# A Joyful Journey: Tungsten(VI) and Tungsten(V) Fluorides Meet *N*‐Heterocyclic Carbenes and Cyclic (Alkyl)(Amino)Carbenes

**DOI:** 10.1002/anie.202504498

**Published:** 2025-05-15

**Authors:** Melanie Riethmann, Leonhard Eyßelein, Ivo Krummenacher, Rüdiger Bertermann, Holger Braunschweig, Michael Gerken, Maik Finze, Udo Radius

**Affiliations:** ^1^ Institute of Inorganic Chemistry Julius‐Maximilians‐Universität Würzburg Am Hubland 97074 Würzburg Germany; ^2^ Institute for Sustainable Chemistry & Catalysis with Boron Julius‐Maximilians‐Universität Würzburg Am Hubland 97074 Würzburg Germany; ^3^ Canadian Centre for Research in Advanced Fluorine Technologies and Department of Chemistry and Biochemistry University of Lethbridge 4401 University Drive Lethbridge AB T1K 3M4 Canada

**Keywords:** Cyclic (alkyl)(amino)carbenes, High oxidation state organometallics, *N*‐heterocyclic carbenes, Transition metal fluorides, Tungsten

## Abstract

The first complexation of tungsten(VI) fluoride with *N*‐heterocyclic carbenes (NHCs) and a cyclic (alkyl)(amino) carbene (cAAC^Me^) is reported, which led to formation of the complexes [(NHC)WF_6_] (NHC = I*i*Pr^Me^, **1**; BI*i*Pr, **2**; IMes, **3**; IDipp, **4**; and SIDipp, **5**) and [(cAAC^Me^)WF_6_] **6**. Solid‐state structural analysis revealed distorted mono‐capped trigonal prismatic geometries for **2** and **3**, whereas **6** adopts a pentagonal bipyramidal coordination. Reduction of **1**–**6** with 0.5 equivalents of TMS‐pyr^Me^‐TMS afforded rare tungsten(V) fluoride complexes [(NHC)WF_5_] (NHC = I*i*Pr^Me^, **7**; BI*i*Pr, **8**; IMes, **9**; IDipp, **10**; and SIDipp, **11**). Reduction of **6** with TMS‐pyr‐TMS yielded the dinuclear, fluoride‐bridged complex [(cAAC^Me^)WF_5_]_2_ **12**. Subsequent addition of a second equivalent cAAC^Me^ to **12** gave the mononuclear, seven‐coordinated bis‐carbene complex [(cAACMe)_2_WF_5_] **13**. Magnetic susceptibility measurements and EPR spectroscopy confirm a predominantly metal‐centered d¹ radical in both **12** and **13**. These findings expand the scope of tungsten fluoride chemistry by providing rare examples of tungsten(VI) and tungsten(V) complexes stabilized by soft carbon‐donor ligands, paving the way for further exploration.

## Graphical abstract

The first tungsten(VI) fluoride complexes coordinated with *N*‐heterocyclic carbenes and cyclic (alkyl)(amino) carbenes were synthesized and characterized. Reduction of these seven‐coordinated complexes afforded rare octahedral tungsten(V) fluoride complexes, a dinuclear fluoride‐bridged complex (cAAC^Me^), or a seven‐coordinated (cAAC^Me^) bis‐carbene complex. These d^0^ tungsten complexes and metal‐centered d¹ radicals expand the scope of tungsten halide chemistry and are convenient entries into tungsten fluoride chemistry in simple organic solvents.

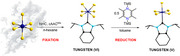

## Introduction

**Figure 1:**
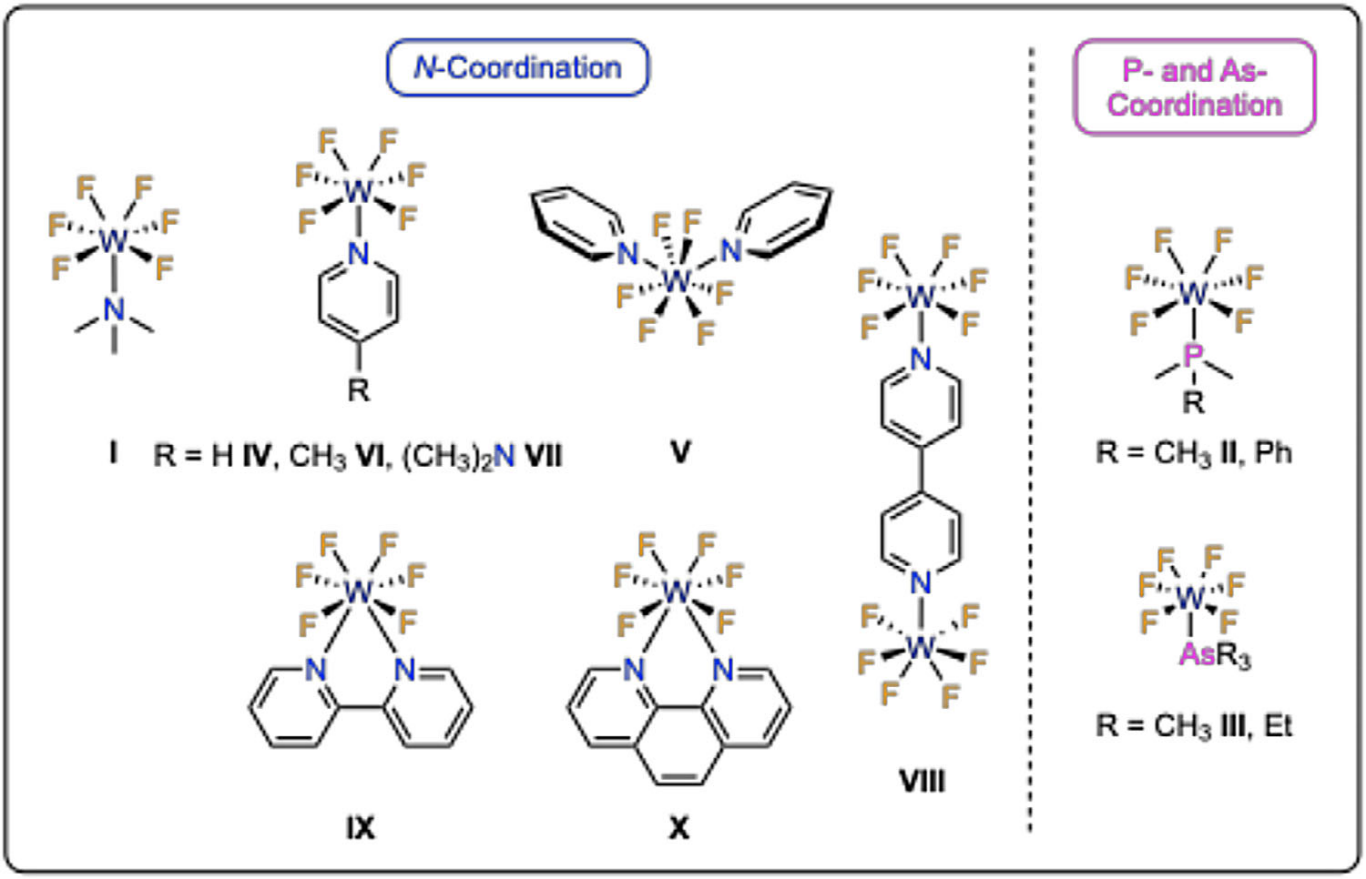
Tungsten hexafluoride complexes with N‐(left), P‐, and As‐coordination (right).

Tungsten hexafluoride^[^ ^1^ ^]^ with tungsten in a d^0^ electronic configuration and the highest possible oxidation state +VI stands out as a highly electrophilic molecular transition metal fluoride. [WF_6_] is a colorless liquid below its boiling point of 17.5 °C and shows an electron affinity (3.5 ± 1 eV)^[^ ^2^, ^3^ ^]^ close to that of elemental fluorine (3.40 eV), but the lowest of the known transition metal hexafluorides. [WF_6_] has the highest vapor pressure among transition metal hexafluorides and has thus found applications in various fields, particularly in the semiconductor sector as a precursor for atomic layer deposition processes.^[^ ^4^, ^5^, ^6^, ^7^ ^]^ Until recently, tungsten hexafluoride was the only transition metal hexafluoride known to form *Lewis* acid/base adducts with organic *Lewis* bases. For example, the reaction of [WF_6_] with pnictogen bases resulted in the formation of seven‐coordinated complexes of the type [({CH_3_}_3_Pn)WF_6_] (Pn = N, **I**; P, **II**; As, **III**, Figure 1) or [(py)WF_6_] **IV** (py = pyridine, Figure 1) and derivatives of it.^[^ ^1^, ^8^, ^9^, ^10^, ^11^, ^12^, ^13^, ^14^, ^15^, ^16^ ^]^ An eight‐coordinated bis‐pyridine adduct [(py)_2_WF_6_] **V** was prepared by addition of [WF_6_] to an excess of pyridine in dichloromethane as a solvent or in pure pyridine. This complex crystallizes in a bicapped trigonal prismatic coordination polyhedron spanned by the six fluorine atoms and both pyridine nitrogen atoms.^[^ ^12^ ^]^ Moreover, further seven‐coordinated complexes [(L)WF_6_] were reported by the Gerken group with 4‐methylpyridine (4‐Mepy), 4‐(dimethylamino)pyridine (DMAP), or 4,4′‐bipyridine (4,4′‐bipy) as ligands to yield [(4‐Mepy)WF_6_] **VI**, [(DMAP)WF_6_] **VII**, and [F_6_W(4,4′‐bipy)WF_6_] **VIII**, respectively. These complexes adopt mono‐capped trigonal‐prismatic geometries, which, aside from pentagonal bipyramidal structures, are the most common geometry observed for coordination number (CN) 7.^[^ ^17^ ^]^ The complex [(py)WF_6_] **IV** was reacted with 1:1 stoichiometric amounts of bidentate *N*‐donor ligands 2,2′‐bipyridine (2,2′‐bipy) and 1,10‐phenathroline (1,10‐phen), which led to pyridine substitution with the formation of the eight‐coordinated complexes [(2,2′‐bipy)WF_6_] **IX** and [(1,10‐phen)WF_6_] **X**. Recently, the Gerken group expanded this chemistry to neutral *Lewis* acid/base adducts of molybdenum hexafluoride and reported the synthesis and characterization of [(py)MoF_6_] and [(py)_2_MoF_6_].^[^ ^18^ ^]^

Whereas several complexes of [WF_6_] are known for “hard”, nitrogen‐based donor ligands, this situation changes for softer *Lewis* bases. Although capped trigonal prismatic [{(CH_3_)_3_P}WF_6_] **II** is known,^[^ ^9^, ^15^ ^]^ the bond between tungsten and phosphorus was considered weak, as the complex revealed detectable dissociation at room temperature. Complex formation was also observed for PEt_3_ and PPh_3_, but these complexes were not characterized properly due to purification and decomposition problems. The triethyl phosphine complex was isolated as an orange oil that decomposes during attempts at distillation, and the triphenyl phosphine complex was obtained as a red solid that was not soluble in organic and inorganic solvents and decomposes above 50 °C.^[^ ^15^ ^]^ Similarly, the reaction of [WF_6_] with other stabilizing donor molecules such as dithioethers RSCH_2_CH_2_SR (R = Me, *i*Pr) led to adduct formation at low temperatures, but the resulting complexes were also not stable toward dissociation at higher temperatures (> ∼210 K).^[^ ^16^ ^]^

Recently, we reported complexes of *N*‐heterocyclic carbenes (NHCs)^[^ ^19^, ^20^, ^21^, ^22^ ^]^ and related cyclic (alkyl)(amino)carbenes (cAACs)^[^ ^23^, ^24^, ^25^, ^26^, ^27^, ^28^, ^29^, ^30^ ^]^ of d‐electron poor transition metals in their higher and highest oxidation states.^[^ ^31^, ^32^, ^33^, ^34^, ^35^ ^]^ As NHCs and related cAAC ligands provide a pronounced steric diversity and electronic flexibility, we were interested whether this class of soft carbon ligands is capable of reacting with tungsten hexafluoride in simple *Lewis* acid/base reactions or if, very likely, electron transfer processes or fluorination reactions prevail in this type of chemistry. Furthermore, it was of interest to us if isolable complexes of [WF_6_] with NHCs and cAACs are suitable for further exploration of tungsten fluoride chemistry. We herein present the first examples of high‐valent seven‐coordinated tungsten(VI) fluoride complexes [(NHC)WF_6_] and [(cAAC^Me^)WF_6_] of NHCs and a cAAC, as well as the first investigations concerning the reduction of such complexes to rather elusive molecular, ligand‐stabilized tungsten(V) fluorides [(L)WF_5_] (L = NHC, cAAC).

## Results and Discussion

### Synthesis and Characterization of W(VI) Complexes

**Scheme 1:**
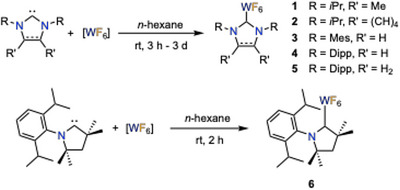
Synthesis of [(NHC)WF_6_] **1–5** and [(cAAC^Me^)WF_6_] **6**.

The reaction of one equivalent of the NHCs I*i*Pr^Me^ (I*i*Pr^Me^ =  1,3‐di‐*iso*‐propyl‐4,5‐dimethyl‐imidazolin‐2‐ylidene}), BI*i*Pr (BI*i*Pr = 1,3‐di‐*iso*‐propyl‐benzimidazol‐2‐ylidene),^[^ ^36^ ^]^ IMes (IMes = 1,3‐dimesitylimidazolin‐2‐ylidene; Mes = 1,3,5‐trimethylphenyl), IDipp (IDipp = 1,3‐bis(2,6‐di‐*iso*‐propylphenyl)imidazolin‐2‐ylidene), SIDipp (SIDipp = 1,3‐bis(2,6‐di‐*iso*‐propylphenyl)imidazolidin‐2‐ylidene), as well as the cyclic (alkyl)(amino) carbene cAAC^Me^ (cAAC^Me^ = 1‐(2,6‐di‐*iso*‐propylphenyl)‐3,3,5,5‐tetramethyl‐pyrrolidin‐2‐ylidene) with stoichiometric amounts of [WF_6_] in *n*‐hexane led to the formation of the complexes [(I*i*Pr^Me^)WF_6_] **1**, [(BI*i*Pr)WF_6_] **2**, [(IMes)WF_6_] **3**, [(IDipp)WF_6_] **4**, [(SIDipp)WF_6_] **5**, and [(cAAC^Me^)WF_6_] **6** as yellow to red solids in excellent yield of 79%–92% (Scheme 1). Key to the successful synthesis was the choice of the solvent, as THF was polymerized under the reaction conditions employed and aromatic solvents (benzene or toluene) led to the unselective formation of multiple products. As **1–6** were only poorly soluble in *n*‐hexane, they precipitated immediately after formation and were thus not available for further decomposition reactions in solution. A similar strategy was applied previously in our titanium(IV) chemistry.^[^ ^31^ ^]^ Once formed, the complexes were isolated by simple removal of all volatiles in a dynamic vacuum or by filtration. For IDipp and SIDipp, the reaction time was extended up to 3 days, as complex formation is rather slow, presumably due to the increased steric demand of the NHC.

Once isolated, the complexes **1–6** are soluble in benzene, toluene, as well as polar solvents such as THF or chloroform without decomposition. The formation of **1–6** was verified by means of ^1^H, ^13^C{^1^H} and ^19^F NMR, IR, and Raman spectroscopy, as well as elemental analysis. In addition, DSC measurements were conducted on a selected set of compounds (see Figures S43–S47 in the Supporting Information). The thermal analyses of [(carbene)WF_6_] were recorded under a nitrogen atmosphere with a heating rate of 10 K·min^−1^ and show surprisingly high decomposition temperatures in a range of ∼170 to 225 °C. [(I*i*Pr^Me^)WF_6_] **1 **is extraordinarily stable, as the onset value of the DSC measurement lies at around 380 °C. The two minor peaks observed below decomposition of **1** could be attributed to crystallization (exothermic effect at ∼120 °C) or melting (endothermic effect at ∼215 °C). The ^1^H and ^13^C{^1^H} NMR spectra are those of NHC‐coordinated, mononuclear complexes, as the resonances were clearly shifted compared to the free NHC, with carbene carbon atom resonances in the range between 198.6 and 266.9 ppm. The low intensity of these resonances was insufficient to reveal the ^183^W satellites, but ^2^ *J*_CF_ coupling (14.5–22.2 Hz) was observed, most indicative of coordination. The ^19^F NMR spectra show only one sharp singlet resonance (except for **6**, s*_br_*) for all fluoride ligands at 151.5 (**1**), 152.2 (**2**), 149.7 (**3**), 151.1 (**4**), 145.6 (**5**), and 140.7 ppm (**6**). These are slightly high‐field shifted compared to uncoordinated [WF_6_] (163.8 ppm) and equipped with ^183^W satellites (^1^ *J*_WF_ coupling constants in the range between 23.1 and 35.8 Hz). It has been demonstrated previously that fluoride ligands in seven‐coordinated complexes [(L)WF_6_] undergo dynamic self‐exchange in solution, which is fast on the NMR time scale.^[^ ^17^ ^]^ However, neither low‐temperature NMR (down to 158 K) nor solid‐state NMR studies resulted in a splitting of these averaged signals (see, for example, Figure S13 in the Supporting Information). The barrier for interconverting the fluoride ligands in these complexes is so low that mobility also occurs rapidly in the solid state (vide infra). The IR spectra of **1–6** reveal characteristic bands for the W─F stretching modes in the region of 658 to 734 cm^−1^, whereas the different signals of the bending vibration could not be separated. For **2**, **3**, and **4**, additional quantum chemical calculations have been carried out, and the results of the calculations are in line with our experimental observations. In their Raman spectra, the frequencies attributed to *ν*(WF) lie between 683 and 711 cm^−1^; the lower stretching frequencies compared to [WF_6_] (772 cm^−1^)^[^ ^37^ ^]^ are due to the increased electron density on tungsten. The frequencies assigned to *ν*(WF) of **1–6** are listed in Table S1 of the Supporting Information.

**Figure 2:**
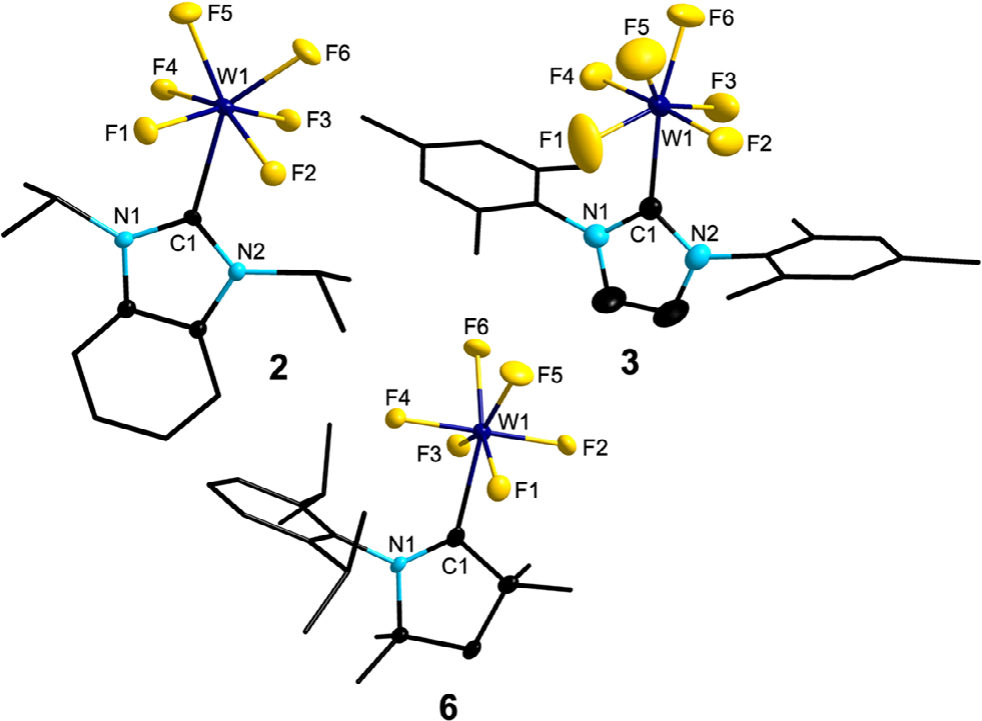
Molecular structures of [(BI*i*Pr)WF_6_] **2** (top left), [(IMes)WF_6_] **3** (top right), and [(cAAC^Me^)WF_6_] **6** (bottom) in the solid state (ellipsoids set at the 50% probability level). Hydrogen atoms are omitted for clarity. For selected bond lengths and angles, see Figures S32, S33, and S35 in the Supporting Information.

The molecular structures of **2**, **3**, and **6** obtained from single crystal X‐ray diffraction (SC‐XRD) (Figure 2) revealed seven‐coordination for the metal centers of [(BI*i*Pr)WF_6_] **2**, [(IMes)WF_6_] **3**, and [(cAAC^Me^)WF_6_] **6**, ligated with six fluorides and one NHC ligand. The coordination sphere around tungsten in **2** and **3** is best described as distorted mono‐capped trigonal prismatic with the carbene carbon atom C1 in the capping position, which results in molecules of pseudo‐*C_s_* symmetry (Figure 2). This is in line with molecular structures of other seven‐coordinated tungsten hexafluoride complexes such as [({CH_3_}_3_P)WF_6_]^[^ ^15^ ^]^ I**I** or [(4‐RC_5_H_4_N)WF_6_] (R = H, **IV**; CH_3_, **VI**; (CH_3_)_2_N, **VII**).^[^ ^17^ ^]^ The W─C distances of 2.264(3) (**2**) and 2.236(5) Å (**3**) are in good agreement with those found in related high‐oxidation state tungsten complexes such as [(IMes)WCl_4_(SEt_2_)] (W─C: 2.217(2) Å) or [(I*i*Pr^Me^)_2_WCl_4_] (C─W: 2.283(2) Å).^[^ ^34^ ^]^ The W─F distances range from 1.828(3) to 1.960(5) Å and are generally elongated in comparison to crystalline [WF_6_] (1.8261(13)–1.8266(19) Å),^[^ ^38^ ^]^ as expected for this higher coordination number. In this context, it is particularly important to highlight *d*(W1─F5) of [(IMes)WF_6_] **2** as it represents the longest tungsten‐to‐fluorine bond with 1.960(5) Å. This is presumably due to an intermolecular interaction evident from SC‐XRD data between the F5 atom and one backbone carbon atom of a neighboring NHC. No distinct trend could be identified regarding the differentiation between the four W─F bonds (F1‐F4) in one plane and the other two bond lengths, W1─F5 and W1─F6, of the mono‐capped prism. However, these distances are in line with those observed for [(py)WF_6_] **IV** (W─F: 1.846(5)–1.880(6) Å)^[^ ^17^ ^]^ or [(py)_2_WF_5_] **V** (W─F: 1.8936(14)–1.9191(11) Å).^[^ ^39^ ^]^ Unlike **2** and **3**, the cAAC complex [(cAAC^Me^)WF_6_] **6** adopts a pentagonal bipyramidal geometry. The W─F distances to the equatorial fluoride ligands are 1.909(4) (W1─F1), 1.904(4) (W1─F3), 1.882(4) (W1─F5), and 1.887(4) Å (W1─F6), while the axial fluoride ligands F2 and F4 are more tightly bound to tungsten with distances of 1.846(4) and 1.835(4) Å, respectively. Notably, the bond lengths of F1 and F3 to the tungsten atom are slightly elongated in the vicinity of the W1─C1 bond compared to those of F5 and F6, suggesting a subtle structural distortion within the equatorial plane of the pentagon. With an F2─W1─F4 angle of 179.61(18)°, the two axial fluorine atoms occupy nearly ideal linear positions.

An important aspect of the chemistry of seven‐coordination is that three different stereoisomers exist, the pentagonal bipyramid (PB), the capped octahedron (CO), and the capped trigonal prism (CTP).^[^ ^40^, ^41^ ^]^ These structures are close in energy and interconvert through minor changes in the angles almost without any activation energy.^[^ ^38^, ^42^ ^]^ It has been shown that in main group heptafluorides and oxo‐hexafluorides, the PB is the preferred geometry, whereas CO or CTP are adopted preferentially by transition metal heptafluoro‐metallates and seven‐coordinated adducts of d^0^ transition metal fluorides. This was attributed to the d‐orbital participation in the σ‐bonding orbitals of these isomers. Therefore, we were interested in a theoretical assessment of our molecules, and the gas‐phase structures of [(BI*i*Pr)WF_6_] **2**, [(IMes)WF_6_] **3**, [(IDipp)WF_6_] **4**, and [(cAAC^Me^)WF_6_] **6** were DFT‐optimized at the PBE0‐D3(BJ)/def2‐TZVP level of theory (see Figure S48 in the Supporting Information). All NHC‐ligated molecules adopt a capped trigonal prismatic geometry with a [WF_6_] trigonal prism and the NHC located in the capping position. There is only a small variation in the W─F distances in a range of 1.867 to 1.880 Å, which agrees well with the averaged experimentally observed distances. The calculated W─C distances of 2.304 (**2**), 2.335 (**3**), and 2.307 Å (**4**) are slightly longer compared to the experimentally observed values of 2.264(3) (**2**) and 2.236 Å (**3**).

**Figure 3:**
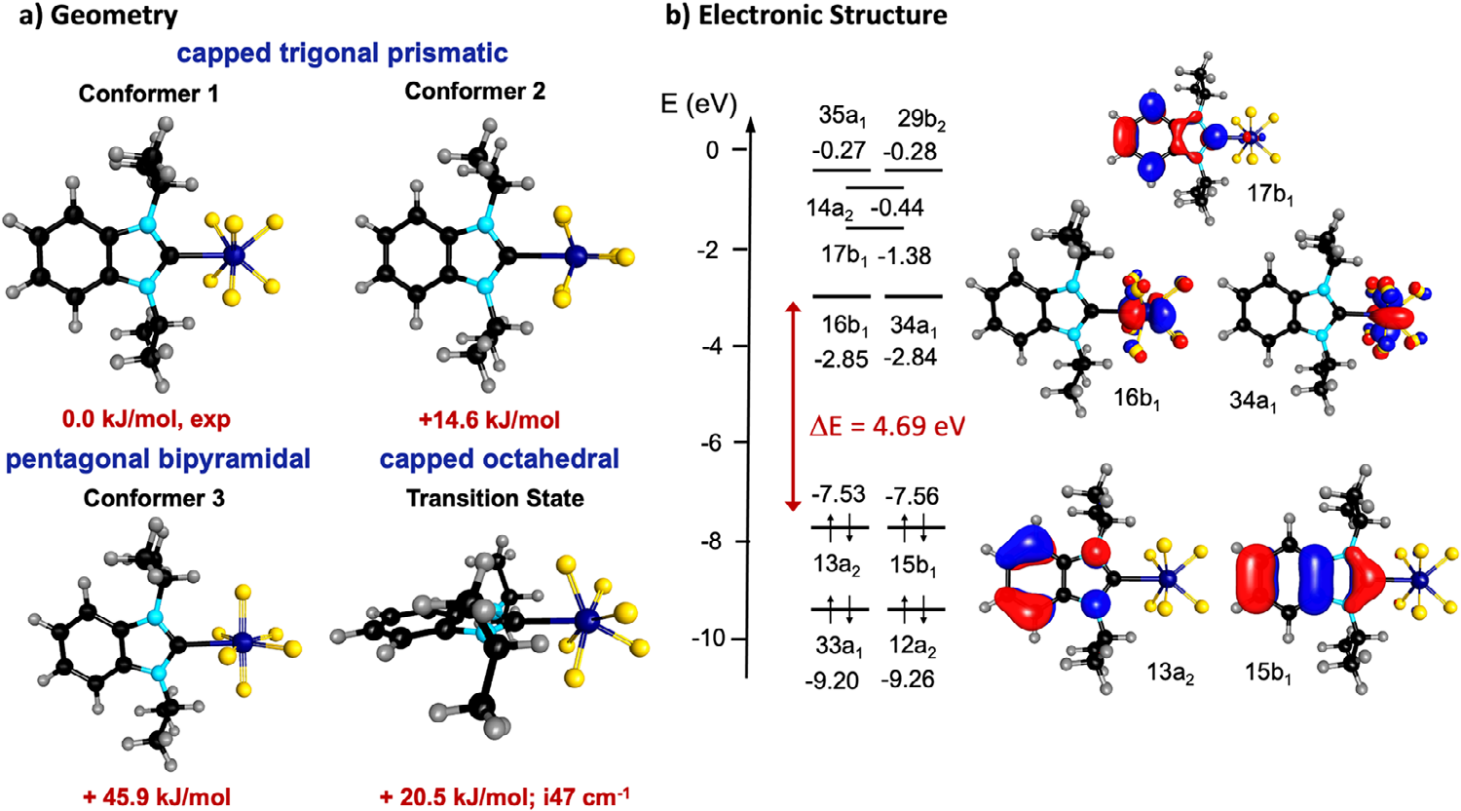
a) DFT‐optimized structures (PBE0‐D3(BJ)/def2‐TZVP) of isomers of [(BI*i*Pr)WF_6_] **2** and b) electronic structure of the CTP energy minimum isomer **2** (conformer 1).

As stated above, the potential hypersurface for seven‐coordination does not contain deep minima corresponding to one polytopal form. This situation reflects the fact that seven points in a sphere cannot be arranged to describe a regular polyhedron and that the number of non‐isomorphic polyhedra with seven vertices is large, but the three high‐symmetry polyhedra PB, CO, and CTP depicted in Figure 3a describe most structures of seven‐coordinated complexes. However, as there are so many possible geometries for seven points on the surface of a sphere, there are many pathways for interconverting these geometries, typically via low energy transition states.^[^ ^43^, ^44^, ^45^, ^46^ ^]^ Non dissociative pathways involve, for example, a turnstile mechanism, where three coordinated fluoride substituents rotate accompanied by a slight simultaneous exchange of other fluoride substituents or a sequential exchange via a pentagonal bipyramidal pathway.^[^ ^43^, ^44^, ^45^, ^46^ ^]^ In order to estimate the barrier for fluoride interconversion of the seven coordinated complexes of the type [(NHC)WF_6_], a transition state was identified for [(BI*i*Pr)WF_6_] **2**, which lies only 20.5 kJ mol^−1^ higher in energy compared to the overall minimum structure (Figure 3a, conformer 1). This transition state can be described as a CO with the NHC in the capping position (Figure 3a). This transition state interconnects two capped trigonal prismatic structures. Due to this low barrier, connected with a rapid fluoride exchange in solution and in the solid state, the NMR spectra of the complexes **1–5** in solution and in the solid state can be explained.

The calculated binding energies between the NHCs and [WF_6_] are 107.9 kJ mol^−1^ for [(BI*i*Pr)WF_6_] **2**, 111.9 kJ mol^−1^ for [(IMes)WF_6_] **3**, and 114.1 kJ mol^−1^ for [(IDipp)WF_6_] **4**. The frontier orbital region of *C*_2_ *_v_*‐symmetric **2** is shown on the right side of Figure 3. The highest occupied frontier orbitals, 13a_2_ and 15b_1_, which are almost degenerate at −7.53 and −7.56 eV, are *π*‐orbitals of the imidazole aromatic system. The lowest unoccupied frontier orbitals are either tungsten d in character, such as the orbitals 16b_1_ at −2.85 eV and 34a_1_ at −2.84 eV, or π* orbitals of the benzimidazole ring system, such as the carbene *π* orbital 17b_1_ at −1.38 eV. The HOMO–LUMO gap is 4.69 eV, which leads to UV/Vis absorptions in the UV region and the pale yellow to white color of these materials. This was also confirmed by TD‐DFT calculations (*ω*B97X/def2‐TZVP) on [(BI*i*Pr)WF_6_] **2** and [(IDipp)WF_6_] **4**, where no excitations were predicted in the visible region.

**Figure 4:**
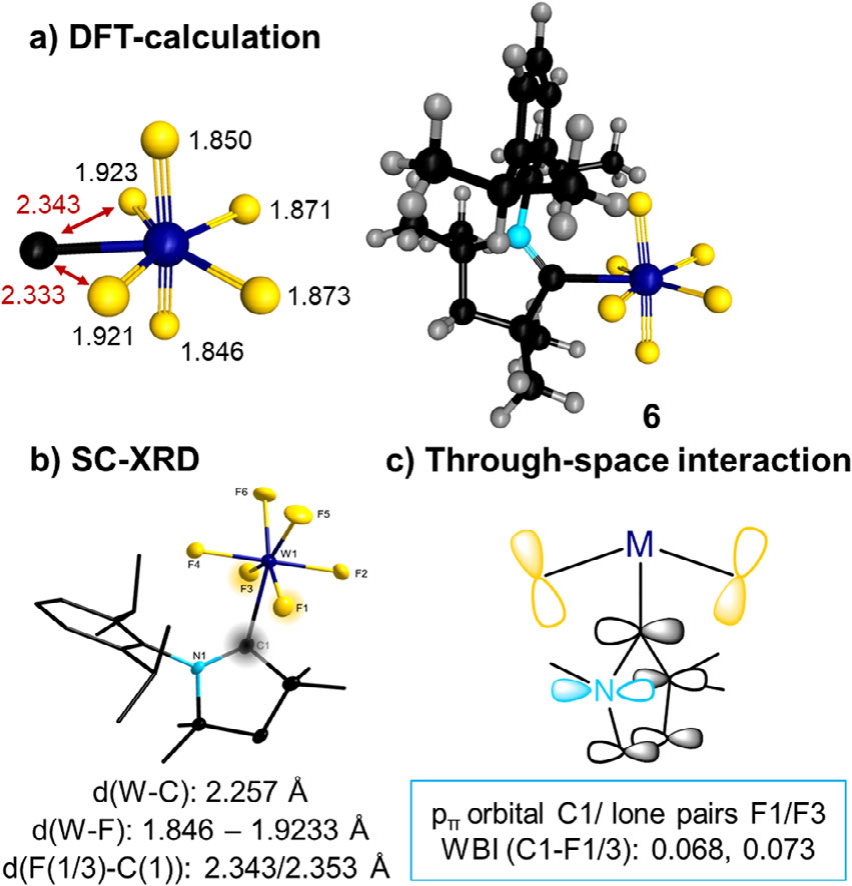
a) DFT‐optimized structure (PBE0‐D3(BJ)/def2‐TZVP) of [(cAAC^Me^)WF_6_] **6**; b) SC‐XRD structure of **6**; c) through space interaction model.

The complex [(cAAC^Me^)WF_6_] **6** optimizes to a pentagonal bipyramidal geometry, which confirms the results obtained from SC‐XRD (Figures 2 and 4). The cAAC ligand is located in the pentagonal plane of the coordination polyhedron, which is typically associated with the sterically more encumbered site of the PB. The calculated W─C bond length is 2.257 Å, which matches exactly the experimentally observed value. The W─F distances optimize pairwise to 1.846/1.850 Å for the axial fluoride ligands, 1.871/1.873 Å for the equatorial fluoride ligands opposite to the cAAC ligand, and 1.921/1.923 Å for the equatorial fluoride ligands adjacent to the carbene carbon atom. This W─F elongation of roughly 0.5 Å results in a relatively short distance to the carbene carbon atom (2.333 and 2.343 Å, Figure 4). A through‐space C_carbene_─F interaction between occupied p‐orbitals at the fluoride ligands F1 and F3 (fluoride lone pairs, Figure 4c) and the unoccupied p_π_‐orbital of the carbene carbon atom was corroborated by the notably short C_carbene_─F1/F3 distances (2.343(8)/2.353(8) Å) obtained from SC‐XRD. This interaction stabilizes the PB structure of **6**, and the analysis of Wiberg bond indices (0.068 and 0.073) indeed indicates a small orbital interaction between these atoms. Similar through‐space interactions were observed for related early transition metal halide complexes such as [(cAAC^Me^)TiCl_4_]^[^ ^31^ ^]^ or [(IMes)TiCl_2_(NMe_2_)].^[^ ^47^ ^]^

### Synthesis and Characterization of W(V) Complexes

The majority of NHC tungsten complexes are those of a low‐valent tungsten atom in oxidation states 0 to +III, most of which contain carbonyl co‐ligands.^[^ ^48^, ^49^ ^]^ A limited number of tungsten complexes in intermediate and high oxidation states are known, which predominantly bear chlorido and oxo ligands.^[^ ^34^, ^50^, ^51^, ^52^ ^]^ Thus, the high‐yield preparation of the NHC and cAAC tungsten(VI) fluoride complexes presented above offers a convenient route for the development of tungsten fluoride chemistry. As a first application of these complexes in molecular synthesis, we envisaged the preparation of carbene‐stabilized tungsten(V) fluorides, as the preparation and fixation of thermally unstable [WF_5_] is challenging. In 1968, Schröder and Grewe first introduced an elaborated and highly specialized reduction of [WF_6_] with tungsten wires at 500–700 °C in a cooled quartz ampoule, enabling the isolation of yellow crystalline neutral [WF_5_] for the first time.^[^ ^53^, ^54^ ^]^ Later on, an alternative synthetic process was established through a *Lewis* acid/base reaction by fluoride abstraction from [NaWF_6_] in an HF/SbF_5_ medium, followed by sublimation at 0 °C.^[^ ^55^ ^]^ Most recently, Kraus et al. synthesized [WF_5_] by photochemical reduction of [WF_6_] utilizing dihydrogen as a reducing agent and Hg as a photosensitizer at 254 nm.^[^ ^56^ ^]^

First complexes of [W^VI^F_5_]^+^ were reported by one of us.^[^ ^57^ ^]^ Fluoride abstraction from the tungsten(VI) complexes **IX** and **X** (Figure 1) using SbF_5_OSO/SO_2_ resulted in the formation of [(L)WF_5_]^+^ (L = 2,2′‐bipy, 1,10‐phen). Similarly, monocationic [(py)_3_WF_5_]^+^ was isolated by fluoride abstraction starting from [(py)_2_WF_6_] **V** and [TMS(py)][OTf] in dichloromethane. This cationic species was subsequently reduced with pyridine to yield the first neutral tungsten(V) fluoride complex [(py)_2_WF_5_].^[^ ^39^ ^]^

**Scheme 2:**
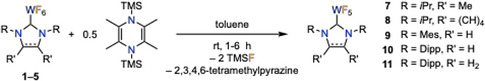
Synthesis of [(NHC)WF_5_] **7–11**.

For the reduction of the complexes **1–6**, we decided not to split the synthesis into fluoride abstraction and subsequent electron transfer but to treat these complexes with 0.5 equivalents of the non‐metallic reducing agent 2,3,5,6‐tetramethyl‐1,4‐bis(trimethylsilyl)‐1,4‐dihydropyrazine (TMS‐pyr^Me^‐TMS)^[^ ^58^ ^]^ This reaction afforded the tungsten(V) complexes [(I*i*Pr^Me^)WF_5_] **7**, [(BI*i*Pr)WF_5_] **8**, [(IMes)WF_5_] **9**, [(IDipp)WF_5_] **10**, and [(SIDipp)WF_5_] **11** in yields of 47%–81% as light yellow to off‐white powders (Scheme 2). The successful synthesis of **7–11** can be ascertained most easily by the release of two equivalents of TMSF and a sharp singlet in the ^1^H NMR spectrum at 2.24 ppm (in C_6_D_6_) for the 2,3,5,6‐tetramethylpyrazine generated during the reaction.

These NHC complexes were characterized using ^1^H and ^13^C{^1^H} NMR spectroscopy, IR and Raman spectroscopy, as well as elemental analysis. In contrast to **1–6**, the complexes **7–11** can be identified via HRMS. Compared to the bright yellow to red starting materials, the tungsten(V) fluoride complexes **7–11** are pale yellow to off‐white. Furthermore, solubility in nonpolar solvents such as benzene or toluene decreases noticeably, and decomposition of the material in polar or halogenated solvents such as THF or dichloromethane was observed over a period of days. Despite the paramagnetic nature of these d^1^ complexes, their ^1^H NMR and ^13^C{^1^H} NMR spectra show relatively conclusive signals, which do not reveal extreme paramagnetic shifts or paramagnetic broadening. Exceptions are the atoms directly attached to the d^1^ metal center, which cannot be detected. The absence of NMR signals for the carbene carbon and the fluoride atoms in the inner coordination sphere is an indication of the paramagnetic nature of the tungsten(V) center in **7–11**. However, the methine protons of the *iso*‐propyl groups of [(I*i*Pr^Me^)WF_5_] **7** give rise to a broadened septet, heavily downfield shifted from 5.15 ppm (**1**) to 10.99 ppm (**7**) in benzene‐*d*_6_ (see Figures S1, S20, and S21 in the Supporting Information). Unfortunately, determination of the magnetic moment in solution (in either benzene, toluene, or 1,2‐difluorobenzene) using Evans’ method^[^ ^59^ ^]^ failed for **7–11**, and EPR measurements performed on **7**, **10**, and **11** showed no resonance in standard X‐band (9.38 GHz) EPR spectroscopy, likely due to rapid relaxation resulting from the near‐degeneracy of the t_2g_‐orbitals (vide infra).^[^ ^60^ ^]^ Similarly, no resonance was observed previously for [(py)MoF_5_]_2_, whereas an EPR signal was easily detectable for the [(py)_2_MoF_5_] adduct.^[^ ^61^ ^]^

**Figure 5:**
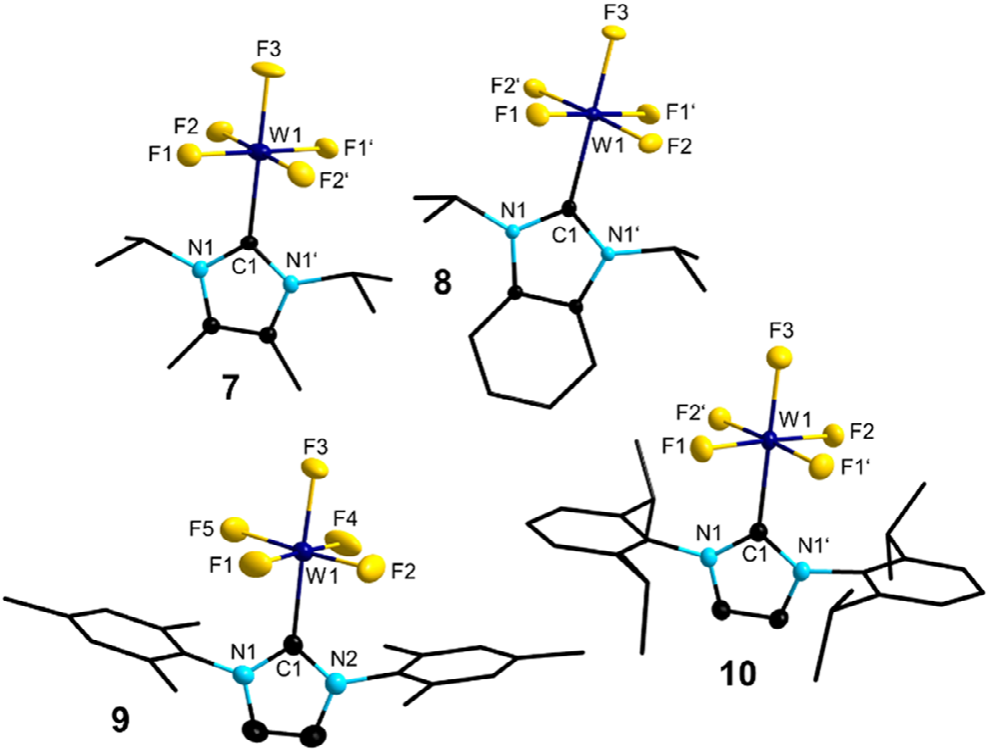
Molecular structures of [(I*i*Pr^Me^)WF_5_] **7** (top, left), [(BI*i*Pr)WF_5_] **8** (top, right), [(IMes)WF_5_] **9** (bottom, left), and [(IDipp)WF_5_] **10** (bottom, right) (ellipsoids set at the 50% probability level). Hydrogen atoms are omitted for clarity. For selected bond lengths and angles, see Figures S36–S39 and Table S2 in the Supporting Information.

The molecular structures of **7**, **8**, **9**, and **10** are presented in Figure 5. Notably, complexes **7–11** are among the few crystallographically characterized tungsten(V) complexes WX_5_L (X = F, Cl, Br; L = neutral electron donor ligand) ever reported.^[^ ^62^, ^63^ ^]^ The environment around W in **7**, **8**, **9**, and **10** is close to octahedral, with the NHC and one of the fluoride ligands (F3) located in the “axial” positions and the four remaining fluorides in “equatorial” positions, staggered with respect to the NHC core plane. The W─F distances to the equatorial fluorides (1.814 to 1.879 Å) are unexceptional, but W1─F3 are noteworthily elongated (1.899(2)–1.928(4) Å) due to the excellent donor properties of the *trans*‐located NHC ligands. The W1─C1 distances range from 2.188(3) to 2.215(6) Å and are slightly shorter as observed for d^0^‐[(NHC)WF_6_] complexes **2** and **3** (2.236(5)–2.264(3) Å). The shortest intramolecular hydrogen‐to‐fluorine contacts are between the methine protons of the isopropyl groups of **7**, **8**, and **10** (2.302(2)–2.700(3) Å) (see, for example, Figure S36 in the Supporting Information) and between one proton of the *ortho* methyl groups of **9** (2.374(3)–2.385(3) Å) with the equatorial fluorides, which suggests only weak or no H···F interactions.

**Figure 6:**
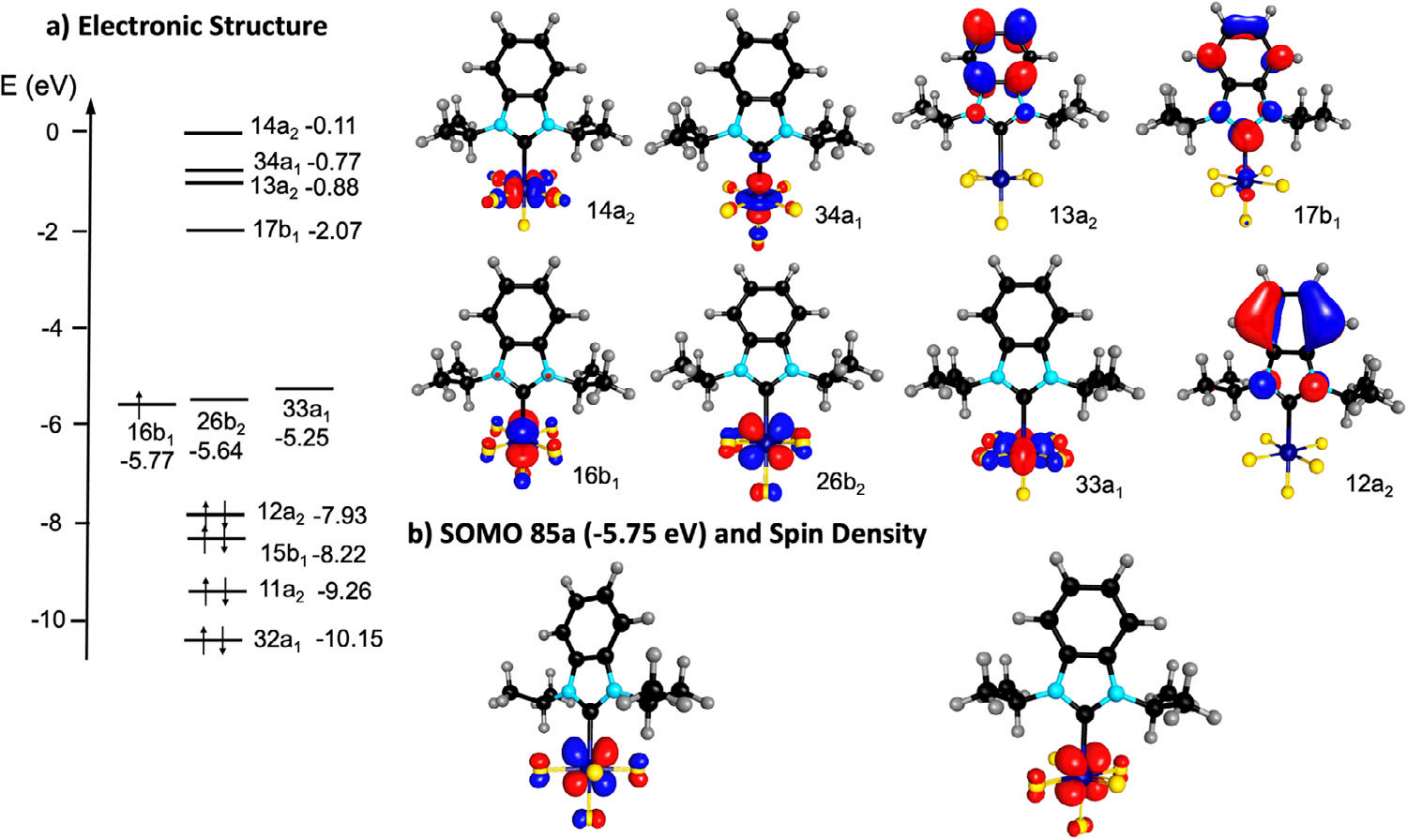
Electronic structure, SOMO and spin density of C_2v_‐[(BI*i*Pr)WF_5_] **8_C_2v_**.

The geometries of [(I*i*Pr^Me^)WF_5_] **7**, [(BI*i*Pr)WF_5_] **8**, [(IMes)WF_5_]** 9**, and [(IDipp)WF_5_] **10** were DFT‐optimized at the PBE0‐D3(BJ)/def2‐TZVP level of theory (see Figure S50 in the Supporting Information), and the optimizations confirm a distorted octahedral structure in which the fluoride ligands of the equatorial plane are pairwise distorted toward and away from the carbene ligands. The geometry of [(BI*i*Pr)WF_5_] **8** has also been optimized with *C*_2_ *_v_*‐symmetry restraints (**8_C_2v_**), which forces all fluoride substituents in ideal octahedral positions. This undistorted octahedral geometry is a transition state for the interconversion of the inequivalent fluoride ligands located in the equatorial plane. This transition state lies only 2.7 kJ mol^−1^ higher in energy than the ground‐state minimum structure of **8**. The electronic structure of **8_C_2v_** is presented in Figure 6.

The molecular orbital 16b_1_ at 5.77 eV is the singly occupied molecular orbital (SOMO) of this complex and belongs to the three tungsten‐centered d‐orbitals (d_xy_, d_yz_, d_xy_) of the t_2__g_ set of the regular octahedron. Below that, orbital 12a_2_ at −7.93 eV is a *π*‐orbital of the imidazole aromatic system. Between the t_2g_‐type orbitals and the orbitals 34a_1_ (d_z2_) and 14a_1_ (d_x2‐y2_), the e_g_ orbitals of a regular octahedron, are the carbene *π*‐orbital 17b_1_ and another *π* ^*^‐orbital 13a_2_ of the imidazole ring. The SOMO of the unrestrained optimized complex **8** is the orbital 85a at −5.75 eV, which makes the only contribution to the overall, metal‐centered spin density of the complex (Figure 6b). The SOMO of the complex reveals some antibonding *π*‐interaction of the singly occupied d orbital and three fluoride ligands, one in *trans* position to the NHC and two mutually in *trans* position in the equatorial plane. The latter are these fluoride ligands, which show slightly elongated tungsten fluoride contacts (see above). For each of the complexes [(I*i*Pr^Me^)WF_5_] **7**, [(BI*i*Pr)WF_5_] **8**, [(IMes)WF_5_]** 9**, and [(IDipp)WF_5_] **10** the spin density has been calculated (see Figure S51 in the Supporting Information), and they all show metal‐centered spin orbitals and spin densities, in which a single electron is located in a d‐centered orbital as shown for **8**. TD‐DFT calculations performed for [(BI*i*Pr)WF_5_] **8**, [(IMes)WF_5_]** 9**, and [(IDipp)WF_5_] **10** reveal energetically low lying d‐d transitions within the t_2g_ block with low oscillator strengths at 2899 nm (**8**), 3133 nm (**9**), and 3116 nm (**10**) as well as 1925 nm (**8**), 5465 nm (**9**), and 5439 nm (**10**), which correspond to transitions of 16b_1_ to 26b_2_ or 33a_1_ for **8_C_2v_** (Figure 6a), but no absorptions in the visible region. This explains the off‐white color of the complexes.

It has been shown previously that d^1^ complexes in an octahedral ligand field with the ground state ^2^T_2g_ (one d electron in the t_2g_ set of d‐ orbitals) reveal considerable spin‐orbit coupling and typically fast relaxation.^[^ ^60^, ^64^ ^]^ For **8**, and for the complexes [(NHC)WF_5_] **7**–**10** in general, the unpaired tungsten d^1^ electron is located in a quasi‐degenerate orbital in the manifold from the octahedral t_2g_ set, 16b_1_, 26b_2_, and 33a_1_ (Figure 6). This most probably induces fast relaxation, which may rationalize the relatively sharp and little shifted paramagnetic NMR spectra as well as the absence of EPR resonances in these six‐coordinated octahedral complexes. We did not observe EPR resonances for the octahedral complexes [(NHC)WF_5_] **7**–**10**, whereas a resonance was detected for seven‐coordinated [(cAAC^Me^)WF_5_]_2_ **12** and [(cAAC^Me^)_2_WF_5_] **13** (vide infra). Similar observations have been made recently by one of us for *Lewis* base adducts of MoF_5_, where no EPR resonance was detected for [(CH_3_CN)MoF_5_] (CN = 6), but [(py)_2_MoF_5_] (CN = 7) was EPR active.^[^ ^61^ ^]^ Considering simple crystal field theory for a pentagonal bipyramidal polyhedron D_5h_‐ML_7_, the d‐orbital splitting is d_xz_ and d_yz_ (e_1_″; −5.28 Dq) below $d_{x^{2}-y^{2}}$ and d_xy_ (e_2_′, +2.82 Dq) below $d_{z^{2}}$ (a_1_′, +4.93 Dq). Regarding the DFT calculated electronic structure of **13** (see Figure S52 in the Supporting Information), the unpaired electron is located in an energetically isolated orbital 188 a(α), essentially a tungsten‐centered d_xz_ orbital. The energy gap calculated in an unrestricted calculation between the spin orbital SOMO 188 a(α) and the unoccupied spin orbital 189 a(α) is 3.42 eV. Thus, occupation of a single electron in an energetically isolated orbital in complexes of CN = 7 should lead (at least in comparison to octahedral complexes of CN = 6) to slower relaxation of the electron spin magnetization and thus to paramagnetically shifted NMR resonances and detectable EPR signals.^[^ ^65^ ^]^ Similarly, dimeric [(cAAC^Me^)WF_5_]_2_ **12**, seven‐coordinated at tungsten, also shows energetically isolated frontier orbitals (see Figure S53 of the Supporting Information), which results in much slower relaxation, and EPR resonances were thus detectable (vide infra).

**Scheme 3:**
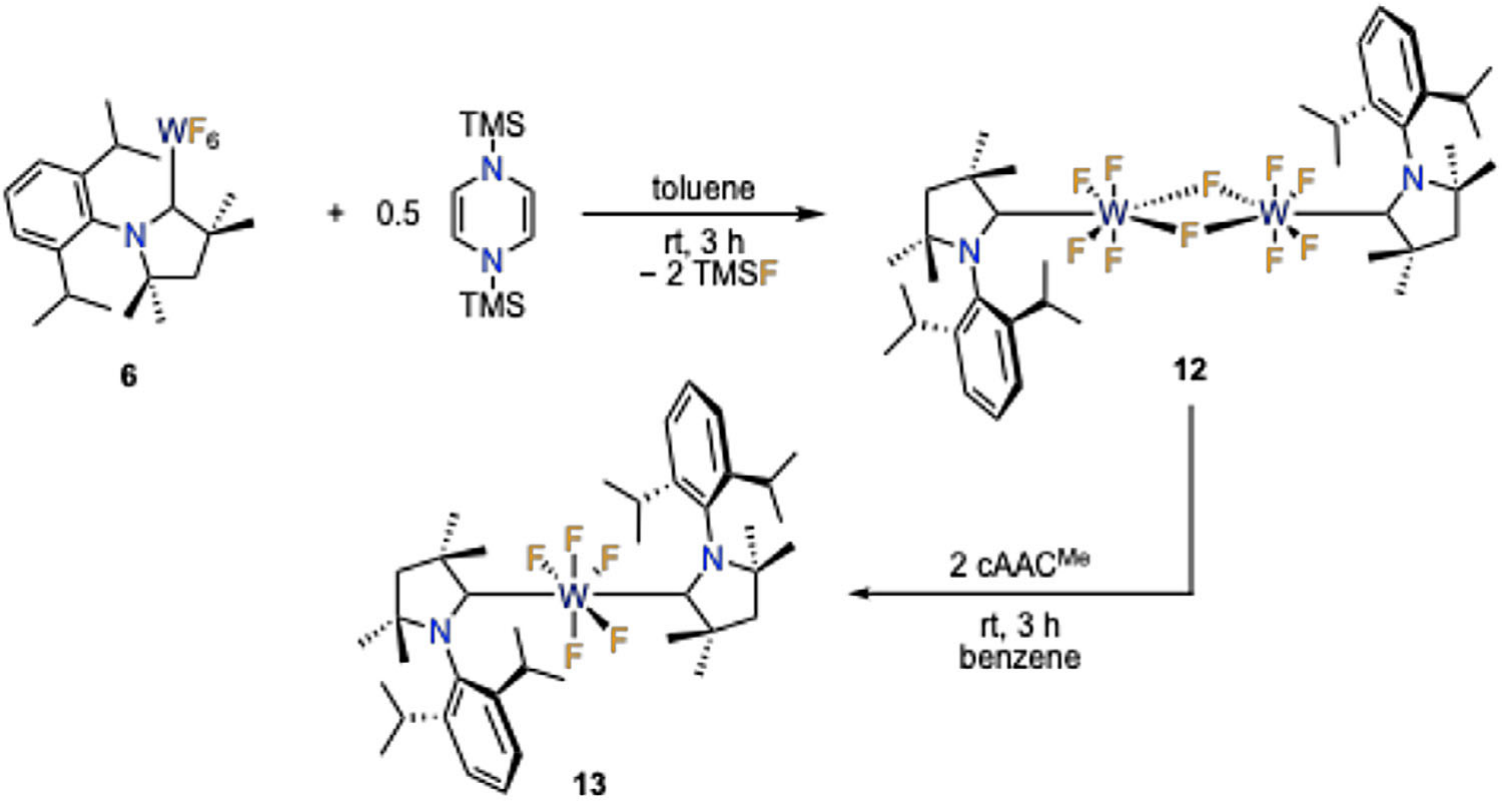
Synthesis of [(cAAC^Me^)WF_5_]_2_ **12** and subsequent reaction with cAAC^Me^ to yield [(cAAC^Me^)_2_WF_5_] **13**.

**Figure 7:**
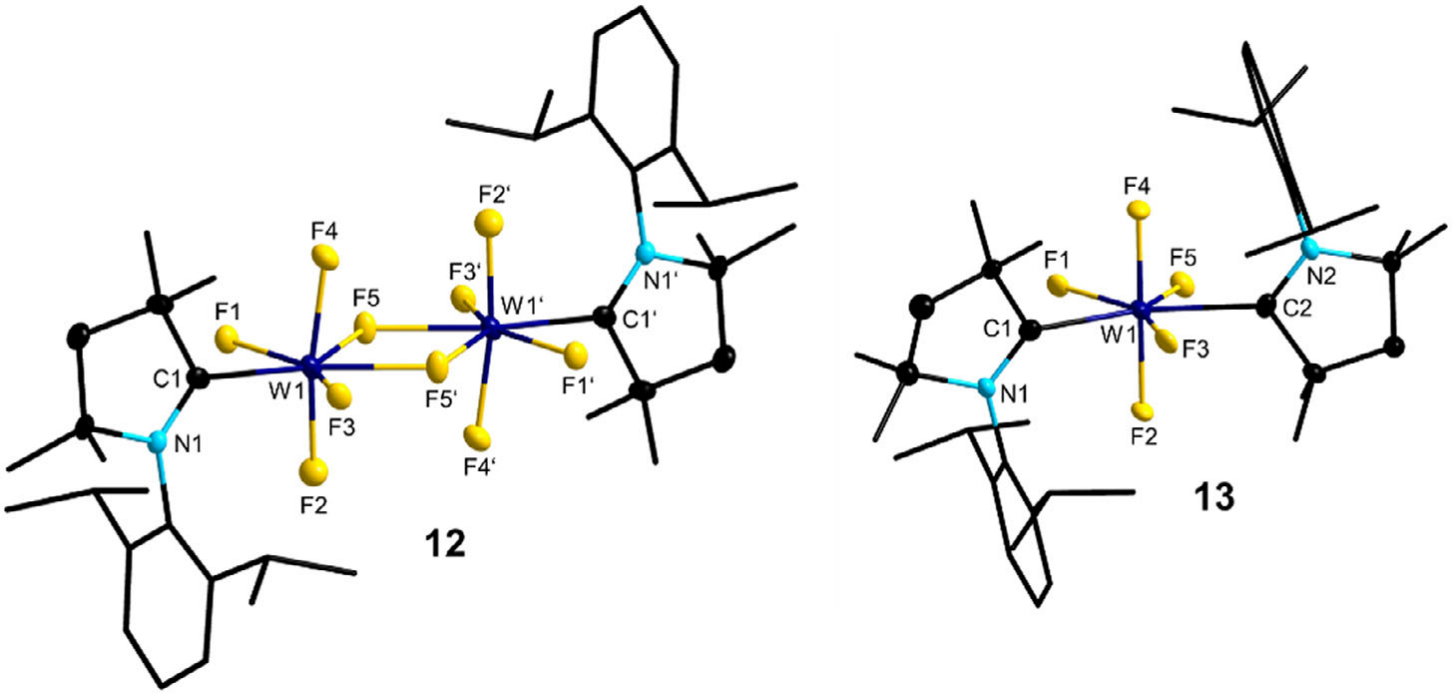
Molecular structures of [(cAAC^Me^)WF_5_]_2_ **12** (left) and to [(cAAC^Me^)_2_WF_5_] **13** (right) (ellipsoids set at the 50% probability level). Hydrogen atoms are omitted for clarity. For selected bond lengths and angles, see Figures S40 and S41 in the Supporting Information.

As we were unable to isolate [(cAAC^Me^)WF_5_] via reduction of [(cAAC^Me^)WF_6_] **6 **with TMS‐pyr^Me^‐TMS, the more reactive derivative 1,4‐bis(trimethylsilyl)‐1,4‐dihydropyrazine (TMS‐pyr‐TMS) was used as a potential alternative reducing agent.^[^ ^66^, ^67^ ^]^ [(cAAC^Me^)WF_6_] **6 **was dissolved in toluene, and upon the addition of 0.5 equivalents of TMS‐pyr‐TMS to the initially orange solution, an instantaneous color change to deep dark purple was observed (Scheme 3, top). After removal of all volatiles after 15 min, ^1^H NMR studies of the remaining dark blue powder in benzene‐*d*_6_ only revealed several signals of low spectroscopic value. However, crystallization of the reaction mixture afforded the fluoride‐bridged dimeric (at least in the solid‐state) complex [(cAAC^Me^)WF_5_]_2_ **12**, which was identified by SC‐XRD (Figure 7, left).

**Figure 8:**
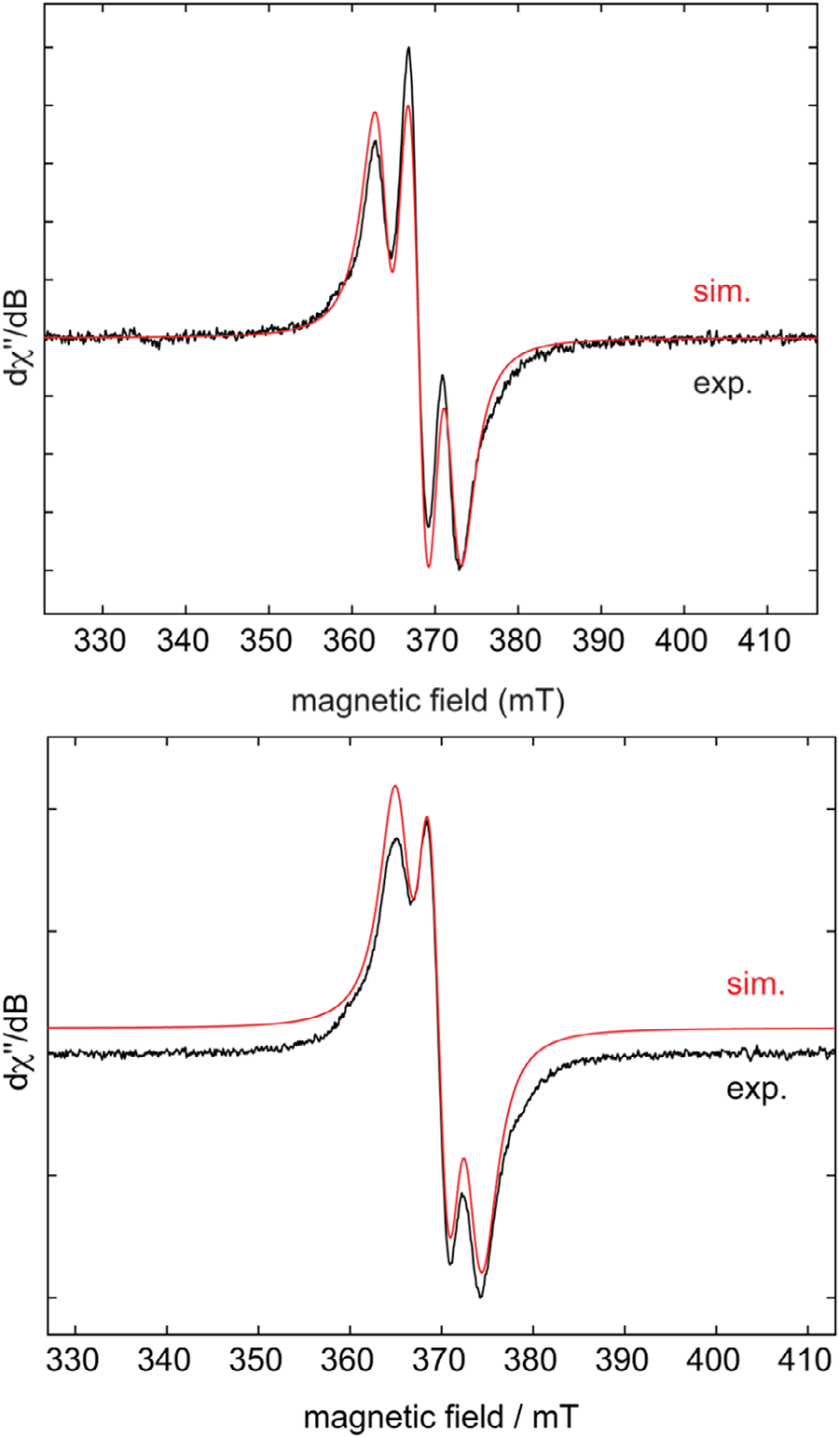
Experimental (black) and simulated (red) X‐band EPR spectra of [(cAAC^Me^)WF_5_]_2_ **12** (top) and [(cAAC^Me^)_2_WF_5_] **13** (bottom) in benzene solution at room temperature. The EPR simulations involve an isotropic *g* factor of 1.821 (**12**) and 1.814 (**13**) and fluorine hyperfine couplings of *a*(^19^F, 2F) = 101 MHz (36 G) (**12**) and *a*(^19^F, 2F) = 89 MHz (31.8 G) and *a*(^19^F, 3F) = 19 MHz (6.8 G) (**13**).

The molecular structure reveals the formation of a fluoride‐bridged dimer [(cAAC^Me^)WF_5_]_2_ **12**, in which each W^v^ atom reaches a distorted pentagonal bipyramidal surrounding. The W─C1 distance of 2.209(5) Å is in line with W─C1 bond lengths observed for **4–6** (Figure 3, Table S1), as are W─F distances of **12** (1.834(3)–1.904(3) Å). The fluoride bridge is within standard deviations symmetric, showing W─F bond lengths of 2.093(3) Å (W1─F5) and 2.104(3) Å (W1─F5’). These 3c–4e^−^ *µ*‐F bond lengths are close to those observed in the related complex [MoF_5_(NC_5_H_5_)]_2_ (Mo−*µ*‐F: 2.0740(12) and 2.0786(11) Å)^[^ ^61^ ^]^ or [Na‐15‐crown‐5]_2_[WO_2_F_3_]·2 CH_3_CN (W−*µ*‐F: 2.151(4) and 2.092(5) Å).^[^ ^68^ ^]^ Although the tungsten to tungsten separation (W1···W1’: 3.5117(4) Å) is smaller than the sum of the van der Waals radii (*r*_vDW_(W) = 2.1 Å),^[^ ^69^ ^]^ a strong bond between both atoms accompanied by pairing of the tungsten d^1^ electrons can be excluded. Complex **12** was additionally characterized by means of elemental analysis, IR and Raman spectroscopy, magnetometry in solution, and EPR spectroscopy (Figure 8, left). The EPR spectrum of **12** recorded in benzene solution revealed an isotropic *g*‐tensor of 1.821 and a three‐line pattern most likely corresponding to coupling of the unpaired electron to two equatorial fluorine atoms with *a*(^19^F, 2F) = 101 MHz (36 G). Using the Evans’ method, the magnetic moment of **12** in benzene‐*d*_6_ solution was determined to be *µ*_eff_ = 1.57 *µ*_B_, significantly lower than the spin‐only value for a d^1^ complex with a single unpaired electron (*µ*_eff_ = 1.73 *µ*_B_). This discrepancy arises from the expected spin‐orbit coupling associated with the heavy metal center tungsten.

Treatment of the dimeric complex **12** with two equivalents of cAAC^Me^ led to a deep, dark red solution, and the paramagnetic bis‐cAAC^Me^ complex [(cAAC^Me^)_2_WF_5_] **13** was isolated and characterized. UV/Vis spectroscopy of the intensively colored complex **13** revealed two absorption bands in the UV range at 220 and 260 nm, along with a maximum in the visible range at 438 nm (see Figure S42 in the Supporting Information; TD‐DFT calculated absorption at 463 nm). The molecular structure of [(cAAC^Me^)_2_WF_5_] **13** (Figure 7, right) confirms a pentagonal bipyramidal geometry as observed for the dinuclear complex [(cAAC^Me^)WF_5_]_2_ **12** or the seven‐coordinated complex [(cAAC^Me^)WF_6_] **6**, in which both cAAC^Me^ ligands occupy equatorial positions. PB complexes with neutral donor molecules in non‐adjacent equatorial sites seem to be preferentially formed for transition metals with a low electron count, as such coordination polyhedra were also observed for the W^VI^ complex [(py)_2_WOF_4_],^[^ ^70^, ^71^ ^]^ the W^V^ complex [(py)_2_WF_5_] **V**,^[^ ^39^ ^]^ and the W^IV^ complex [(C_6_F_5_N)(py)_2_WF_4._]^[^ ^71^ ^]^ However, the complexes [(cAAC^Me^)WF_5_]_2_ **12** and [(cAAC^Me^)_2_WF_5_] **13** represent one of the few seven‐coordinated tungsten(V) fluoride complexes, aside from [(py)_2_WF_5_] **V** and ionic K_2_[WF_7_].^[^ ^72^ ^]^

The experimentally observed magnetic moment of **13** in benzene‐*d*_6_ solution is 1.84 *µ*_B_ (Evans’ method)^[^ ^59^ ^]^ which corresponds well with the expected theoretical value of 1.73 *μ*_B_ of one single unpaired electron of the tungsten d^1^ complex. The EPR spectrum of **13** (Figure 8) displays a broadened triplet signal (*g* = 1.814) from which two distinct fluorine hyperfine couplings of *a*(^19^F, 2F) = 89 MHz (31.8 G) and *a*(^19^F, 3F) = 19 MHz (6.8 G), respectively, can be determined via spectral simulation. The low *g*‐factor indicates that the unpaired electron is primarily localized on the tungsten atom.

## Conclusion

We report herein the complexation of gaseous tungsten(VI) fluoride at room temperature by using different *N*‐heterocyclic carbenes as well as a cyclic (alkyl)(amino)carbene cAAC^Me^ to yield the solid complexes [(NHC)WF_6_] (NHC  =  I*i*Pr^Me^, **1**; BI*i*Pr, **2**; IMes, **3**; IDipp, **4**; SIDipp, **5**) and [(cAAC^Me^)WF_6_] **6**, respectively, in overall good yields. The use of hexane as a solvent is crucial to circumvent decomposition reactions. The solid‐state molecular structures of **2** and **3** confirm seven‐coordination in the form of distorted capped trigonal prisms, with the carbene ligand adopting capping positions. In contrast, the cAAC complex [(cAAC^Me^)WF_6_] **6** adopts a pentagonal bipyramidal geometry, which represents the first example of a transition metal hexafluoride adduct with that geometry. The compounds **1–6** are the first examples of tungsten(VI) halide complexes stabilized by a soft carbon two valence electron (2 VE) donor ligand. Reaction of [(carbene)WF_6_] **1–6** with 0.5 equivalents of the mild, non‐metallic reducing reagent TMS‐pyr^Me^‐TMS resulted in the formation of rare *Lewis*‐base stabilized tungsten(V) fluoride complexes [(NHC)WF_5_] (NHC  =  I*i*Pr^Me^, **7**; BI*i*Pr, **8**; IMes, **9**; IDipp, **10**; SIDipp, **11**). As this approach failed for [(cAAC^Me^)WF_6_] **6**, the stronger reducing reagent TMS‐pyr‐TMS was used, which led to isolation of dinuclear, fluoride‐bridged [(cAAC^Me^)WF_5_]_2_ **12**. The fluoride bridges can be broken upon addition of a second equivalent of cAAC^Me^ to yield the bis‐carbene complex [(cAAC^Me^)_2_WF_5_] **13**. Magnetometry in solution, EPR, and UV/Vis spectroscopy (for **13**) experiments performed on the complexes **12** and **13** are indicative of a predominantly metal‐centered paramagnetic d^1^ radical in both cases. The compounds **7–13** are some of the few d^1^ tungsten penta‐halide complexes of the type [WX_5_(L)] (L = 2 VE donor) and represent the first tungsten(V) pentahalide complexes stabilized by a carbene ligand, and thus expand the scope of hitherto limited known tungsten(V) halide, especially fluoride, complexes. Herein we have demonstrated that (i) NHCs and cAACs are suitable ligands to enter high oxidation state metal fluoride chemistry without any degradation, such as oxidative fluorination of the carbene; (ii) such highly fluorinated complexes of W(VI) and W(V) are available in high yield for further reactivity studies; and that (iii) these adducts of highly electrophilic fluorides are now accessible for chemical reactions in standard organic solvents.

## Supporting Information

Crystallographic data for the structures reported in this paper have been deposited with the Cambridge Crystallographic Data Centre as supplementary publication no.s CCDC‐2425821 (**2**), CCDC‐2425823 (**3**), CCDC‐2425822 (**6**), CCDC‐2425817 (**7**), CCDC‐2425816 (**8**), CCDC‐2425819 (**9**), CCDC‐2425820 (**10**), CCDC‐2425824 (**12**), CCDC‐2425818 (**13**). Crystal data can be obtained free of charge from The Cambridge Crystallographic Data Centre via www.ccdc.cam.ac.uk/data_request/cif ^[^ ^73^ ^]^ The authors have cited additional references within the Supporting Information.^[^ ^23^, ^66^, ^67^, ^74^, ^75^, ^76^, ^77^, ^78^, ^79^, ^80^, ^81^, ^82^, ^83^, ^84^, ^85^, ^86^, ^87^, ^88^, ^89^, ^90^, ^91^, ^92^, ^93^, ^94^, ^95^, ^96^, ^97^, ^98^, ^99^, ^100^, ^101^, ^102^, ^103^, ^104^, ^105^, ^106^ ^]^

## Conflict of Interests

The authors declare no conflict of interest.

## Supporting information



Supporting Information

## Data Availability

The data that support the findings of this study are available in the supplementary material of this article.
