# Supplementary material for: A Joyful Journey: Tungsten(VI) and Tungsten(V) Fluorides Meet N‐Heterocyclic Carbenes and Cyclic (Alkyl)(Amino)Carbenes
Source: Angew Chem Int Ed Engl. 2025 May 15;64(25):e202504498. doi: 10.1002/anie.202504498 (PMC12171349; doi:10.1002/anie.202504498)
Supplement: Supplementary file 1 — Supporting Information [file ANIE-64-e202504498-s001.docx]

**Table of Contents**

1. **Experimental Section**
2. **NMR spectra**
3. **Crystallographic Details**
4. **Additional Tables and Figures**
5. **Thermal analyses**
6. **Computational Details − Optimized Geometries**
7. **Experimental Section**

Caution! [WF_6_] is highly corrosive and toxic, undergoing rapid HF evolution upon exposure to moisture. Appropriate precautions must be implemented during its handling.

**General:** All reactions and subsequent manipulations involving air sensitive organometallic compounds were carried out under an argon atmosphere by using standard Schlenk techniques or in a Glovebox (Innovative Technology Inc. and MBraun Uni Lab).^[1]^ All reactions were performed in oven-dried glassware equipped with valves with PTFE stems (Rettberg, Göttingen and Young, London). Elemental analyses were performed in the microanalytical laboratory of the Institute of Inorganic Chemistry of the University Würzburg with an Elementar vario micro cube. High-resolution mass analysis (HRMS) was performed on a Thermo Scientific Exactive Plus mass spectrometer, equipped with an Orbitrap Mass Analyzer. Solution NMR spectra were recorded on a Bruker Avance 400 (^1^H, 400.1 MHz; ^13^C, 100.7 MHz; ^19^F, 376.8 MHz), a Bruker Avance 500 (^1^H, 500.1 MHz; ^13^C, 125.8 MHz; ^19^F, 470.5 MHz) and a Bruker Advance 600 (^1^H, 600.2 MHz; ^13^C, 150.9 MHz; ^19^F, 564.7 MHz) spectrometer using benzene-*d*_6_ or toluene-*d*_8_ as solvent. Solid-state RSHE/MAS (RSHE = rotor synchronized hahn echo) NMR spectra were recorded at 293 K with a Bruker Avance NEO 400 NMR spectrometer with bottom layer rotors of ZrO_2_ (outer diameter 4 mm with KelF rotor cap) containing approximately 100 *μ*L of sample (ca. 60–130 mg) spinning the rotor at 23 kHz. Assignment of the ^1^H NMR and ^13^C NMR data was supported by ^1^H, ^1^H and ^13^C{^1^H},^1^H correlation experiments. ^13^C NMR spectra were recorded broad-band proton-decoupled (^13^C{^1^H}) at 298 K if not otherwise noted. Chemical shifts are listed in parts per million (ppm), reported relative to TMS and were calibrated against residual solvent signals (*δ*(^1^H): C_6_D_5_*H*: 7.16; toluene-*d*_8_: 2.08, 6.97, 7.01, 7.09; ­*δ*(^13^C): *C*_6_D_6_: 128.06; toluene-*d*_8_: 20.43, 125.13, 127.96, 128.87, 137.48) and external CFCl_3_ (*δ*(^19^F): 0).^[2]^ If not otherwise noted ^19^F spectra were not proton decoupled. Coupling constants are quoted in Hertz. Infrared spectra were recorded on a Bruker Alpha FT-IR spectrometer by using an ATR unit. All IR spectra were measured in the region of 4000−400 cm^-1­^ with an apodized resolution of 2 cm^-1^ under an argon atmosphere on solid samples at room temperature and values are given in cm^-1^. Raman spectra were measured at room temperature with a MultiRAM FT‐Raman spectrometer using the 1064 nm excitation line of a Nd/YAG laser on solid samples contained in melting point capillaries in the region of 4000−400 cm­^-1^ with a resolution of 4 cm^−1^. All Raman spectra were recorded with low laser intensity (27−120 mW), as decomposition of the samples was observed at higher power levels. UV/Vis spectra were recorded on a JASCO V-670 spectrophotometer in a sealed cuvette in *n*-hexane. EPR measurements at X-band were carried out using a Bruker ELEXSYS E580 CW EPR spectrometer. The spectral simulations were performed using MATLAB 9.14.0.2206163 (R2023a) and the EasySpin 5.2.36 toolbox.^[3]^ Thermal analyses were performed with a DSC 204 F1 Phoenix (Netzsch) in the temperature range of 0 to 550 °C with a heating rate of 10 K∙min^-1^. All solvents were HPLC grade, further treated to remove traces of water using an Innovative Technology Inc. Pure-Solv Solvent Purification System or dried according to standard protocol^[4]^ and stored under argon atmosphere in flasks with valves with PTFE stems (Rettberg, Göttingen and Young, London). Benzene-*d*_6_ and toluene-*d*_8_ were purchased from Sigma-Aldrich and stored over molecular sieve. The carbenes I*i*Pr^Me^,^[5]^ IMes,^[6]^ IDipp,^[7]^ SIDipp,^[7]^ BI*i*Pr,^[8-10]^ and cAAC^Me[11, 12]^ were synthesized according to literature procedures. [WF_6_] (Solvay Fluor GmbH) was condensed from a gas bottle into a 250 mL glass bulb equipped with a PTFE valve (Rettberg, Göttingen) and stored at 4 °C. 1,4-bis(trimethylsilyl)-1,4-dihydropyrazine (TMS-py-TMS) was prepared according to literature procedure.^[13]^ 2,3,5,6-tetramethyl-1,4-bis(trimethylsilyl)-1,4-dihydropyrazine (TMS-py^Me^-TMS) was prepared according to a modified literature procedure by using sodium metal instead of potassium metal.^[14]^ All other starting materials were purchased from commercial sources and used without further purification.

**[(I*i*Pr^Me^)WF_6_] 1:** A solution of I*i*Pr^Me^ (100 mg, 555 *µ*mol, 1.0 eq) in 10 mL *n*-hexane was frozen in liquid nitrogen and [WF_6_] (555 *µ*mol, 1.0 eq) was condensed in at −196 °C. Upon warming the reaction mixture to ambient temperature, a red solid precipitated. After stirring the reaction mixture for 3 h at room temperature, all volatiles were removed under reduced pressure and the remaining solid was dried *in vacuo* yielding **1** (236 mg, 494 *µ*mol, 89%) as a red powder. **Elemental analysis** found (calcd.) for C_7_H_10_F_6_N_2_W [478.13 g/mol]: C 28.52 (27.63), H 4.30 (4.22), N 6.35 (5.86). **^1^H NMR** (400.1 MHz, C_6_D_6_, 298 K): *δ* [ppm] = 0.99 (d, 12 H, ^3^*J*_HH_ = 7.0 Hz, *i*Pr-C*H*_3_), 1.48 (s, 6 H, NCC*H*_3_CC*H*_3_N), 5.15 (sept, 2 H, ^3^*J*_HH_ = 7.0 Hz, *i*Pr-C*H*). **^13^C{^1^H} NMR** (125.8 MHz, C_6_D_6_, 298 K): *δ* [ppm] = 10.3 (NC*C*H_3_C*C*H_3_N), 21.1 (*i*Pr-*C*H_3_), 52.2 (sept*_br_*, 2 C, ^4^*J*_CF_ = 3.2 Hz, *i*Pr-*C*H), 125.8 (N*CC*N), 198.6 (sept*_br_*, 1 C, ^2^*J*_CF_ = 21.2 Hz, N*C*N). **^19^F NMR** (470.5 MHz, C_6_D_6_, 298 K): *δ* [ppm] = 151.5 (s, 6 F, ^1^*J*_WF_ = 35.8 Hz). **IR** (ATR [cm^-1^]): 2995 (vw), 2949 (vw), 2322 (vw), 1633 (vw), 1613 (vw), 1558 (vw), 1465 (vw), 1445 (vw), 1403 (w), 1389 (vw), 1377 (m), 1335 (vw), 1319 (vw), 1229 (vw), 1201 (w), 1171 (vw), 1145 (vw), 1112 (w), 1019 (vw), 977 (vw), 904 (vw), 751 (vw), 707 (vw), 685 (m), 609 (vs), 542 (vw), 526 (w), 460 (w), 414 (vw). **Raman** [cm^-1^]: 686.

**[(BI*i*Pr)WF_6_] 2:** A solution of BI*i*Pr (500 mg, 2.47 mmol, 1.0 eq) in 15 mL *n*-hexane was frozen in liquid nitrogen and [WF_6_] (2.47 mmol, 1.0 eq) was condensed in at −196 °C. Upon warming the reaction mixture to ambient temperature, a dark red solid precipitated. After stirring the reaction mixture overnight at room temperature, the solid turned yellow. All volatiles were removed under reduced pressure and the remaining solid was dried *in vacuo* yielding **2** (1.07 g, 2.14 mmol, 87%) as a yellow powder. **Elemental analysis** found (calcd.) for C_13_H_18_F_6_N_2_W [500.13 g/mol]: C 32.21 (31.22), H 3.81 (3.63), N 5.92 (5.60).  **^1^H NMR** (600.2 MHz, toluene-*d*_8_, 298 K): *δ* [ppm] = 1.14 (d, 12 H, ^3^J*_HH_* = 7.0 Hz, *i*Pr-C*H*_3_), 5.27 (sept, 2 H, ^3^*J*_HH_ = 7.0 Hz, *i*Pr-C*H*), 6.89−6.93 (m, 2 H, aryl-C*H*), 7.14−7.18 (m, 2 H, aryl-C*H*). **^13^C{^1^H} NMR** (150.9 MHz, toluene-*d*_8_, 298 K): *δ* [ppm] = 20.4 (*i*Pr-*C*H_3_), 53.1 (*i*Pr-*C*H), 115.7 (aryl-*C*H), 124.1 (aryl-*C*H), 132.0 (aryl-N*CC*N), 201.9 (N*C*N, assignment *via* 2D-HMBC spectrum). **^19^F NMR** (376.8 MHz, C_6_D_6_, 298 K): *δ* [ppm] = 152.0 (s, 6 F, ^1^*J*_WF_ = 35.2 Hz). **IR** (ATR [cm^-1^]): 2991 (w), 2948 (vw), 2161 (vw), 1602 (vw), 1560 (vw), 1478 (m), 1420 (w), 1392 (s), 1375 (m), 1299 (m), 1246 (vw), 1211 (vw), 1171 (vw), 1147 (m), 1094 (s), 1069 (vw), 1027 (vw), 978 (vw), 889 (vw), 852 (vw), 801 (m), 752 (s), 689 (s), 658 (w), 610 (vs), 542 (m), 529 (m), 511 (m), 429 (m). **Raman**[cm^-1^]: 690.

Yellow crystals of [(BI*i*Pr)WF­_6_] **2** suitable for single crystal X-ray diffraction were obtained by slow diffusion of *n*-hexane into a saturated solution of **5** in benzene.

**[(IMes)WF_6_] 3:** A solution of IMes (50 mg, 164 *µ*mol, 1.0 eq) in 10 mL *n*-hexane was frozen in liquid nitrogen and [WF_6_] (164 *µ*mol, 1.0 eq) was condensed in at −196 °C. Upon warming the reaction mixture to ambient temperature, a brown solid precipitated. After stirring the reaction mixture for 3 h at room temperature, the solid turned yellow. All volatiles were removed under reduced pressure and the remaining solid was dried *in vacuo* yielding **3** (80.2 mg, 134 *µ*mol, 82%) as a yellow powder. **Elemental analysis** found (calcd.) for C_21_H_24_F_6_N_2_W [602.27 g/mol]: C 41.32 (41.88), H 4.29 (4.02), N 4.38 (4.65). **^1^H NMR** (400.1 MHz, C_6_D_6_, 298 K): *δ* [ppm] = 2.02 (s, 12 H, C*H*_3-_*_ortho_*), 2.03 (s, 6 H, C*H*_3-_*_para_*), 5.81 (s, 2 H, NC*H*C*H*N), 6.63 (s, 4 H, aryl-C*H_meta_*). **^13^C{^1^H} NMR** (100.7 MHz, C_6_D_6_, 298 K): *δ* [ppm] = 17.6 (*C*H_3-_*_ortho_*), 21.0 (*C*H_3-_*_para_*), 122.7 (N*C*H*C*HN), 129.1 (aryl-*C*H*_meta_*), 134.9 (aryl-*C_ipso_*), 135.4 (aryl-*C_ortho_*), 139.6 (aryl-*C_para_*), 201.4 (sept*_br_*, 1 C, ^2^*J*_CF_ = 22.1 Hz, N*C*N). **^19^F NMR** (376.8 MHz, C_6_D_6_, 298 K): *δ* [ppm] = 149.7 (s, 6 F, ^1^*J*_WF_ = 27.1 Hz). **IR** (ATR [cm^-1^]): 3174 (vw), 3143 (vw), 2921 (vw), 2859 (vw), 1607 (vw), 1482 (w), 1460 (vw), 1410 (vw), 1382 (vw), 1289 (vw), 1225 (w), 1121(vw), 1085 (vw), 1036 (vw), 1115 (vw), 929 (vw), 855 (w), 759 (vw), 718 (w), 708 (vw), 668 (s), 615 (vs), 573 (s), 526 (vw), 456 (vw). **Raman** [cm^-1^]: 708.

Yellow crystals of [(IMes)WF­_6_] **3** suitable for single crystal X-ray diffraction were obtained by slow diffusion of *n*-hexane into a saturated solution of **3** in benzene.

**[(IDipp)WF_6_] 4:** A solution of IDipp (500 mg, 1.29 mmol, 1.0 eq) in 15 mL *n*-hexane was frozen in liquid nitrogen and [WF_6_] (1.29 mmol, 1.0 eq) was condensed in at −196 °C. Upon warming the reaction mixture to ambient temperature, a yellow solid precipitated. After stirring the reaction mixture for 3 d at room temperature, all volatiles were removed under reduced pressure and the remaining solid was dried *in vacuo* yielding **4** (698 mg, 1.02 mmol, 79%) as a yellow powder. **Elemental analysis** found (calcd.) for C_27_H_36_F_6_N_2_W [686.43 g/mol]: C 47.09 (47.24), H 5.22 (5.29), N 4.18 (4.08). **^1^H NMR** (600.2 MHz, toluene-*d*_8_, 298 K): *δ* [ppm] = 0.97 (d, 12 H, ^3^*J*_HH_ = 6.8 Hz, *i*Pr-C*H_3_*), 1.28 (d, 12 H, ^3^*J*_HH_ = 6.8 Hz, *i*Pr-C*H_3_*), 2.76 (sept, 2 H, ^3^*J*_HH_ = 6.8 Hz, *i*Pr-C*H*), 6.33 (s, 2 H, NC*H*C*H*N), 6.97 (d, 4 H, aryl-C*H_meta_*, overlap with solvent), 7.12 (t, 2 H, ^3^*J*_HH_ = 7.7 Hz, aryl-C*H_para_*). **^13^C{^1^H} NMR** (150.9 MHz, toluene-*d*_8_, 298 K): *δ* [ppm] = 22.6 (*i*Pr-*C*H_3_), 26.0 (*i*Pr-*C*H_3_), 29.3 (*i*Pr-*C*H), 123.8 (N*C*H*C*HN), 124.0 (aryl-*C*H*_meta_*), 130.8 (aryl-*C*H*_para_*), 134.6 (aryl-*C_ipso_*), 145.9 (aryl-*C_ortho_*), 202.0 (sept*_br_*, 1 C, ^2^*J*_CF_ = 22.2 Hz, N*C*N). **^19^F NMR** (470.6 MHz, toluene-*d*_8_, 298 K): *δ* [ppm] = 151.1 (s, 6 F, ^1^*J*_WF_ = 27.1 Hz). **IR** (ATR [cm^-1^]): 3177 (vw), 3148 (vw), 2965 (w), 2932 (vw), 2870 (vw), 1596 (vw), 1564 (vw), 1476 (vw), 1466 (w), 1455 (w), 1409 (w), 1386 (w), 1365 (w), 1352 (vw), 1329 (w), 1307 (vw), 1288 (vw), 1272 (vw), 1256 (vw), 1207 (vw), 1183 (vw), 1164 (vw), 1144 (vw), 1114 (w), 1059 (w), 1042 (vw), 1019 (vw), 1004 (vw), 983 (vw), 971 (vw), 948 (w), 935 (w), 805 (m), 760 (m), 724 (m), 708 (m), 670 (s), 613 (vs), 570 (s), 549 (m), 520 (w), 461 (w), 438 (w). **Raman** [cm^-1^]: 710.

Yellow crystals of [(IDipp)WF_6_] **4** suitable for single crystal X-ray diffraction were obtained by slow diffusion of *n*-hexane into a saturated solution of **4** in toluene. Due to poor data to parameter ratio the crystal structure of **4** serves merely as evidence of structure.

**[(SIDipp)WF_6_] 5:** A solution of SIDipp (100 mg, 256 *μ*mol, 1.0 eq) in 10 mL *n*-hexane was frozen in liquid nitrogen and [WF_6_] (256 *μ*mol, 1.0 eq) was condensed in at −196 °C. Upon warming the reaction mixture to ambient temperature, an orange solid precipitated. After stirring the reaction mixture overnight at room temperature, all volatiles were removed under reduced pressure and the remaining solid was dried *in vacuo* yielding **5** (143 mg, 208 *μ*mol, 83%) as an orange powder. **Elemental analysis** found (calcd.) for C_27_H_38_F_6_N_2_W [688.45 g/mol]: C 47.08 (47.11), H 5.70 (5.56), N 4.06 (4.07).  **^1^H NMR** (400.1 MHz, C_6_D_6_, 298 K): *δ* [ppm] = 1.10 (d, 12 H, ^3^*J*_HH_ = 6.8 Hz, *i*Pr-C*H_3_*), 1.40 (d, 12 H, ^3^*J*_HH_ = 6.8 Hz, *i*Pr-C*H_3_*), 3.37 (sept, 2 H, ^3^*J*_HH_ = 6.8 Hz, *i*Pr-C*H*), 3.45 (s, 4 H, NC*H*_2_C*H*_2_N), 6.98 (d, 4 H, ^3^*J*_HH_ = 7.8 Hz, aryl-C*H_meta_*,), 7.11 (t, 2 H, ^3^*J*_HH_ = 7.8 Hz, aryl-C*H_para_*). **^13^C{^1^H} NMR** (100.7 MHz, C_6_D_6_, 298 K): *δ* [ppm] = 23.7 (*i*Pr-*C*H_3_), 26.2 (*i*Pr-*C*H_3_), 29.1 (*i*Pr-*C*H), 54.6 (N*C*H_2_*C*H_2_N), 124.8 (aryl-*C*H*_meta_*), 130.2 (aryl-*C*H*_para_*), 134.5 (aryl-*C_ipso_*), 146.6 (aryl-*C_ortho_*), 224.7 (sept*_br_*, 1 C, ^2^*J*_CF_ = 19.1 Hz, N*C*N N*C*N) **^19^F NMR** (376.8 MHz, C_6_D_6_, 298 K): *δ* [ppm] = 145.6 (s, 6 F, ^1^*J*_WF_ = 23.1 Hz). **IR** (ATR [cm^-1^]): 2963 (m), 2929 (w), 2870 (w), 1630 (m), 1588 (vw), 1481 (m), 1456 (m), 1387 (w), 1366 (w), 1323 (w), 1301 (vw), 1270 (m), 1183 (vw), 1104 (vw), 1056 (w), 978 (vw), 934 (w), 912 (w), 807 (m), 761 (m), 710 (m), 674 (vs), 608 (vs), 573 (vs), 550 (m), 517 (m), 458 (m), 439 (m). **Raman** [cm^-1^]: 711.

**[(****cAAC^Me^)WF_6_] 6:** A solution of cAAC^Me^ (500 mg, 1.75 mmol, 1.0 eq) in 10 mL *n*-hexane was frozen in liquid nitrogen and [WF_6_] (1.75 *µ*mo, 1.0 eq) was condensed in at −196 °C. Upon warming the reaction mixture to ambient temperature, a yellow solid precipitated. After stirring the reaction mixture for 2 h at room temperature, all volatiles were removed under reduced pressure and the remaining solid was dried *in vacuo* yielding **6** (941 mg, 1.61 mmol, 92%) as a yellow powder. **Elemental analysis** found (calcd.) for C_20_H_31_F_6_NW [583.31 g/mol]: C 40.92 (41.18), H 5.19 (5.36), N 2.80 (2.40). **^1^H NMR** (400.1 MHz, C_6_D_6_, 298 K): *δ* [ppm] = 0.83 (s, 6 H, W-C-C(C*H*_3_)_2_/N-C(C*H*_3_)_2_), 1.08 (d, 6 H, ^3^*J*_HH_ = 6.5 Hz, *i*Pr-C*H*_3_), 1.30 (d, 6 H, ^3^*J*_HH_ = 6.5 Hz, *i*Pr-C*H*_3_), 1.46 (s, 6 H, W-C-C(C*H*_3_)_2_/N-C(C*H*_3_)_2_), 1.47 (s, 2 H, C*H*_2_), 2.78 (sept, 2 H, ^3^*J*_HH_ = 6.5 Hz, *i*Pr-C*H*), 6.89 (d, 2 H, ^3^*J*_HH_ = 8.0 Hz, aryl-C*H_meta_*), 7.00 (t, 1 H, ^3^*J*_HH_ = 8.0 Hz, aryl-C*H_para_*). **^13^C{^1^H} NMR** (125.8 MHz, C_6_D_6_, 298 K): *δ* [ppm] = 24.6 (*i*Pr-*C*H_3_), 26.8 (*i*Pr-*C*H_3_), 28.78 (W-C-C(*C*H_3_)_2_/N-C(*C*H_3_)_2_), 28.97 (W-C-C(*C*H_3_)_2_/N-C(*C*H_3_)_2_), 29.6 (*^i^*Pr-*C*H), 52.9 (*C*H_2_), 57.1 (W-C-*C*(CH_3_)_2_), 82.4 (N-*C*(CH_3_)_2_), 125.4 (aryl-*C_meta_*), 130.0 (aryl-*C_para_*), 133.4 (aryl-*C_ipso_*), 145.2 (aryl-*C_ortho_*), 266.9 (sept*_br_*, 1 C, ^2^*J*_CF_ = 14.5 Hz). **^19^F NMR** (376.8 MHz, C_6_D_6_, 298 K): *δ* [ppm] = 140.6 ppm (s*_br_*, 6 F, ^1^*J*_WF_ coupling constant could not be determined). **IR** (ATR [cm^-1^]): 3008 (vw), 2975 (w), 2949 (vw), 2934 (vw), 2873 (vw), 1645 (vw), 1589 (vw), 1539 (w), 1465 (m), 1390 (vw), 1376 (w), 1343 (vw), 1318 (vw), 1264 (vw), 1204 (vw), 1191 (w), 1162 (vw), 1128 (w), 1108 (vw), 1093 (vw), 1053 (vw), 1013 (vw), 983 (vw), 953 (vw), 932 (vw), 913 (vw), 887 (vw), 876 (vw), 810 (m), 778 (m), 724 (m), 704 (m), 692 (s), 666 (s), 624 (s), 611 (vs), 599 (vs), 562 (vs), 496 (vw), 449 (vw), 433 (vw), 412 (vw). **Raman** [cm^-1^]: 705.

Yellow crystals of [(cAAC^Me^)WF_6_] **6** suitable for single crystal X-ray diffraction were obtained by slow diffusion of *n*-hexane into a saturated solution of **6** in benzene.

**[(I*i*Pr^Me^)WF_5_] 7:** [(I*i*Pr^Me^)WF_6_] **1** (100 mg, 209 *µ*mol, 1.0 eq) and TMS-py^Me^-TMS (29.6 mg, 104 *µ*mol, 0.5 eq) were dissolved in 10 mL toluene whereby a colour change from dark orange to brown was observed. After stirring for 6 h at room temperature all volatiles were removed under reduced pressure and the remaining solid was suspended in 7 mL *n*-hexane. After filtration the solid was washed with 7 mL of *n*-hexane and dried *in vacuo* yielding **7** (51.1 mg, 111 *µ*mol, 53%) as a light-yellow powder. **^1^H NMR** (400.1 MHz, C_6_D_6_, 298 K): *δ* [ppm] = 2.43 (s, 12 H, *i*Pr-C*H*_3_), 3.16 (s, 6 H, C*H*_3_CCC*H*_3_), 10.99 (s*_br_,* 2 H, *i*Pr-C*H*). **^13^C{^1^H} NMR** (100.7 MHz, C_6_D_6_, 298 K): *δ* [ppm] = 9.8 (NC*C*H_3_C*C*H_3_N), 34.3 (*i*Pr-*C*H_3_), 48.4 (*i*Pr-*C*H, assignment *via* 2D-HMBC spectrum), 95.9 (N*CC*N, assignment *via* 2D-HMBC spectrum). The carbene carbon atom was not detected. **^19^F NMR** (376.8 MHz, C_6_D_6_, 298 K): no signal observed. **HRMS** (ASAP) *m*/*z* found (calcd.) for C_11_H_20_F_5_N_2_W: 459.1050 (459.1050). **Elemental analysis** found (calcd.) for C_11_H_20_F_5_N_2_W [459.13 g/mol]: C 29.41 (28.78), H 4.52 (4.39), N 6.10 (6.10). **IR** (ATR [cm^-1^]): 3162 (w), 2991 (w), 2941 (w), 1615 (w), 1558 (m), 1465 (m), 1370 (m), 1269 (w), 1229 (m), 1196 (m), 1142 (w), 1115 (w), 1079 (vw), 976 (s), 902 (w), 769 (w), 750 (w), 734 (w), 675 (s), 602 (vs), 544 (m), 518 (s), 435 (w). **Raman** [cm^-1^]: 673.

Yellow crystals of [(I*i*Pr^Me^)WF­_5_] **7** suitable for single crystal X-ray diffraction were obtained by slow diffusion of *n*-hexane into a saturated solution of **7** in THF.

**[(BI*i*Pr)WF_5_] 8:** [(BI*i*Pr)WF**­_6_**­] (100 mg, 200 *µ*mol, 1.0 eq) **2** and TMS-py^Me^-TMS (28.3 mg, 100 *µ*mol, 0.5 eq) were dissolved in 10 mL toluene whereby a colour change from yellow to orange was observed. After stirring for 1 h at room temperature all volatiles were removed under reduced pressure and the remaining solid was suspended in 7 mL *n*-hexane. After filtration the solid was washed with 2 mL of *n*-hexane and dried *in vacuo* yielding **8** (65.2 mg, 111 *µ*mol, 68%) as an off-white powder. **^1^H NMR** (600.2 MHz, C_6_D_6_, 298 K): *δ* [ppm] = 1.95 (s, 12 H, *i*Pr-C*H*_3_), 5.52−5.57 (m, 2 H, aryl-C*H*), 5.74−5.77 (m, 2 H, aryl-C*H*), 10.19 (s*_br_*, 2 H, *i*Pr-C*H*).**^13^C{^1^H} NMR** (150.9 MHz, C_6_D_6_, 298 K): *δ* [ppm] = 35.2 (*i*Pr-*C*H_3_), 39.2 (*i*Pr-*C*H), ), 91.2 (aryl-N*CC*N), 114.9 (aryl-*C*H), 127.2 (aryl-*C*H). The carbenen carbon atom was not detected. **^19^F NMR** (564.7 MHz, C_6_D_6_, 298 K): no signal observed. **HRMS** (ASAP) *m*/*z* found (calcd.) for C_13_H_18_F_5_N_2_W: 481.0907 (481.0905). **Elemental analysis** found (calcd.) for C_13_H_18_F_5_N_2_W [481.13 g/mol]: C 34.58 (32.45), H 4.08 (3.77), N 6.16 (5.82). **IR** (ATR [cm^-1^]): 3151 (vw), 2981 (m), 2942 (m), 1601 (m), 1559 (m), 1474 (s), 1440 (m), 1397 (m), 1368 (s), 1319 (w), 1297 (m), 1245 (m), 1211 (m), 1171 (vw),1143 (s), 1095 (s), 1071 (m), 1024 (w), 978 (m), 938 (w), 888 (m), 802 (m), 785 (vw), 742 (vs), 716 (vw), 685 (s), 611 (vs), 579 (vs), 552 (vs), 512 (s), 550 (m), 421 (s). **Raman** [cm^-1^]: 687.

Yellow crystals of [(BI*i*Pr)WF­_5_] **8** suitable for single crystal X-ray diffraction were obtained by slow diffusion of *n*-hexane into a saturated solution of **8** in benzene.

**[(IMes)WF_5_] 9:** A solution of [(IMes)WF_6_] **3** (100 mg, 166 *µ*mol, 1.0 eq) and TMS-py^Me^-TMS (23.5 mg, 83.0 *µ*mol, 0.5 eq) were dissolved in 10 mL toluene whereby a colour change from orange to dark was observed. After stirring overnight at room temperature all volatiles were removed under reduced pressure and the remaining solid was suspended in 10 mL *n*-hexane. After filtration the solid was washed three times with 5 mL of *n*-hexane and dried *in vacuo* yielding **9** (76.0 mg, 130 *µ*mol, 78%) as an off-white powder. **^1^H NMR** (400.1 MHz, C_6_D_6_, 298 K): *δ* [ppm] = 2.07 (s, 12 H, C*H_3-ortho_*), 2.38 (s, 6 H, C*H_3-para_*), 4.12 (sept*_br_*, 2 H, *i*Pr-C*H*), 7.43 (s, 4 H, aryl-C*H_meta_*). **^13^C{^1^H} NMR** (150.9 MHz, C_6_D_6_, 298 K): *δ* [ppm] = 14.6 (*C*H_3-_*_ortho_*), 21.1 (*C*H_3-_*_para_*), 92.1 (N*C*H*C*HN, assignment *via* 2D-HMBC spectrum), 112.5 (*C_ortho_*_,_ assignment *via* 2D-HMBC spectrum), 130.9 (aryl-*C*H*_meta_*), 140.7 (aryl-*C_para_*), 152.9 (aryl-*C_ipso_*). The carbene carbon atom was not detected. **^19^F NMR** (564.7 MHz, C_6_D_6_, 298 K): no signal observed. **HRMS**(ASAP) *m*/*z* found (calcd.) for C_21_H_24_F_5_N_2_W: 583.1359 (583.1363). **Elemental analysis** found (calcd.) for C_21_H_24_F_5_N_2_W [583.27 g/mol]: C 44.44 (43.24), H 4.42 (4.15), N 4.85 (4.80). **IR** (ATR [cm^-1^]): 3148 (vw), 2921 (w), 2860 (w), 2870 (w), 1607 (m), 1543 (m), 1483 (m), 1450 (m), 1406 (w), 1381 (w), 1311 (vw), 1287 (vw), 1226 (s), 1162 (vw),1125 (vw), 1103 (vw), 1062 (w), 1035 (m), 984 (m), 930 (m), 866 (m), 851 (s), 770 (s), 734 (vw), 711 (m), 692 (m), 676 (m), 597 (vs), 572 (s), 562 (m), 550 (m), 485 (m), 455 (m). **Raman** [cm^-1^]: 691.

Colourless crystals of [(IMes)WF_5_] **9** suitable for single crystal X-ray diffraction were obtained by slow diffusion of *n*-hexane into a saturated solution of **9** in 1,2-difluorbenzene.

**[(IDipp)WF_5_] 10:** A solution of [(IDipp)WF_6_] **4** (300 mg, 437 *µ*mol, 1.0 eq) and TMS-py^Me^-TMS (61.8 mg, 219 *µ*mol, 0.5 eq) were dissolved in 15 mL toluene whereby a colour change from orange to yellow was observed. After stirring for 3 h at room temperature all volatiles were removed under reduced pressure and the remaining solid was suspended in 10 mL
*n*-hexane. After filtration the solid was washed two times with 7 mL of *n*-hexane and dried *in vacuo* yielding **10** (235 mg, 352 *µ*mol, 81%) as an off-white powder. **^1^H NMR** (600.2 MHz, C_6_D_6_, 298 K): *δ* [ppm] = 0.82 (d, 12 H, ^3^*J*_HH_ = 5.6 Hz, *i*Pr-C*H_3_*), 1.70 (d, 12 H, ^3^*J*_HH_ = 5.6 Hz, *i*Pr-C*H_3_*), 2.66 (sept*_br_*, 2 H, *i*Pr-C*H*), 4.74 (s, 2 H, NC*H*C*H*N), 7.32 (t, 2 H, ^3^*J*_HH_ = 7.8 Hz, aryl-C*H_para_*), 7.69 (d, 4 H, ^3^*J*_HH_ = 7.8 Hz, aryl-C*H_meta_*). **^13^C{^1^H} NMR** (150.9 MHz, C_6_D_6_, 298 K): *δ* [ppm] = 22.3 (*i*Pr-*C*H_3_), 26.7 (*i*Pr-*C*H_3_), 27.2 (*i*Pr-*C*H), 112.5 (N*C*H*C*HN), 125.6 (aryl-*C*H*_meta_*), 131.6 (aryl-*C*H*_para_*), 142.5 (aryl-*C_ipso_*), 161.7 (aryl-*C_ortho_*). The carbene carbon atom was not detected. **^19^F NMR** (564.7 MHz, C_6_D_6_, 298 K): no signal observed. **HRMS** (ASAP) *m*/*z* found (calcd.) for C_27_H_36_F_5_N_2_W: 667.2303 (667.2302). **Elemental analysis** found (calcd.) for C_27_H_36_F_5_N_2_W [667.43 g/mol]: C 48.46 (48.59), H 5.57 (5.44), N 4.50 (4.20). **IR** (ATR [cm^-1^]): 2966 (m), 2931 (m), 2873 (w), 1547 (w), 1465 (m), 1447 (m), 1405 (m), 1386 (w), 1364 (w), 1351 (vw), 1327 (w), 1295 (vw), 1256 (w), 1206 (w), 1183 (vw), 1117 (vw), 1061 (w), 980 (vw), 946 (vw), 937 (w), 886 (vw), 803 (m), 768 (m), 758 (s), 710 (w), 692 (w), 678 (w), 621 (vs), 589 (s), 576 (vs), 482 (vw), 455 (w), 419 (vw). **Raman** [cm^-1^]: 693.

Colourless crystals of [(IDipp)WF­_5_] **10** suitable for single crystal X-ray diffraction were obtained by slow evaporation of a saturated solution of **10** in benzene.

**[(SIDipp)WF_5_] 11:** A solution of [(SIDipp)WF_6_] **5** (50 mg, 72.6 *µ*mol, 1.0 eq) and TMS-py^Me^-TMS (10.3 mg, 72.6 *µ*mol, 0.5 eq) were dissolved in 10 mL toluene whereby a colour change from orange to yellow was observed. After stirring for 12 h at room temperature all volatiles were removed under reduced pressure and the remaining solid was suspended in 10 mL
*n*-hexane. After filtration the solid was washed two times with 5 mL of *n*-hexane and dried *in vacuo* yielding **11** (23.0 mg, 34.4 *µ*mol, 47%) as an off-white powder. **^1^H NMR** (600.2 MHz, C_6_D_6_, 298 K): *δ* [ppm] = 1.03 (d, 12 H, ^3^*J*_HH_ = 6.5 Hz, *i*Pr-C*H_3_*), 1.39 (d, 12 H, ^3^*J*_HH_ = 6.5 Hz, *i*Pr-C*H_3_*), 3.12 (sept*_br_*, 2 H, *i*Pr-C*H*), 4.68 (s, 2 H, NC*H*_2_C*H*_2_N), 6.88 (t, 2 H, ^3^*J*_HH_ = 7.9 Hz, aryl-C*H_para_*), 7.12 (d, 4 H, ^3^*J*_HH_ = 7.9 Hz, aryl-C*H_meta_*). **^13^C{^1^H} NMR** (150.9 MHz, C_6_D_6_, 298 K): *δ* [ppm] = 24.4 (*i*Pr-*C*H_3_), 25.3 (*i*Pr-*C*H_3_), 29.0 (*i*Pr-*C*H), 57.8 (N*C*H_2_*C*H_2_N, assignment *via* 2D-HMBC spectrum), 124.9 (aryl-*C*H*_meta_*), 128.6 (aryl-C*_ipso_*), 131.3 (aryl-*C*H*_para_*, assignment *via* 2D-HMBC spectrum), 147.3 (aryl-*C_ortho_*). The carbene carbon atom were not detected. **^19^F NMR** (564.7 MHz, C_6_D_6_, 298 K): no signal observed. **HRMS**(ASAP) *m*/*z* found (calcd.) for C_27_H_38_F_5_N_2_W: 669.2457 (669.2459). **Elemental analysis** found (calcd.) for C_27_H_38_F_5_N_2_W [663.45 g/mol]: C 48.59 (48.44), H 5.88 (5.72), N 4.03 (4.18). **IR** (ATR [cm^-1^]): 3067 (vw), 2963 (m), 2929 (w), 2870 (w), 1630 (s), 1587 (w), 1503 (vw), 1449 (w), 1389 (w), 1368 (w), 1325 (w), 1258 (m), 1191 (w), 1149 (vw),1101 (w), 1058 (w), 1017 (vw), 977 (m), 935 (w), 883 (w), **807** (m), **758** (m), 709 (vw), **676** (m), **597** (vs), 541 (m), 508 (m), 481 (m), 438 (m). **Raman** [cm^-1^]: 683.

**[(cAAC^Me^)WF_5_]_2_ 12:** [(cAAC^Me^)WF**­_6_**­] (100 mg, 171 *µ*mol, 1.0 eq) **6** was dissolved in 5 mL toluene and TMS-py-TMS (19.4 mg, 85.7 *µ*mol, 0.5 eq) was added whereby a colour change from orange to deep dark purple was observed. After stirring for 15 min at room temperature, all volatiles were removed under reduced pressure and the remaining solid was dissolved in 60 mL of *n*-hexane and stored at −30 °C overnight. The precipitated solid was collected by filtration at low temperatures and dried *in vacuo* yielding **12** (59.4 mg, 52.3 *µ*mol, 31%) as a dark purple powder. **EPR** (benzene, 298 K): *g* = 1.821. **Magnetic moment** (Evans’ method, C_6_D_6_): *μ*_eff_ = 1.57. **Elemental analysis** found (calcd.) for C_40_H_62_F_10_N_2_W_2_: [1128.61 g/mol]: C 43.54 (42.57), H 5.57 (5.54), N 4.06 (2.48). **IR** (ATR [cm^-1^]): 2968 (m), 2932 (w), 2869 (w), 1533 (w), 1468 (m), 1458 (m), 1419 (w), 1388 (w), 1370 (w), 1326 (vw), 1261 (vw), 1196 (w), 1183 (vw), 1163 (vw), 1128 (m), 1108 (w), 1093 (vw), 1054 (m), 971 (vw), 933 (vw), 878 (vw), 809 (s), **777** (s), 712 (m), 680 (w), 671 (w), **614** (vs), **590** (vs), **564** (vs), 486 (m), 447 (m), 415 (w). **Raman** [cm^-1^]: 726.

Red crystals of [(cAAC^Me^)WF_5_]_2_ **12** suitable for single crystal X-ray diffraction were obtained by slow evaporation of a saturated solution of **12** in benzene.

**[(cAAC^Me^)_2_WF_5_] 13:** [(cAAC^Me^)WF_5_]_2_(79. mg, 70.1 *µ*mol, 1.0 eq) **12** and cAAC^Me^ (40.0 mg, 140.2 *µ*mol, 2.0 eq) were dissolved in 7 mL benzene whereby a colour change from deep dark purple to deep dark red was observed. After stirring for 3 h at room temperature all volatiles were removed under reduced pressure and the remaining solid was dried *in vacuo* yielding **13** (65.8 mg, 77.4 *µ*mol, 55%) as a dark blue powder. **EPR** (benzene, 298 K): *g* = 1.814. **Magnetic moment** (Evans’ method, C_6_D_6_): *μ*_eff_ = 1.84 *μ*_B_. **Elemental analysis** found (calcd.) for C_40_H_62_F_5_N_2_W: [849.78 g/mol]: C 57.36 (56.54), H 7.45 (7.35), N 4.08 (3.30). **IR** (ATR [cm^-1^]): 2962 (m),2927 (m), 2868 (w), 1688 (vw), 1645 (vw), 1589 (vw), 1505 (w), 1466 (m), 1416 (vw), 1386 (w), 1367 (w), 1345 (vw), 1323 (vw), 1260 (s), 1195 (w), 1127 (m), 1091 (s), 1051 (s), 1017 (vs), 959 (w), 939 (w), 876 (w), 854 (w), 798 (vs), 772 (s), 704 (m), 677 (w), 663 (w), 598 (m), 573 (vs), 556 (s), 498 (m), 461 (m). **UV/Vis** (*n*-hexane, [nm]): 220, 260, 438.

Red crystals of [(cAAC^Me^)_2_WF_5_] **13** suitable for single crystal X-ray diffraction were obtained by slow evaporation of a solution of **13** in benzene.

1. **NMR spectra**

**
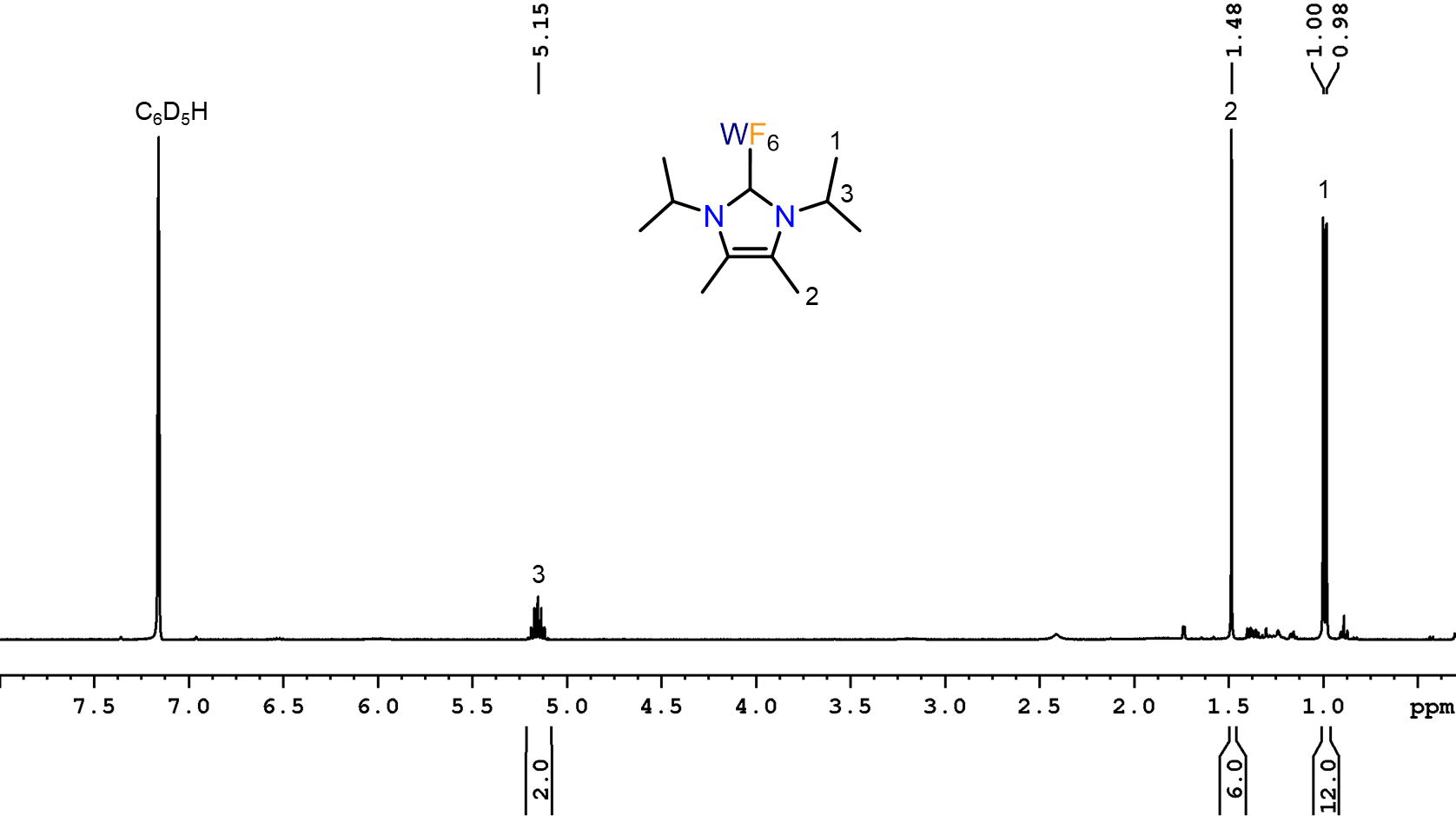
**

**Figure S1.** ^1^H NMR spectrum (400.1 MHz) of [(IiPr^Me^)WF_6_] **1** recorded in C_6_D_6_.


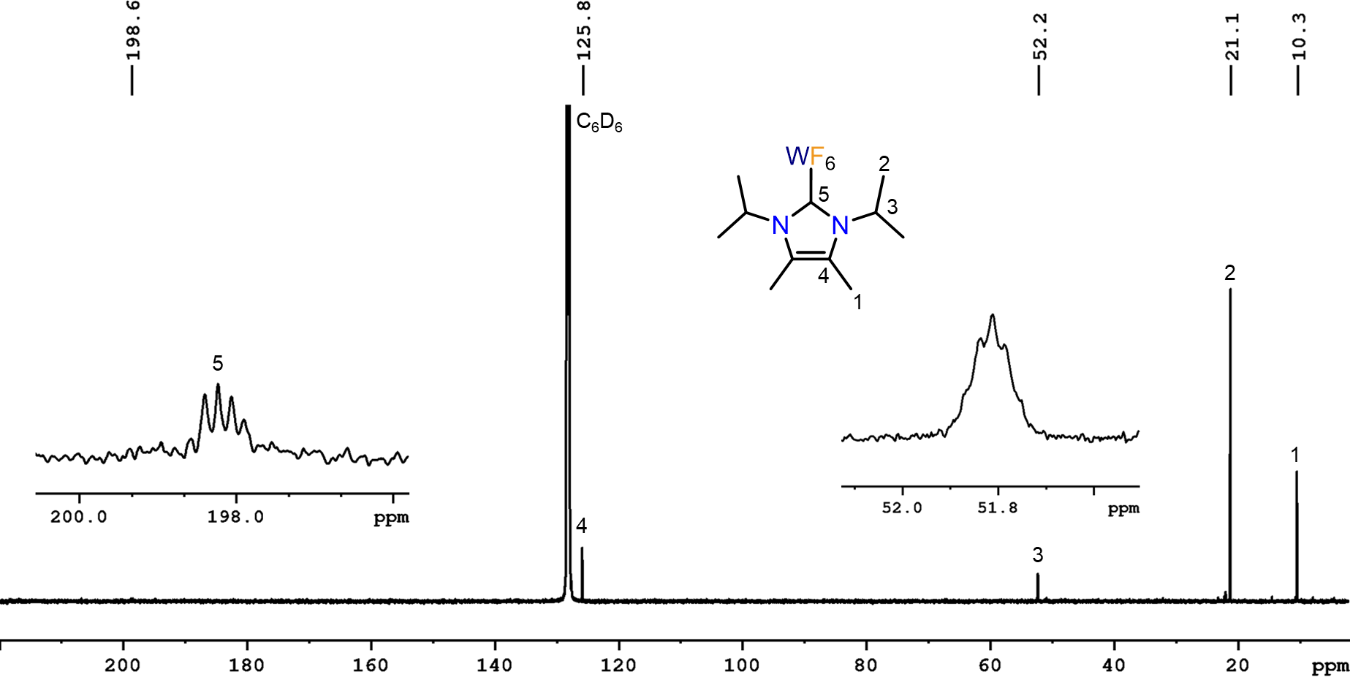


**Figure S2**. ^13^C{^1^H} NMR spectrum (125.8 MHz) of [(IiPr^Me^)WF_6_] **1** recorded in C_6_D_6_.


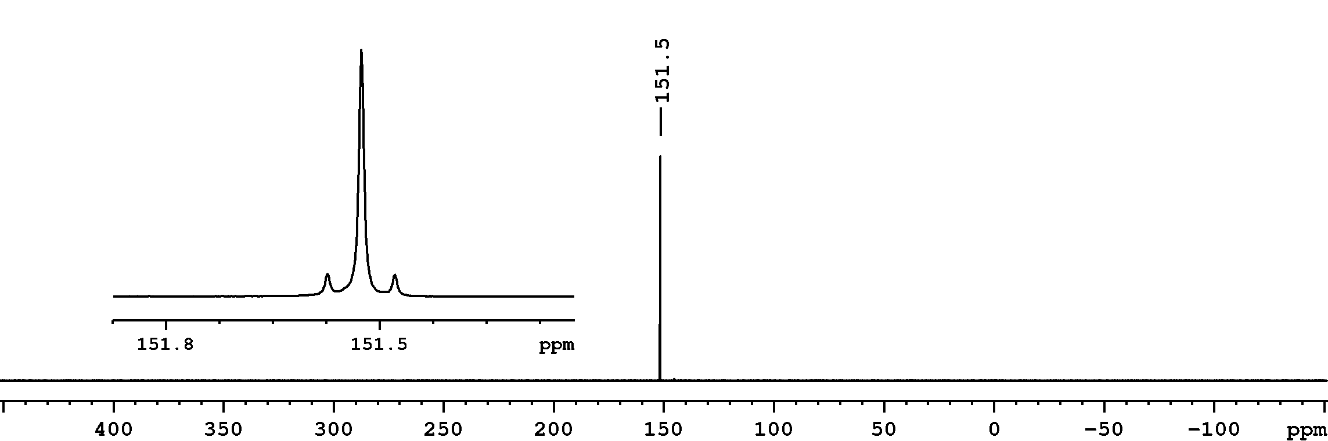


**Figure** **S3.** ^19^F{^1^H} NMR spectrum (470.5 MHz) of [(IiPr^Me^)WF_6_] **1** recorded in C_6_D_6_.


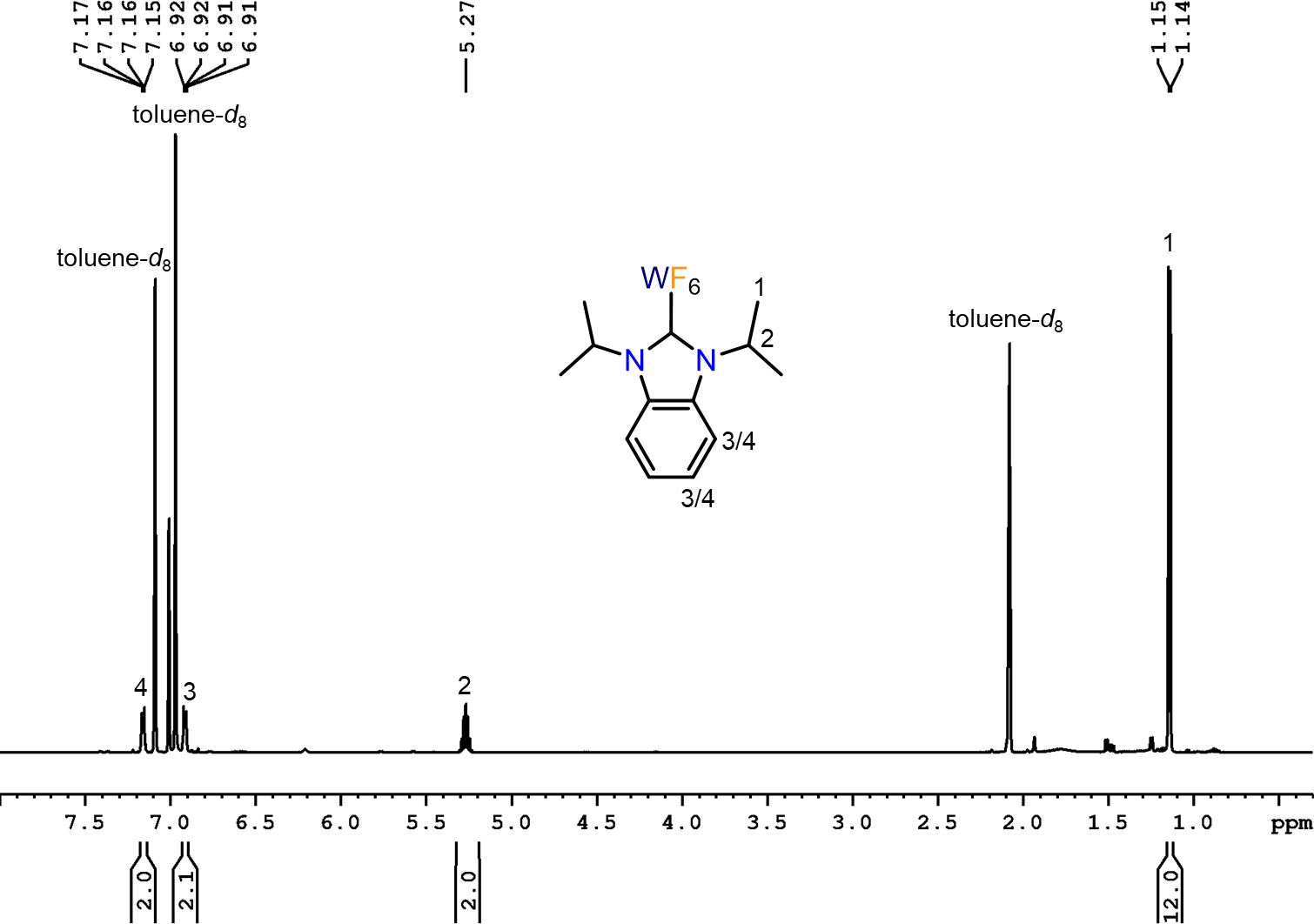


**Figure** **S4.** ^1^H NMR spectrum (600.2 MHz) of [(BI*i*Pr)WF_6_] **2** recorded in toluene-*d*_8_.


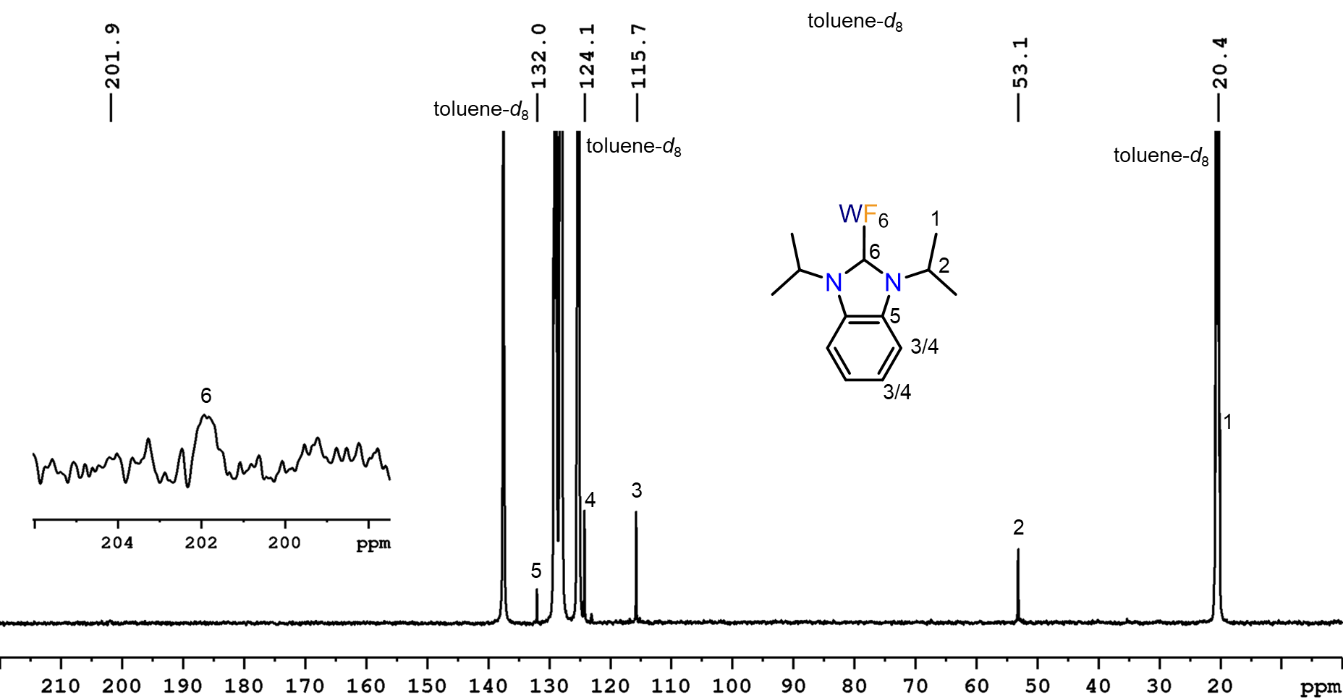


**Figure S5**. ^13^C{^1^H} NMR spectrum (150.9 MHz) of [(BI*i*Pr)WF_6_] **2** recorded in toluene-*d*_8_.


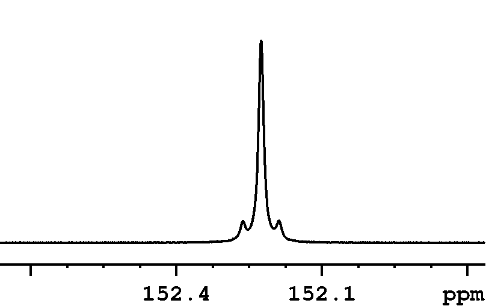


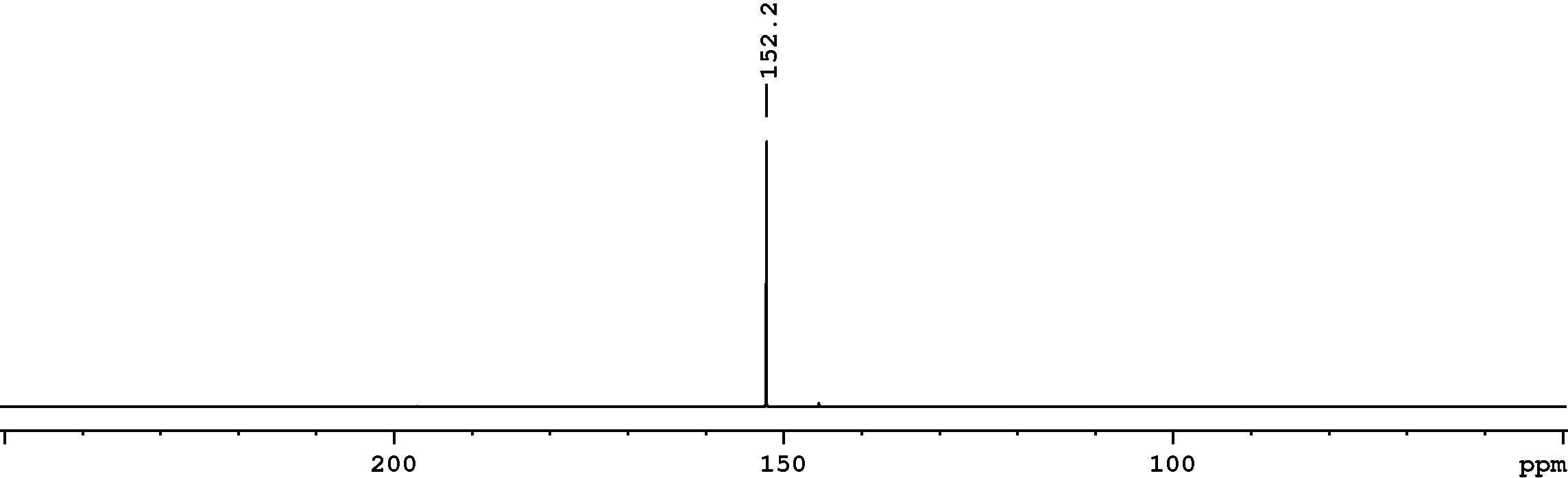


**Figure S6.** ^19^F{^1^H} NMR spectrum (564.7 MHz) of [(BI*i*Pr)WF_6_] **2** recorded in toluene-*d*_8._


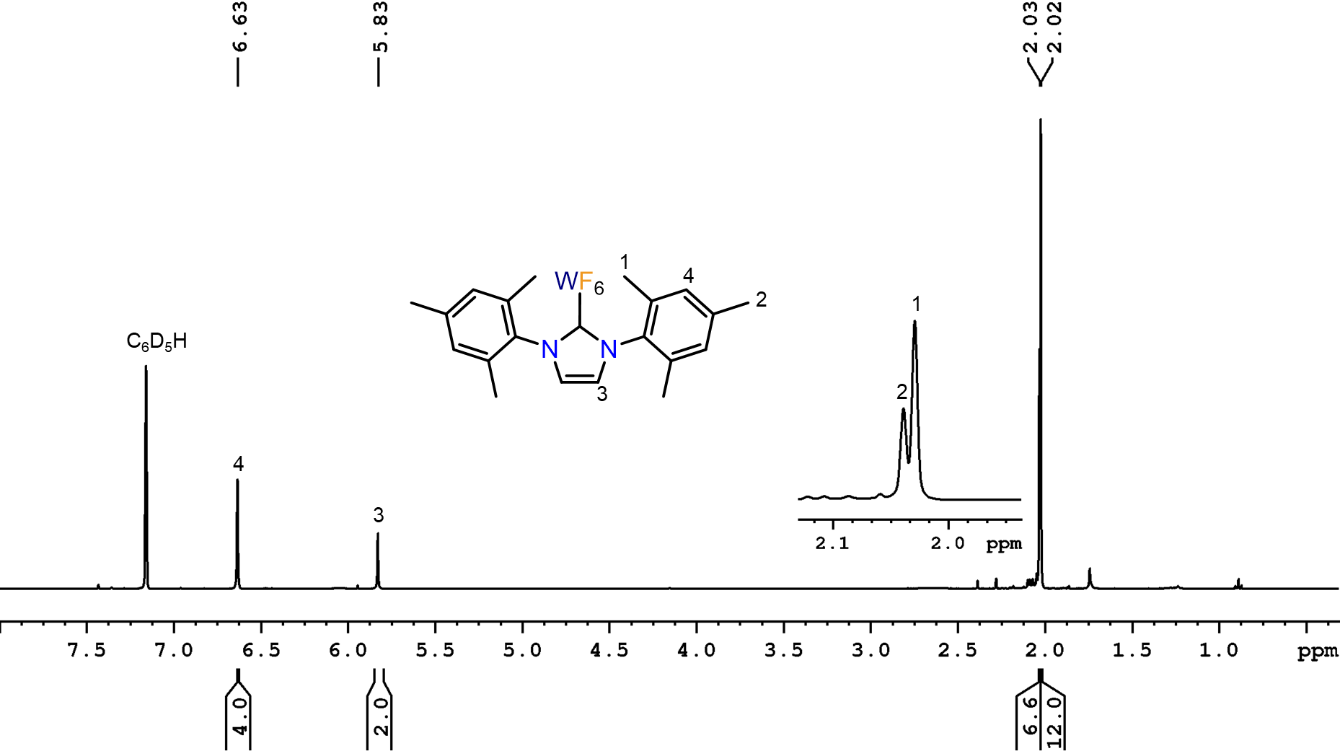


**Figure** **S7.** ^1^H NMR spectrum (400.1 MHz) of [(IMes)WF_6_] **3** recorded in C_6_D_6_.


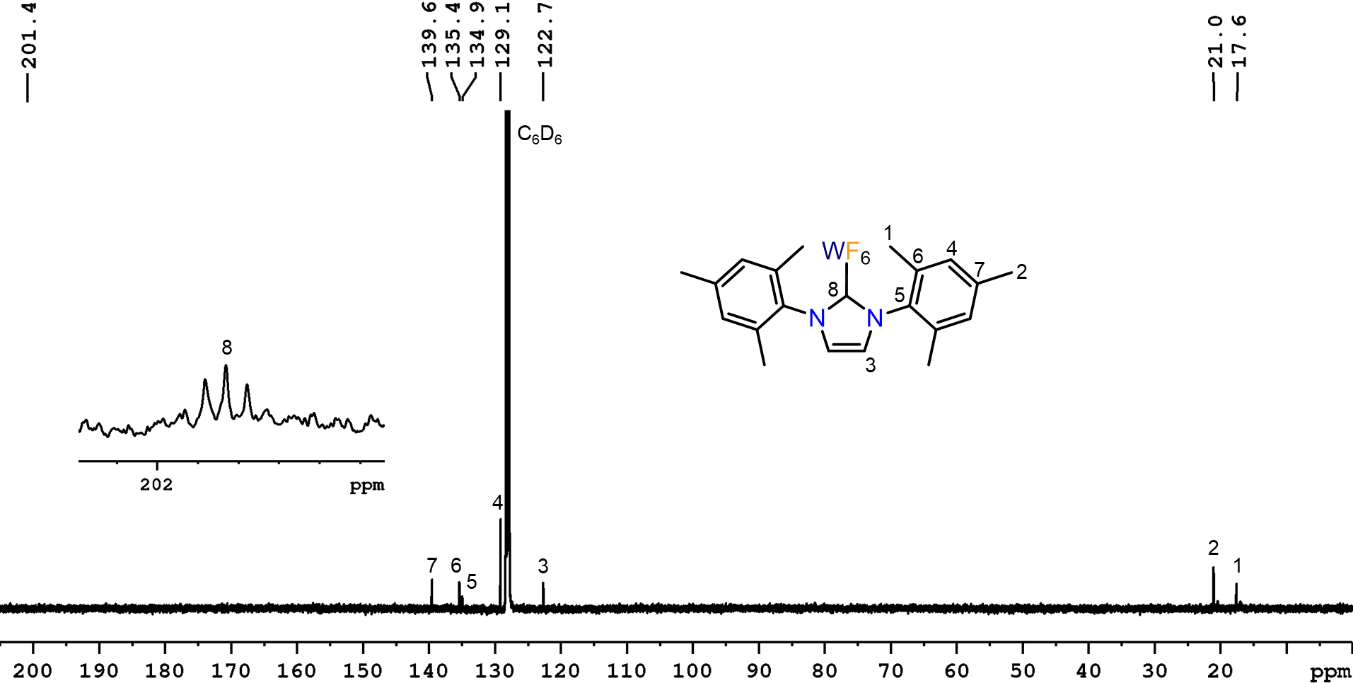


**Figure S8.** ^13^C{^1^H} NMR spectrum (100.7 MHz) of [(IMes)WF_6_] **3** recorded in C_6_D_6_.


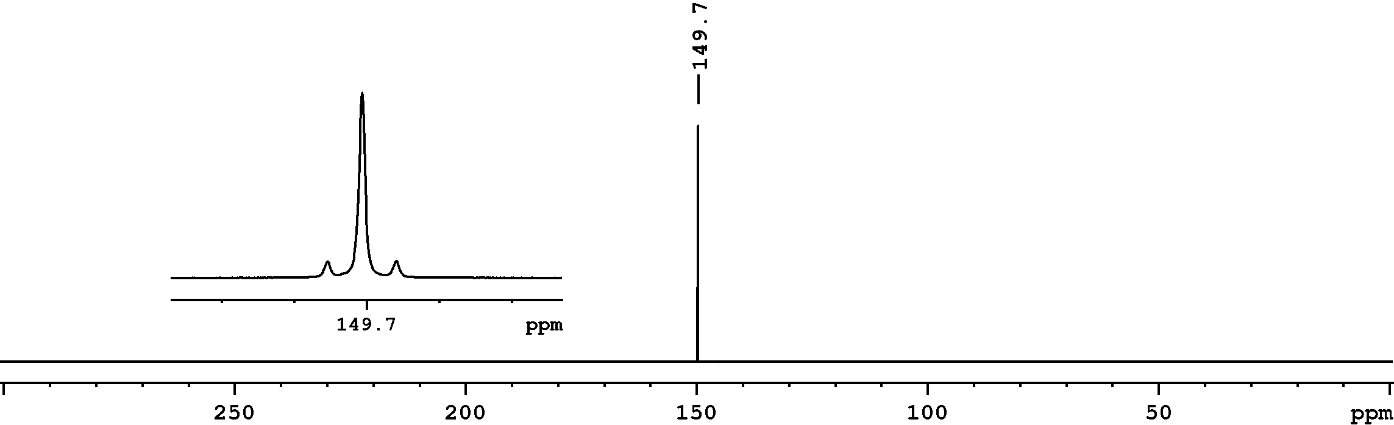


**Figure** **S9.** ^19^F{^1^H} NMR spectrum (376.8 MHz) of [(IMes)WF_6_] **3** recorded in C_6_D_6._


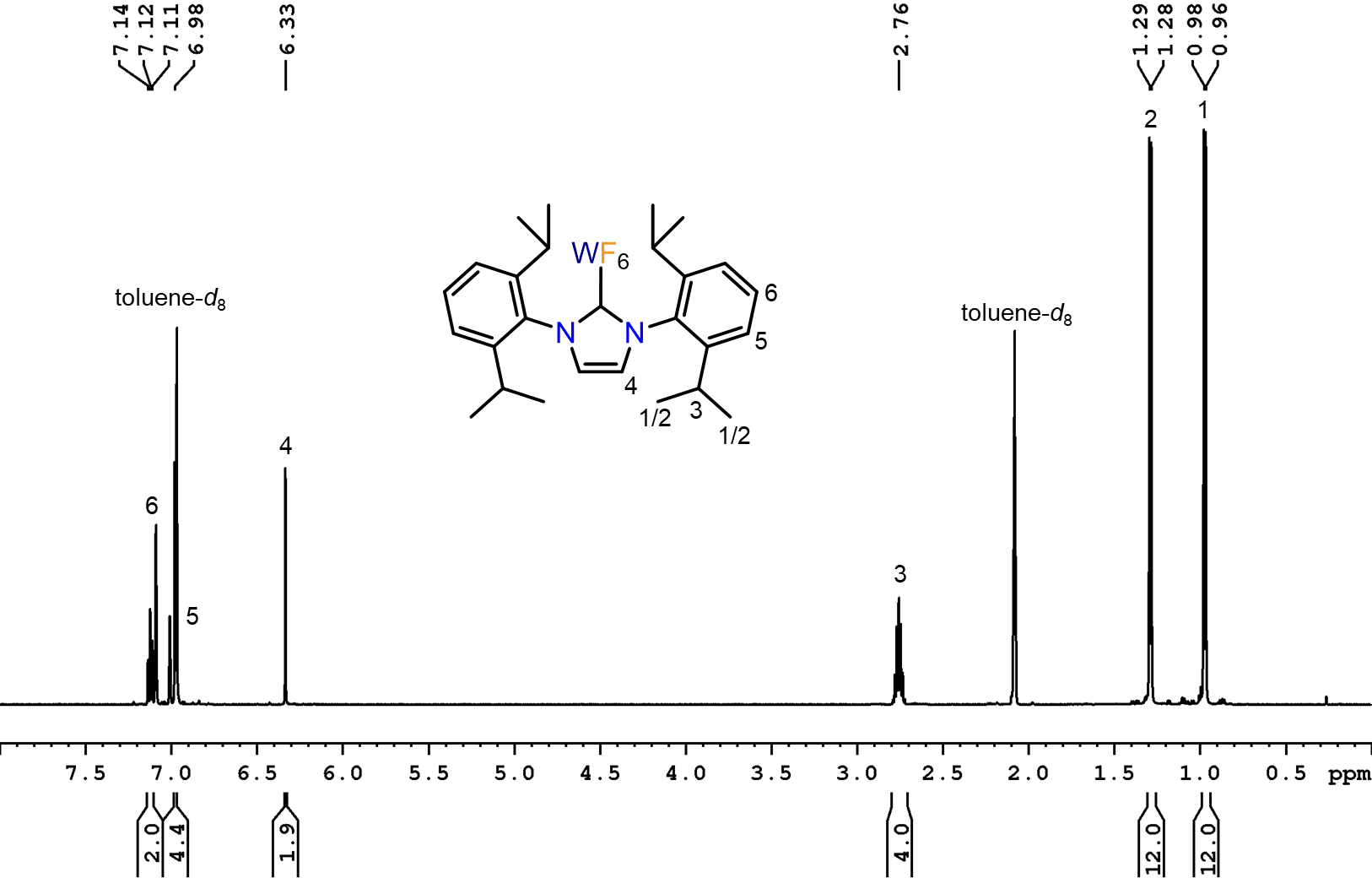


**Figure** **S10.** ^1^H NMR spectrum (600.2 MHz) of [(IDipp)WF_6_] **4** recorded in toluene-d_8_.


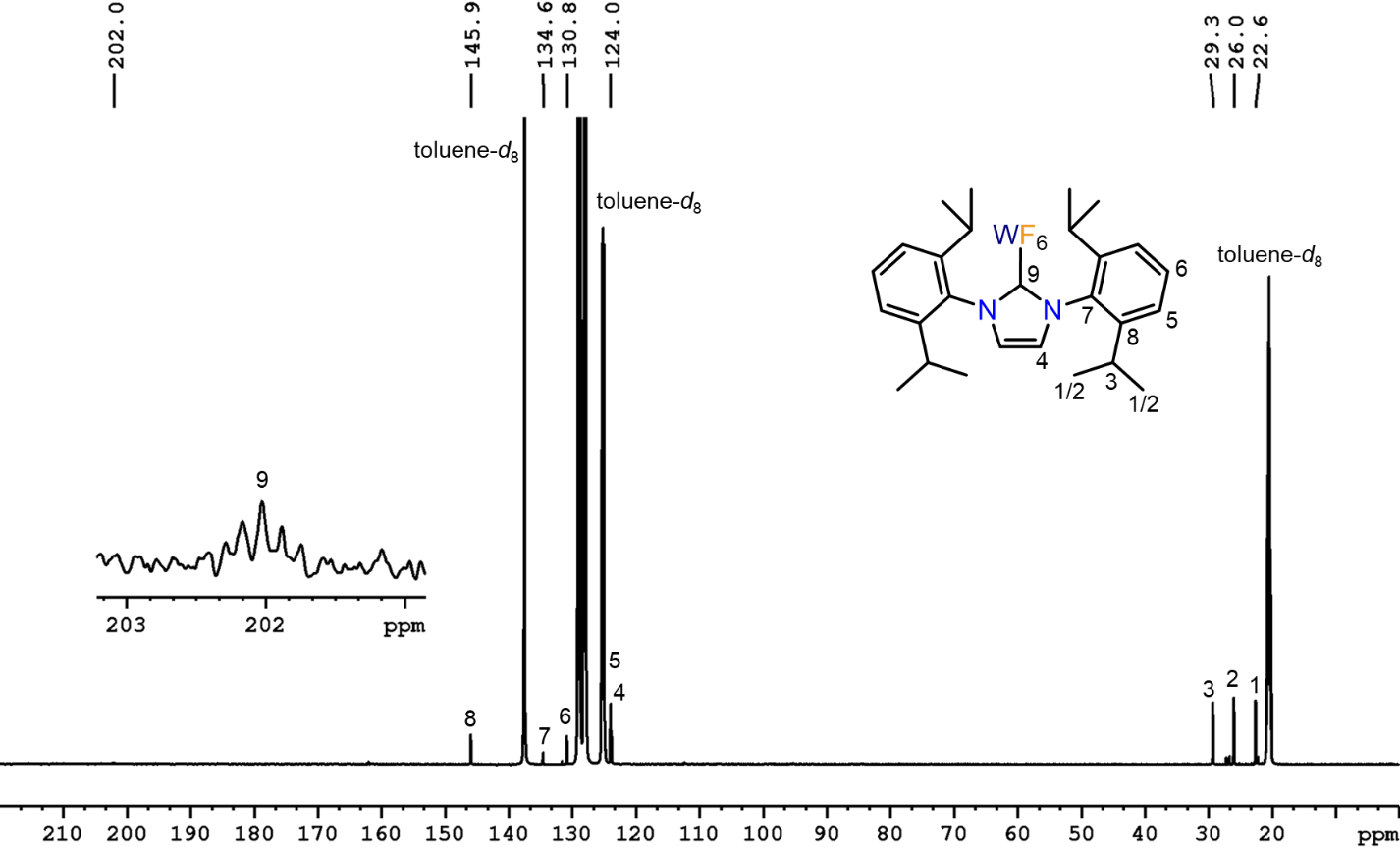


**Figure** **S11**. ^13^C{^1^H} NMR spectrum (150.9 MHz) of [(IDipp)WF_6_] **4** recorded in toluene-d_8_.


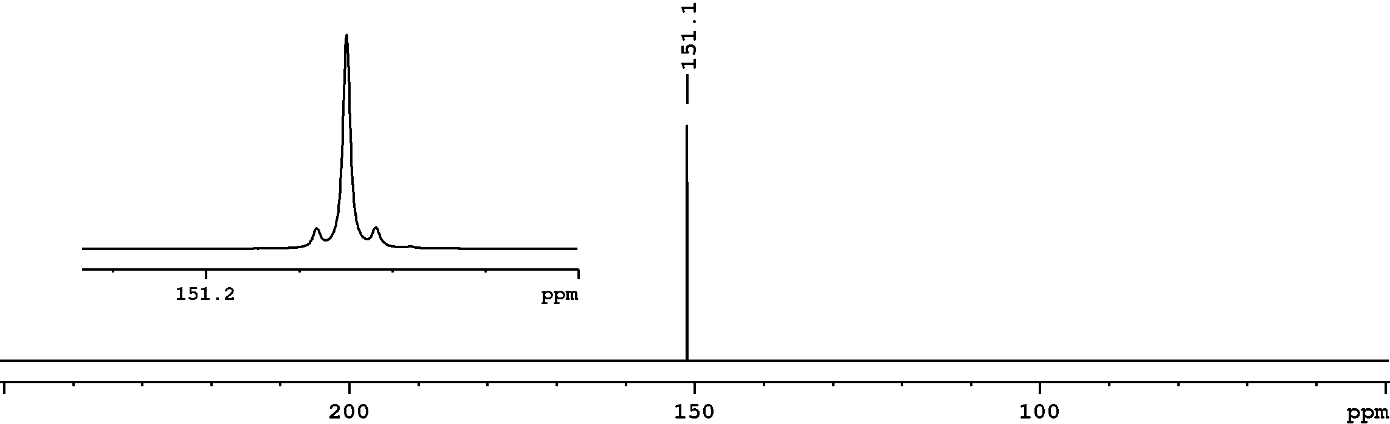


**Figure** **S12.** ^19^F{^1^H} NMR spectrum (564.7 MHz) of [(IDipp)WF_6_] **4** recorded in toluene-d_8_.


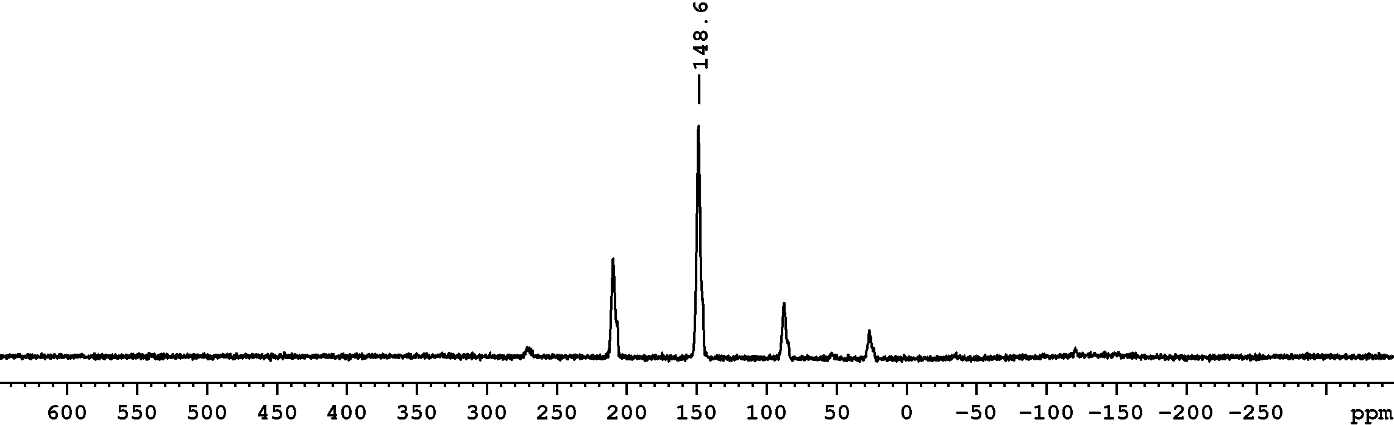


**Figure S13.** ^19^F{^1^H} RSHE/MAS-NMR spectrum (v_rot_ = 23 kHz) of [(IDipp)WF_6_] **4**.


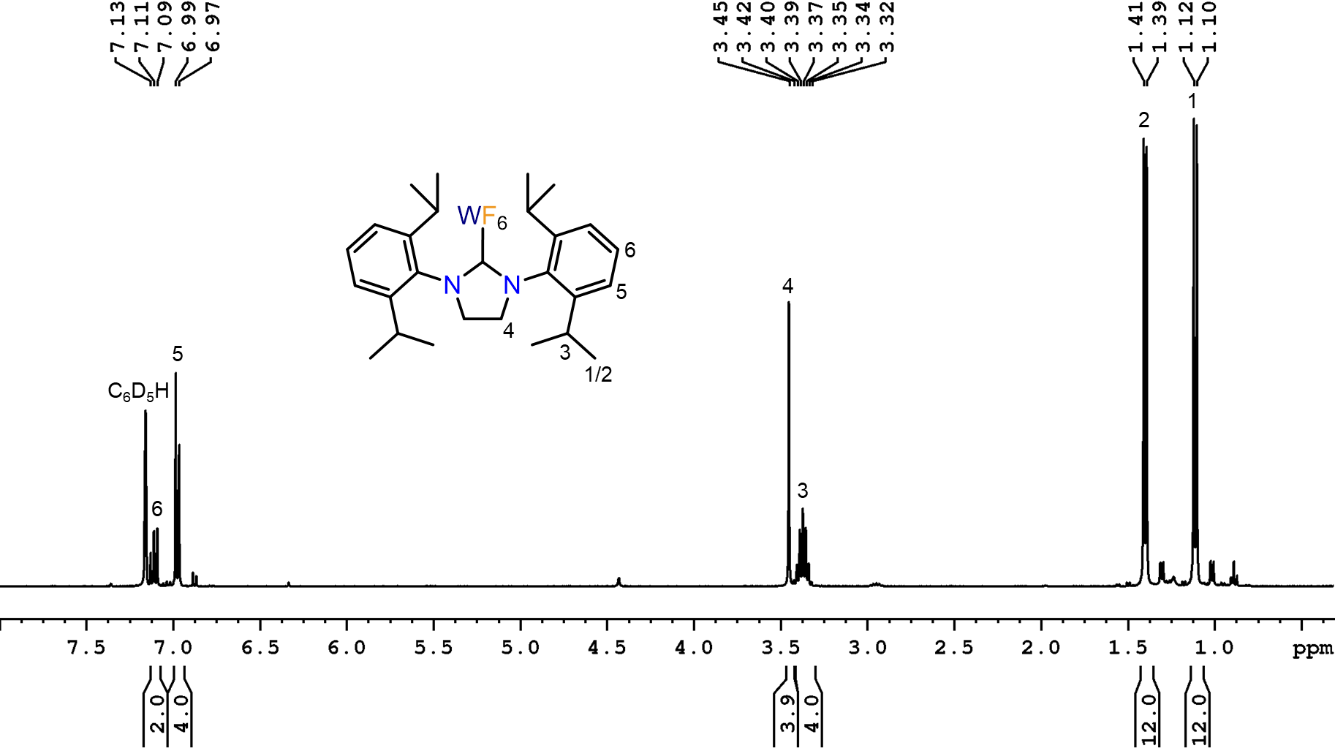


**Figure** **S14.** ^1^H NMR spectrum (400.1 MHz) of [(SIDipp)WF_6_] **5** recorded in C^­^_6_D_6_.


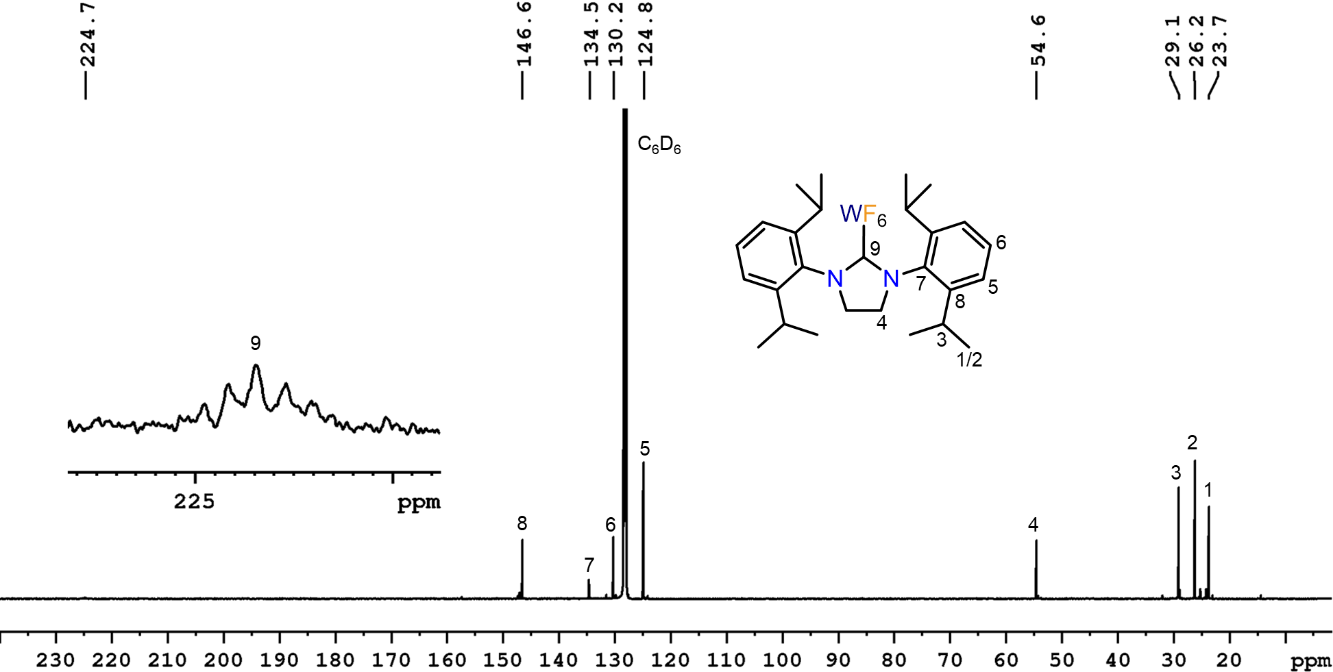


**Figure** **S15.** ^13^C{^1^H} NMR spectrum (125.8 MHz) of [(SIDipp)WF_6_] **5** recorded in C^­^_6_D_6_.


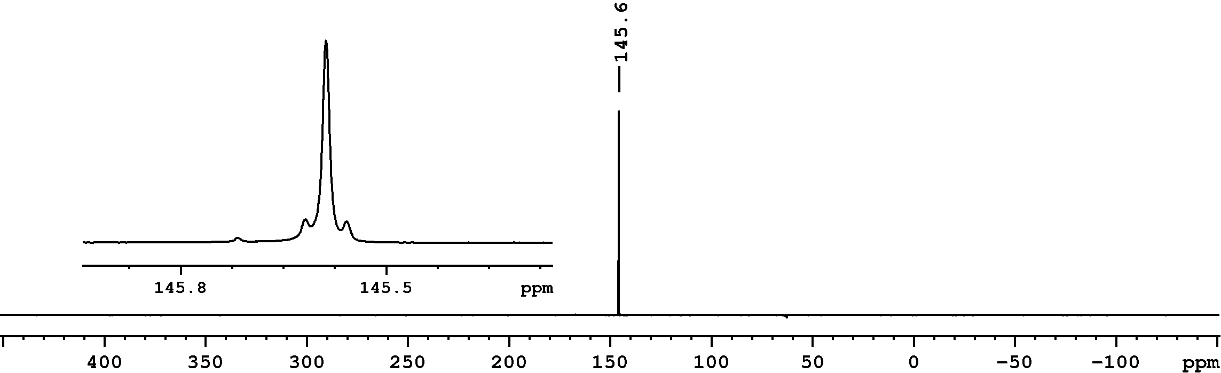


**Figure** **S16.** ^19^F{^1^H} NMR spectrum (470.5 MHz) of [(SIDipp)WF_6_] **5** recorded in C^­^_6_D_6_.


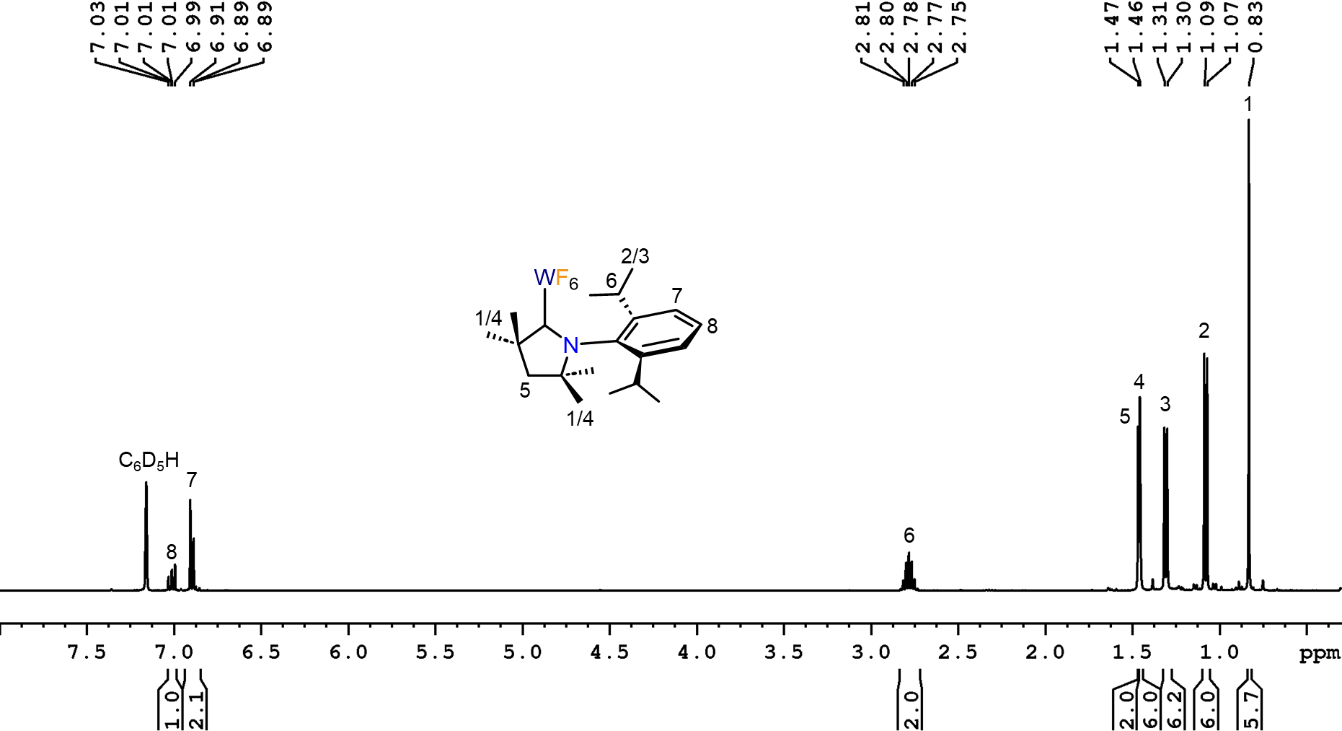


**Figure** **S17.** ^1^H NMR spectrum (400.1 MHz) of [(cAAC^Me^)WF_6_] **6** recorded in C_6_D_6_.


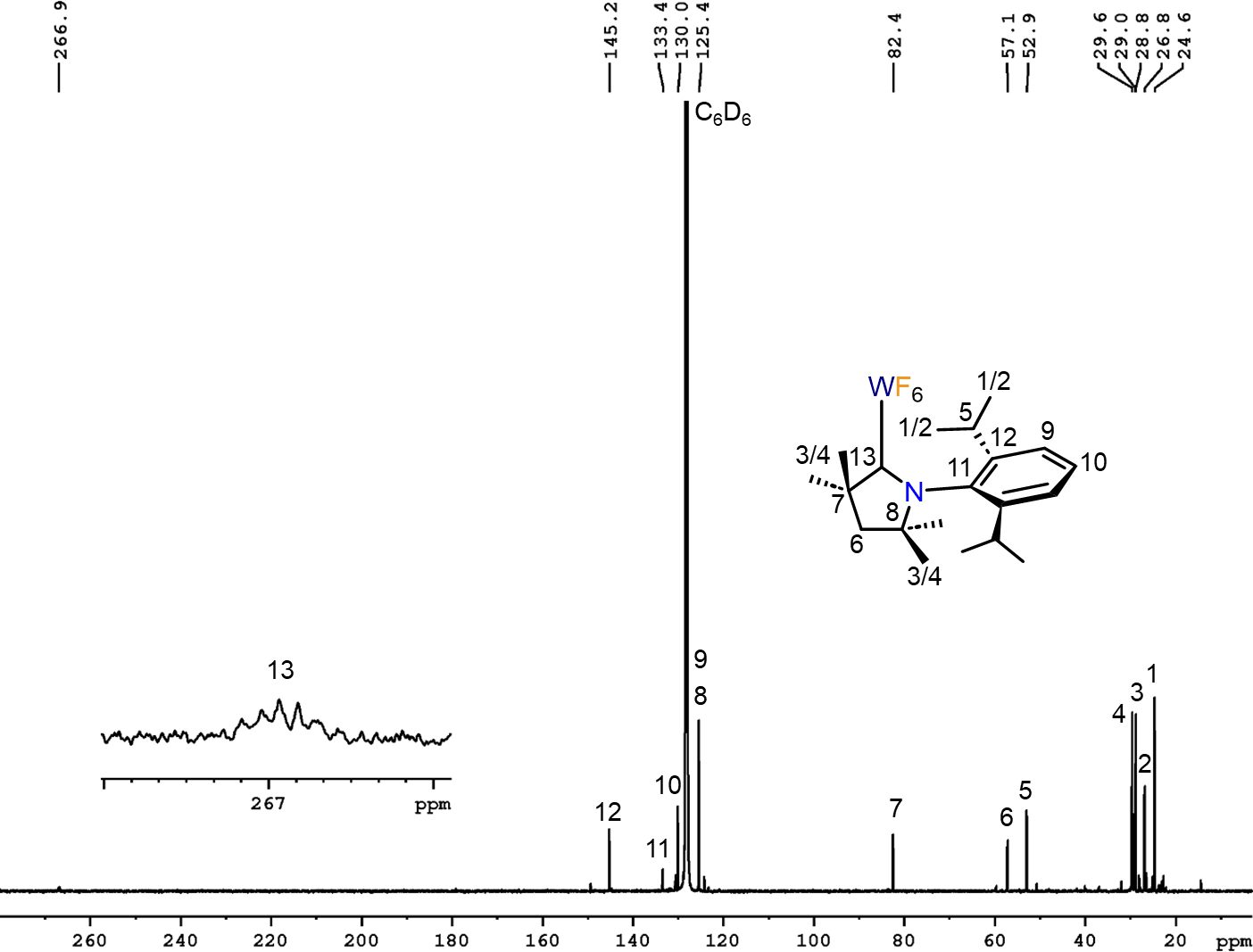


**Figure** **S18.** ^13^C{^1^H} NMR spectrum (100.7 MHz) of [(cAAC^Me^)WF_6_] **6** recorded in C_6_D_6_.


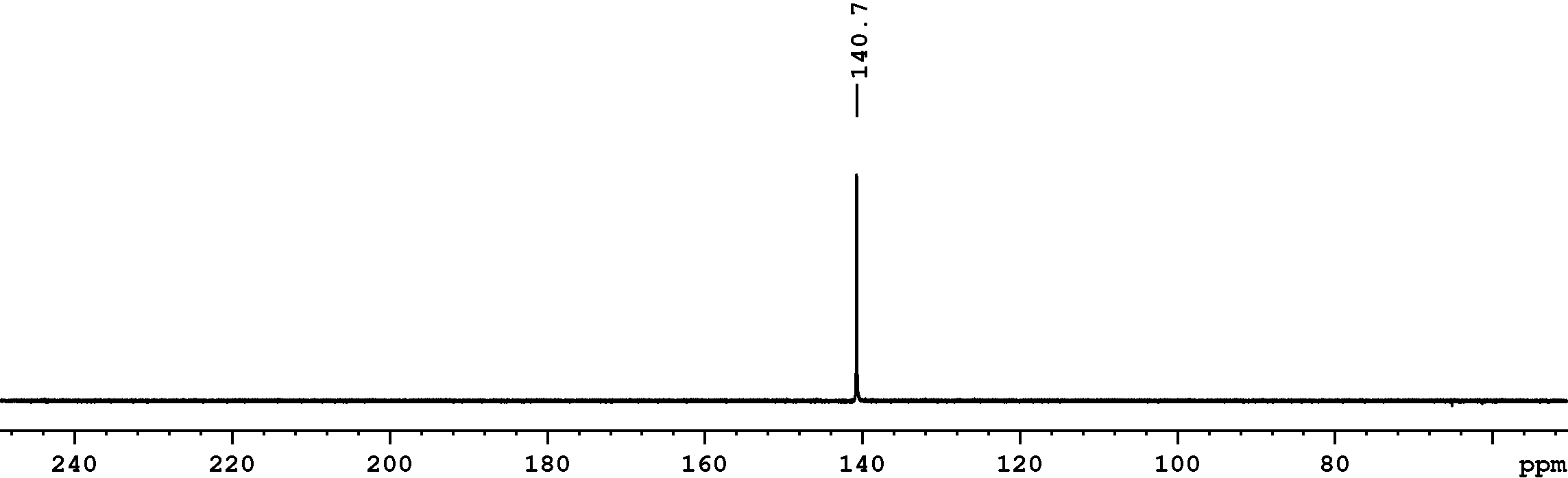


**Figure S19.** ^19^F{^1^H} NMR spectrum (376.8 MHz) of [(cAAC^Me^)WF_6_] **6** recorded in C_6_D_6_.


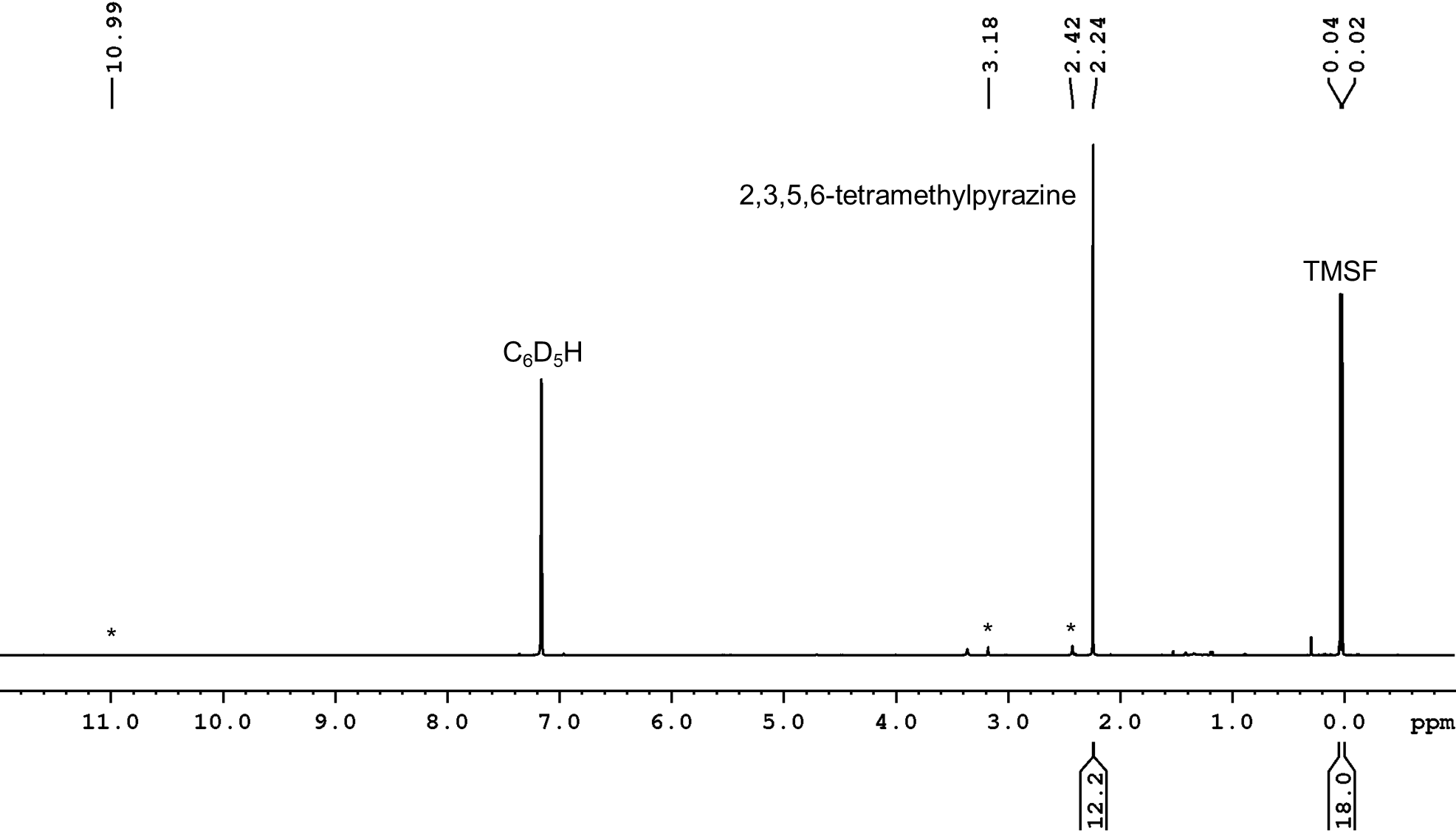


**Figure** **S20.** ^1^H NMR spectrum (400.1 MHz) of the reaction of [(IiPr^Me^)WF_6_] **1** with TMS-py^Me^-TMS recorded in C_6_D_6_ (signals of [(IiPr^Me^)WF_5_] **7** are marked with *).


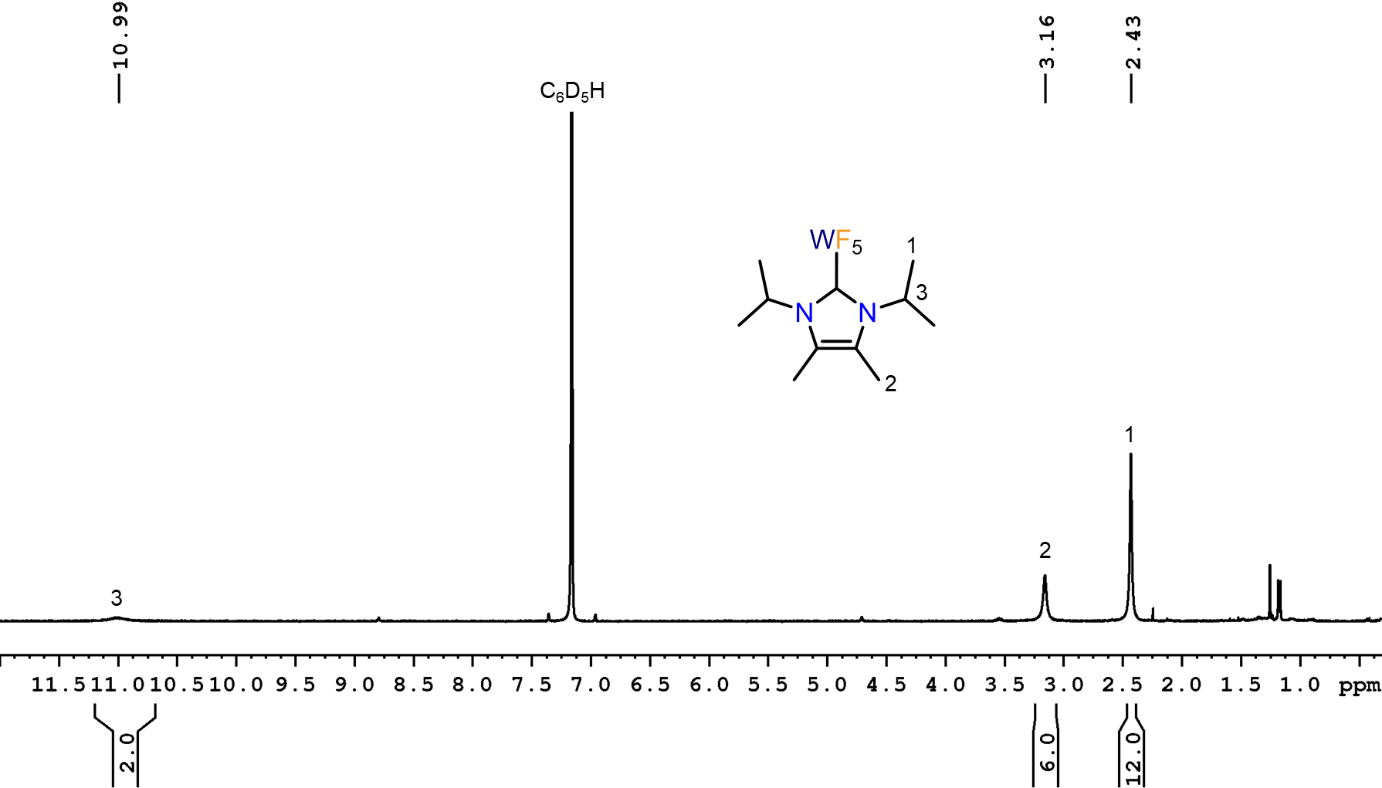


**Figure S21.** ^1^H NMR spectrum (400.1 MHz) of [(IiPr^Me^)WF_5_] **7** recorded in C^­^_6_D_6_.


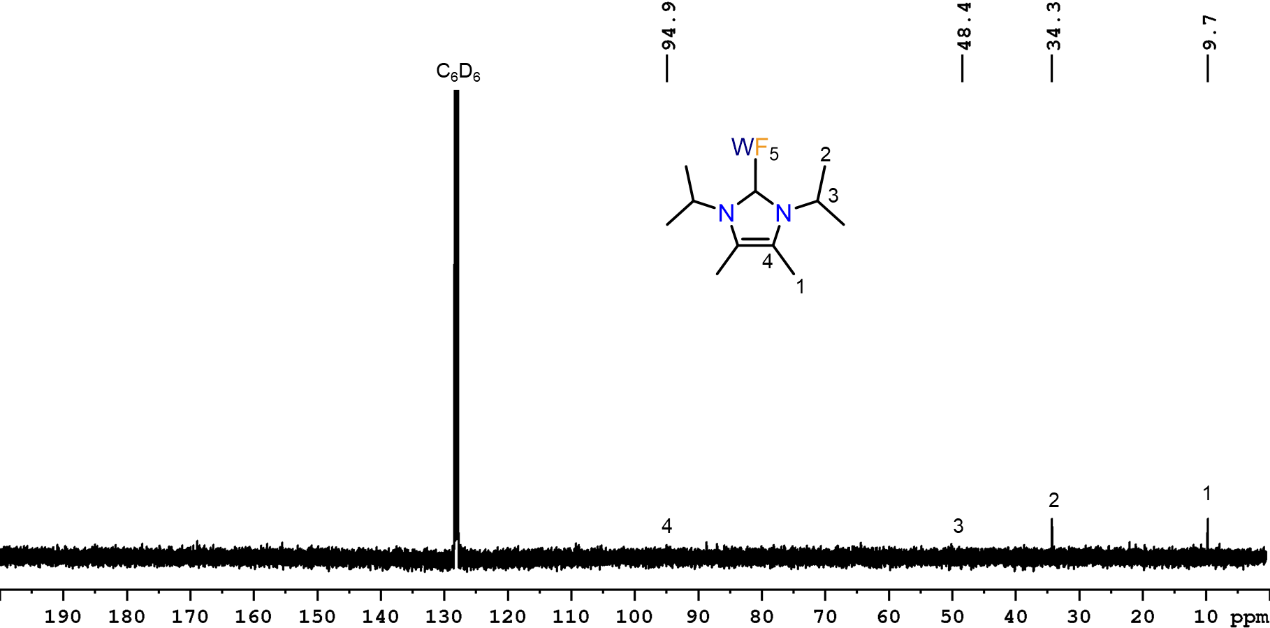


**Figure** **S22.** ^13^C{^1^H} NMR spectrum (376.8 MHz) of [(IiPr^Me^)WF_5_] **7** recorded in C^­^_6_D_6_.


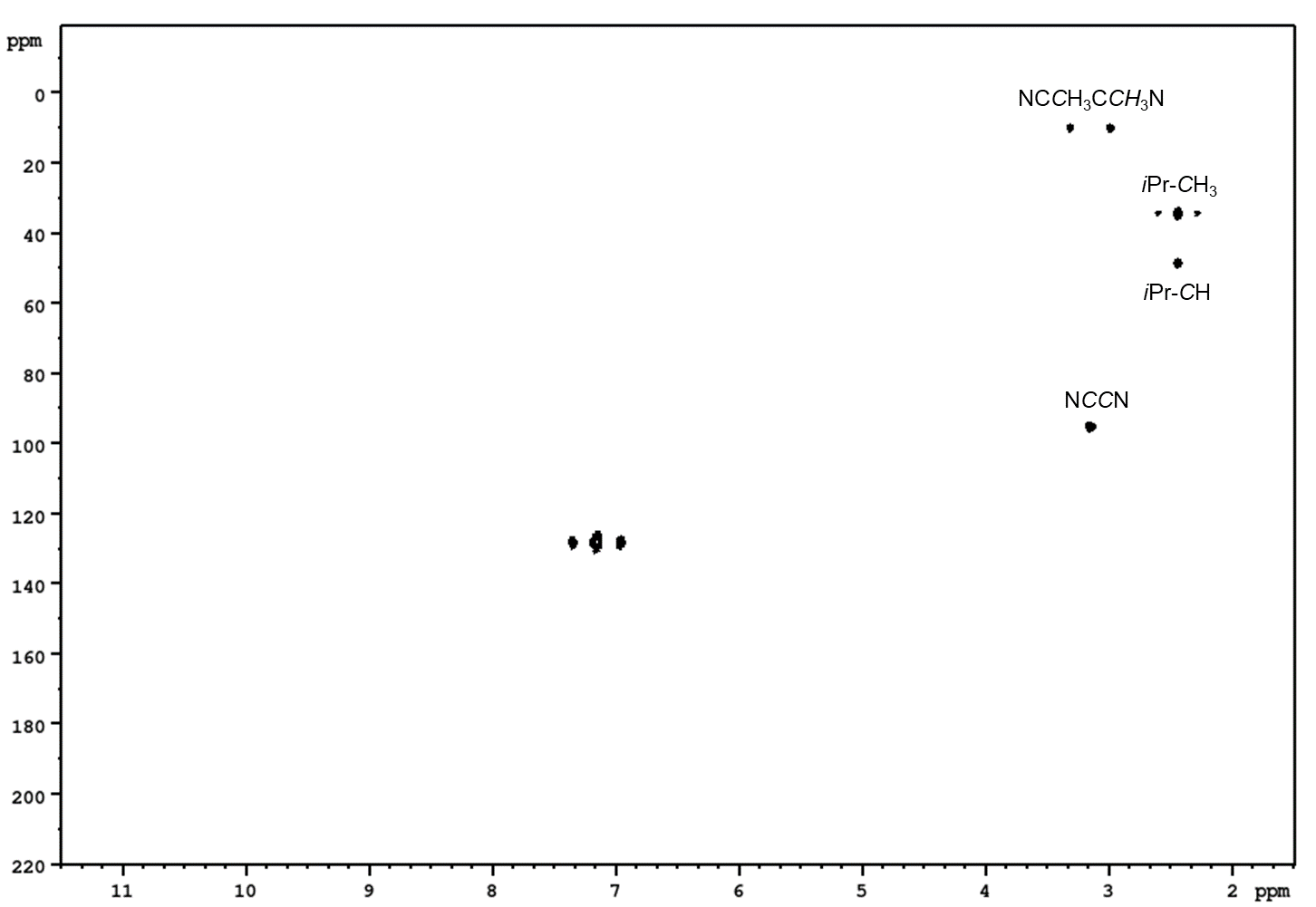


**Figure** **S23.** ^1^H-^13^C{^1^H} HMBC spectrum (400.1 MHz) of [(IiPr^Me^)WF_5_] **7** recorded in C^­^_6_D_6_.


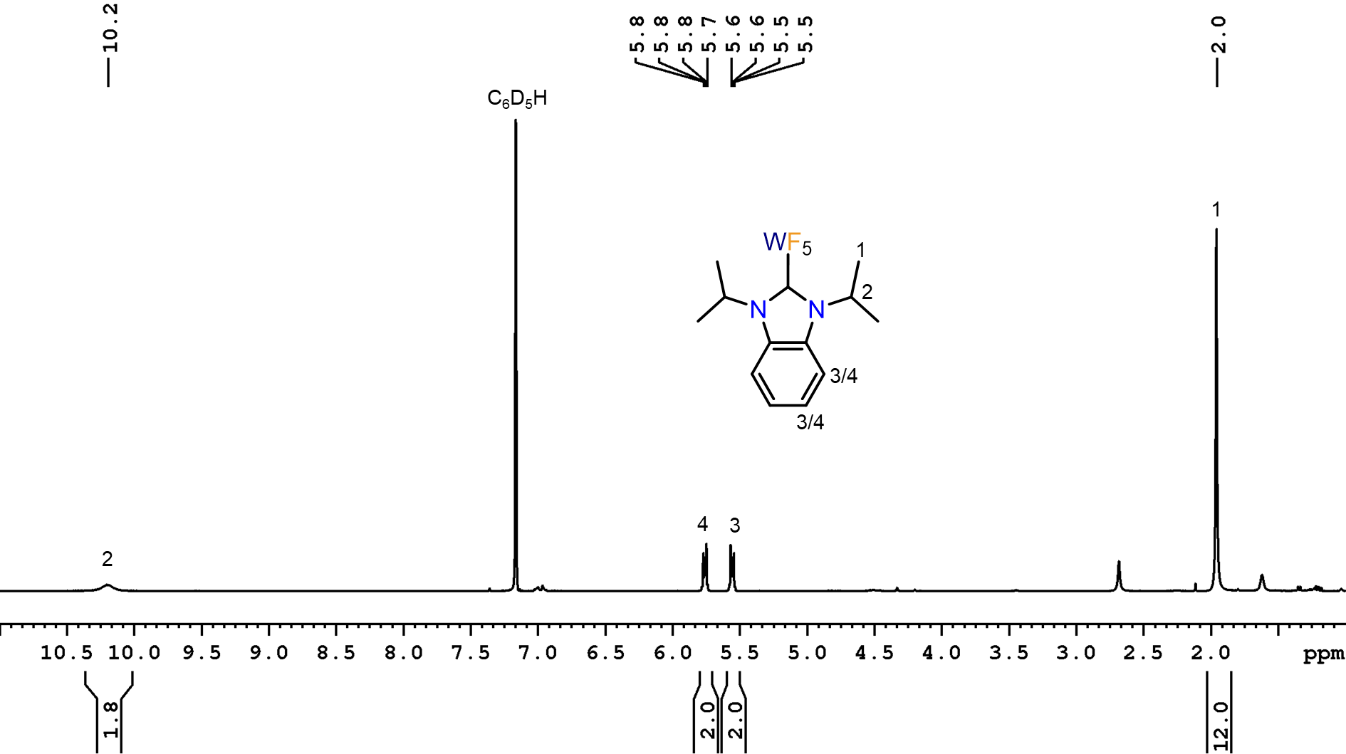


**Figure S24.** ^1^H NMR spectrum (400.1 MHz) of [(BIiPr)WF_5_] **8** recorded in C^­^_6_D_6_.


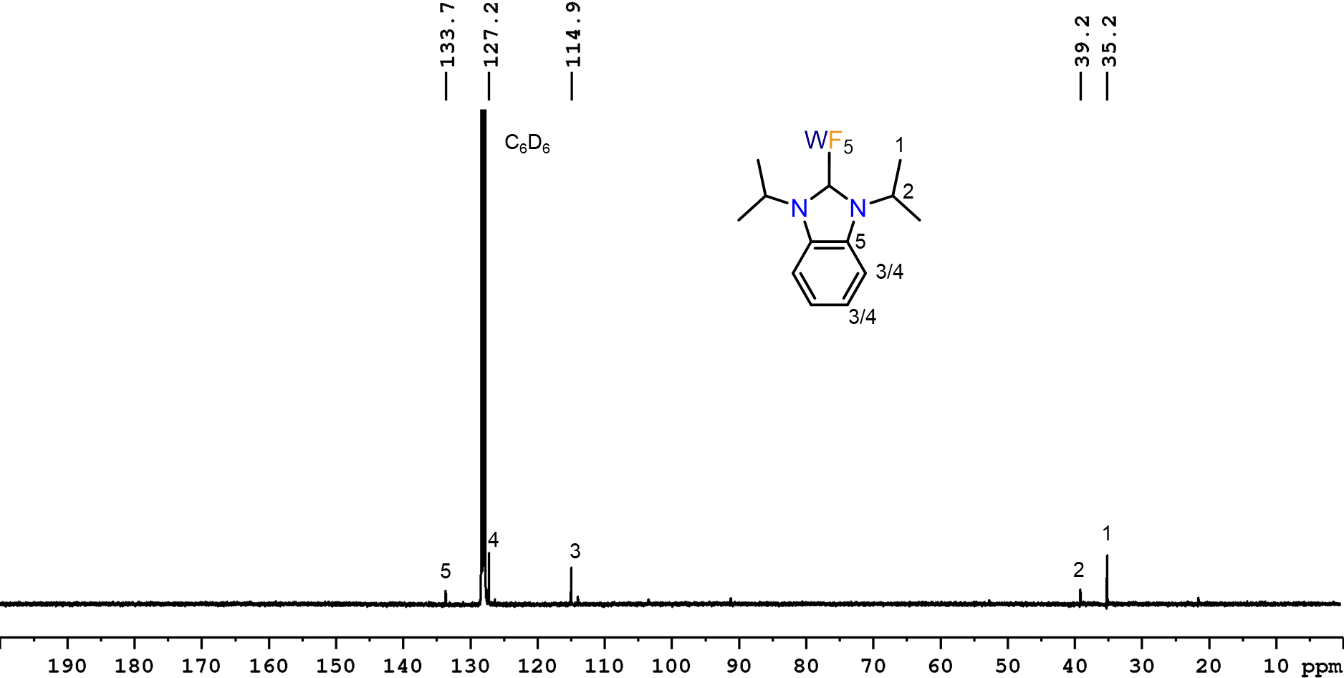


**Figure** **S25.** ^13^C{^1^H} NMR spectrum (376.8 MHz) of [(BIiPr)WF_5_] **8** recorded in C^­^_6_D_6_.


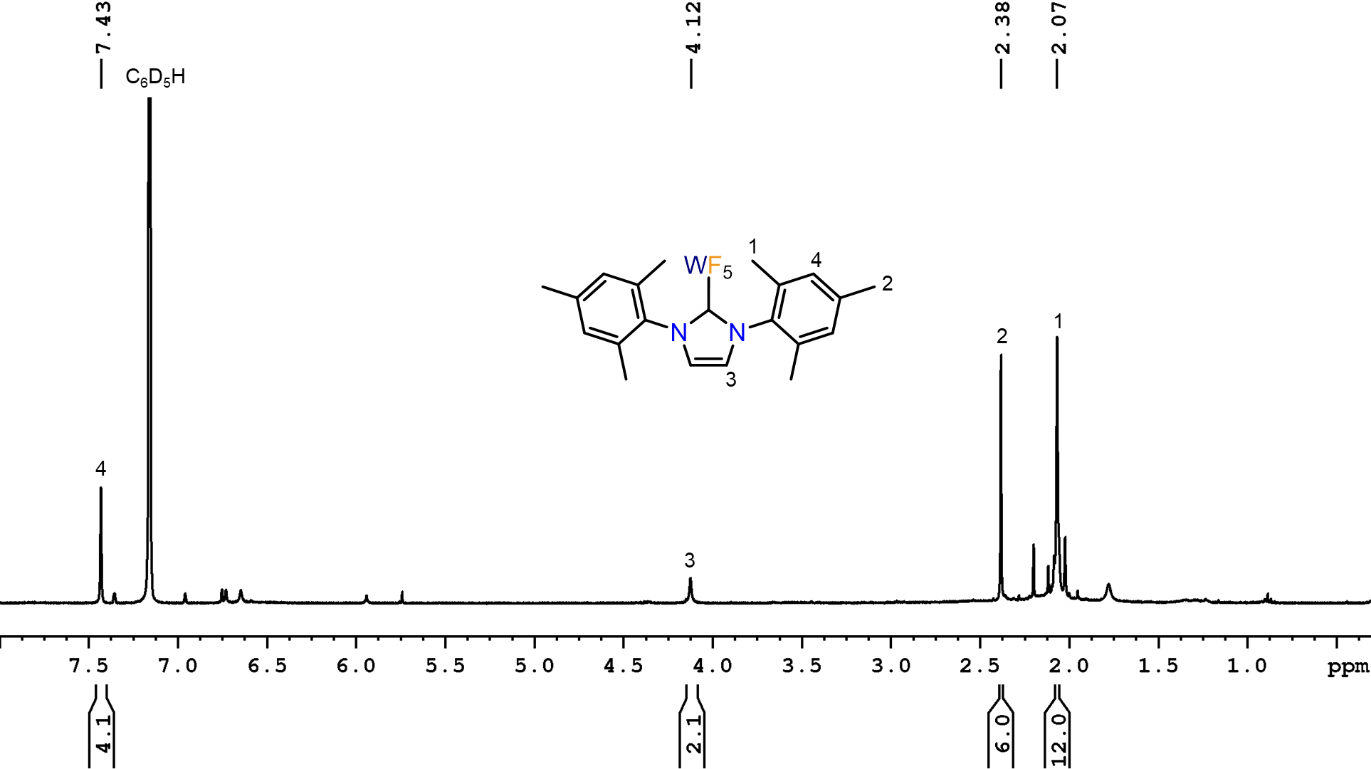


**Figure** **S26.** ^1^H NMR spectrum (400.1 MHz) of [(IMes)WF_5_] **9** recorded in C^­^_6_D_6_.


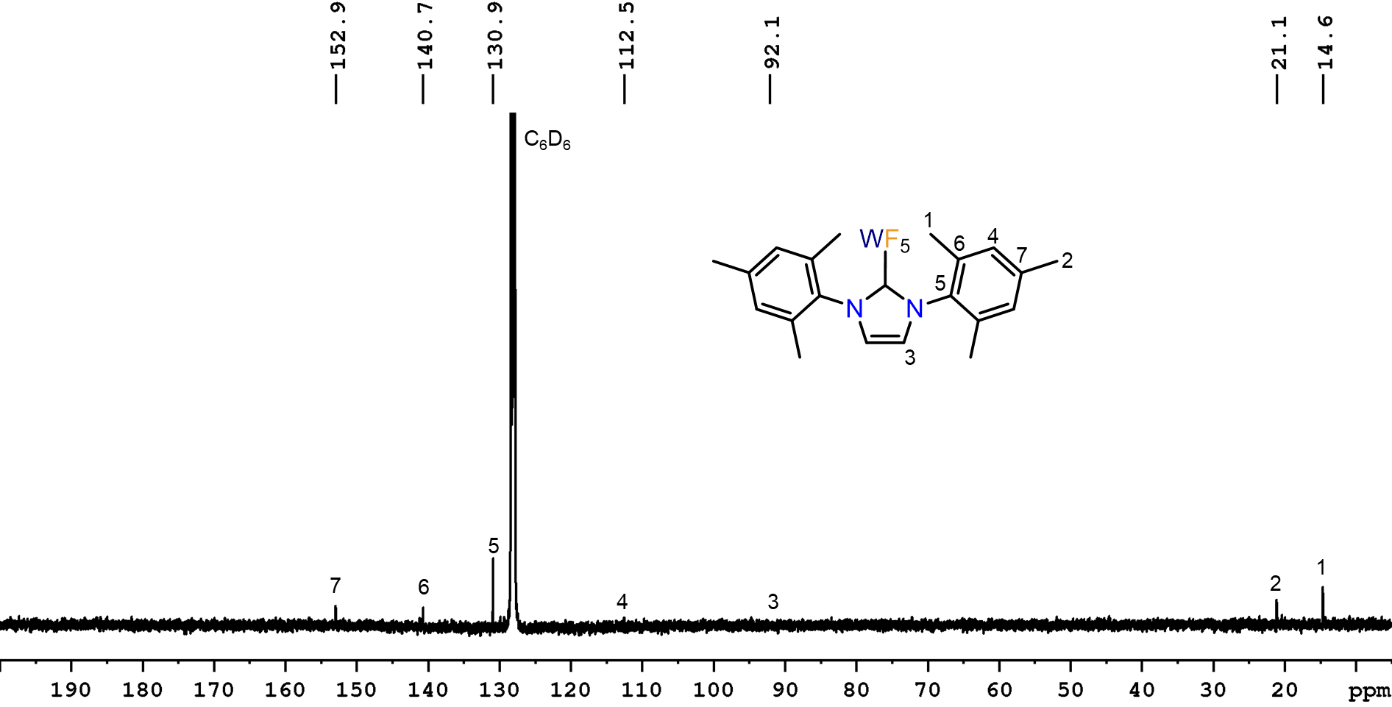


**Figure S27.** ^13^C{^1^H} NMR spectrum (376.8 MHz) of [(IMes)WF_5_] **9** recorded in C^­^_6_D_6_.


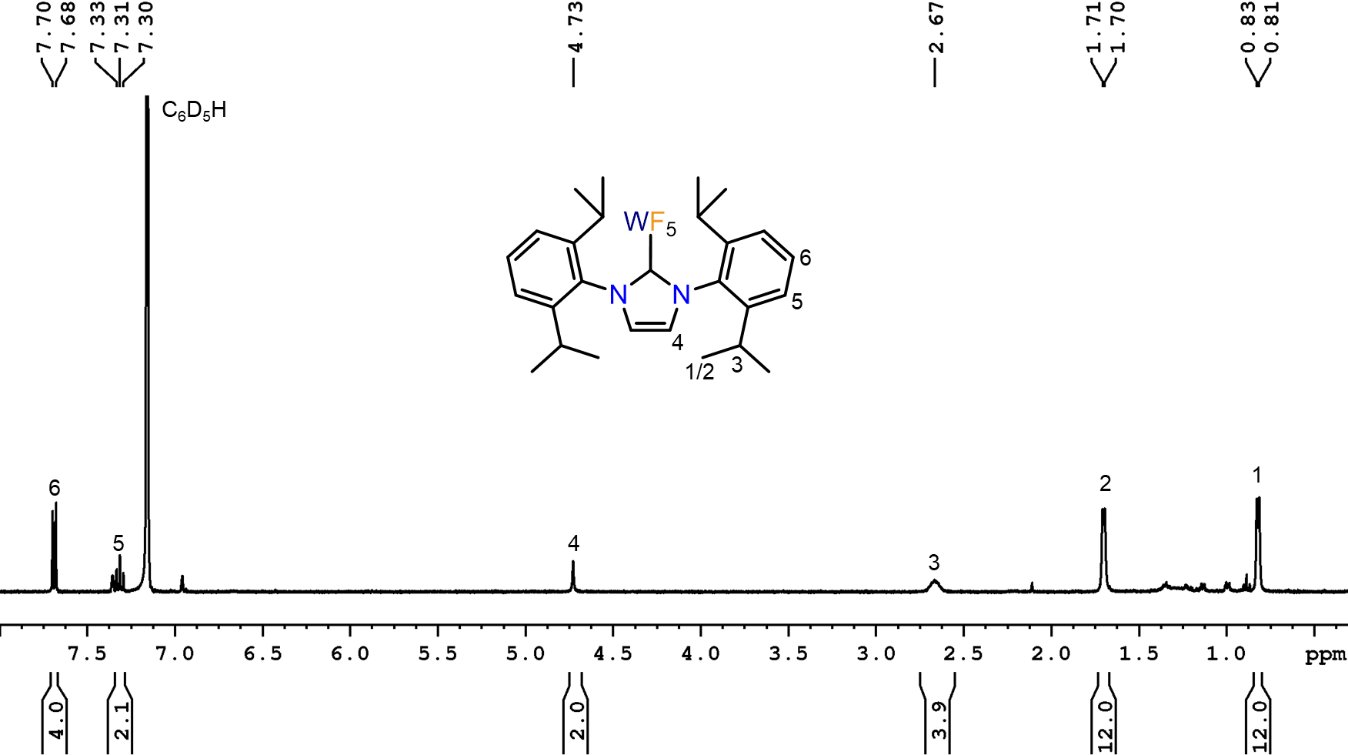


**Figure** **S28.** ^1^H NMR spectrum (400.1 MHz) of [(IDipp)WF_5_] **10** recorded in C^­^_6_D_6_.


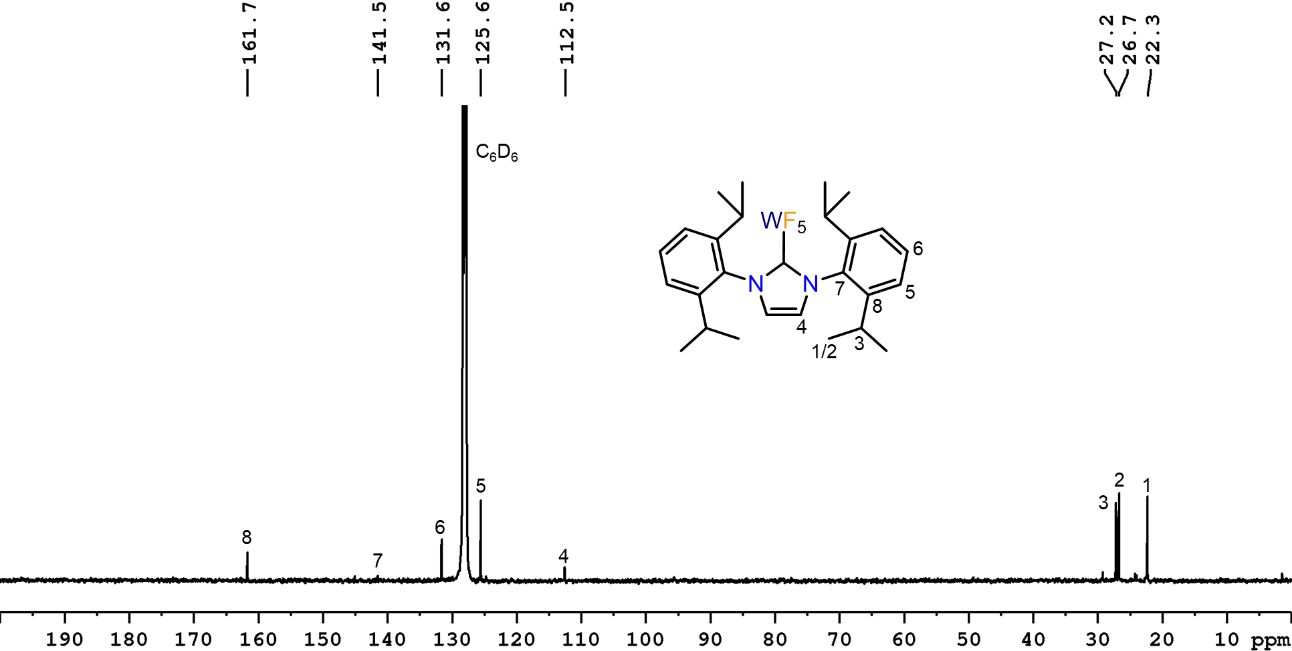


**Figure** **S29.** ^13^C{^1^H} NMR spectrum (376.8 MHz) of [(IDipp)WF_5_] **10** recorded in C^­^_6_D_6_.


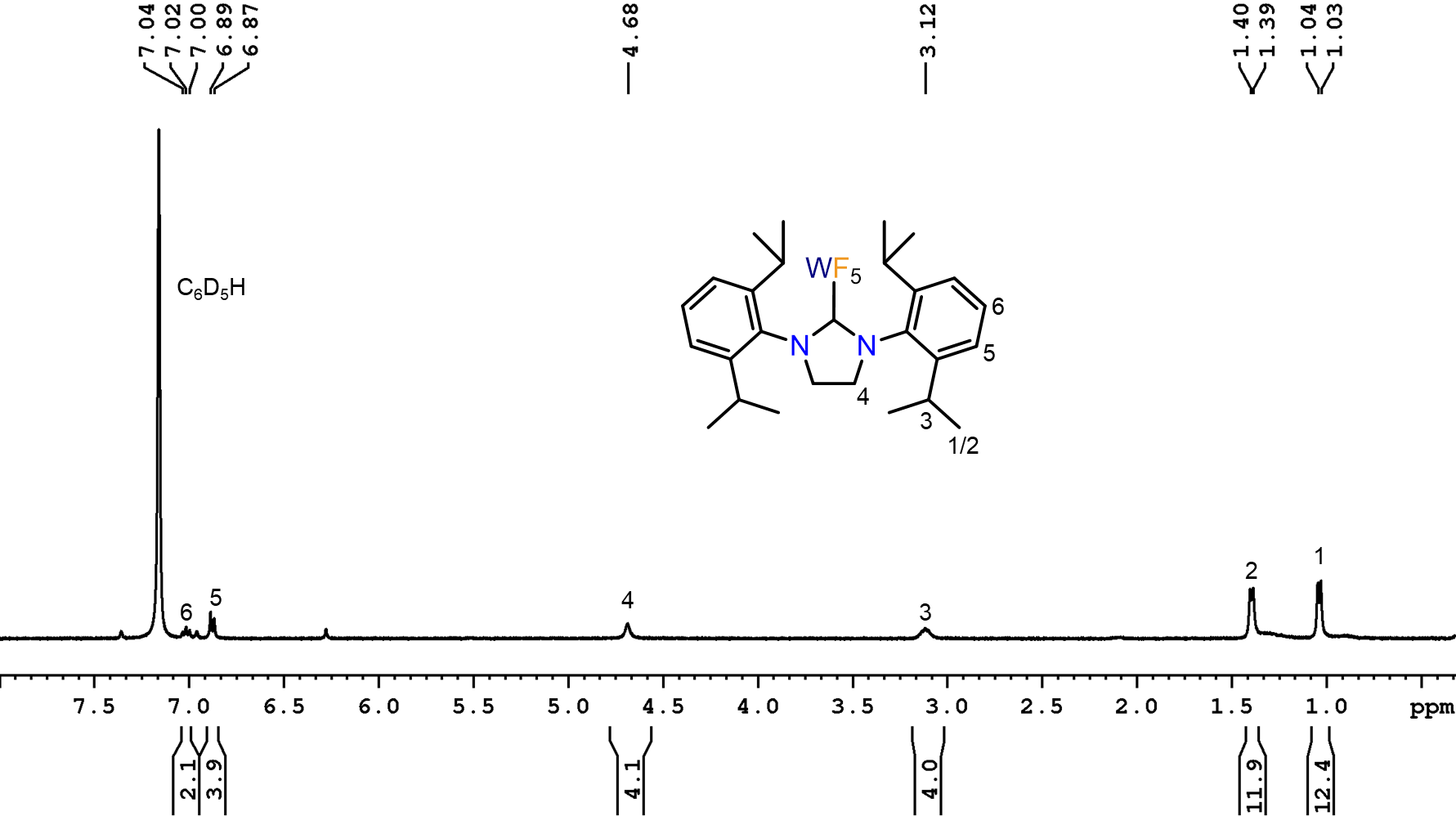


**Figure** **S30.** ^1^H NMR spectrum (400.1 MHz) of [(SIDipp)WF_5_] **11** recorded in C^­^_6_D_6_.


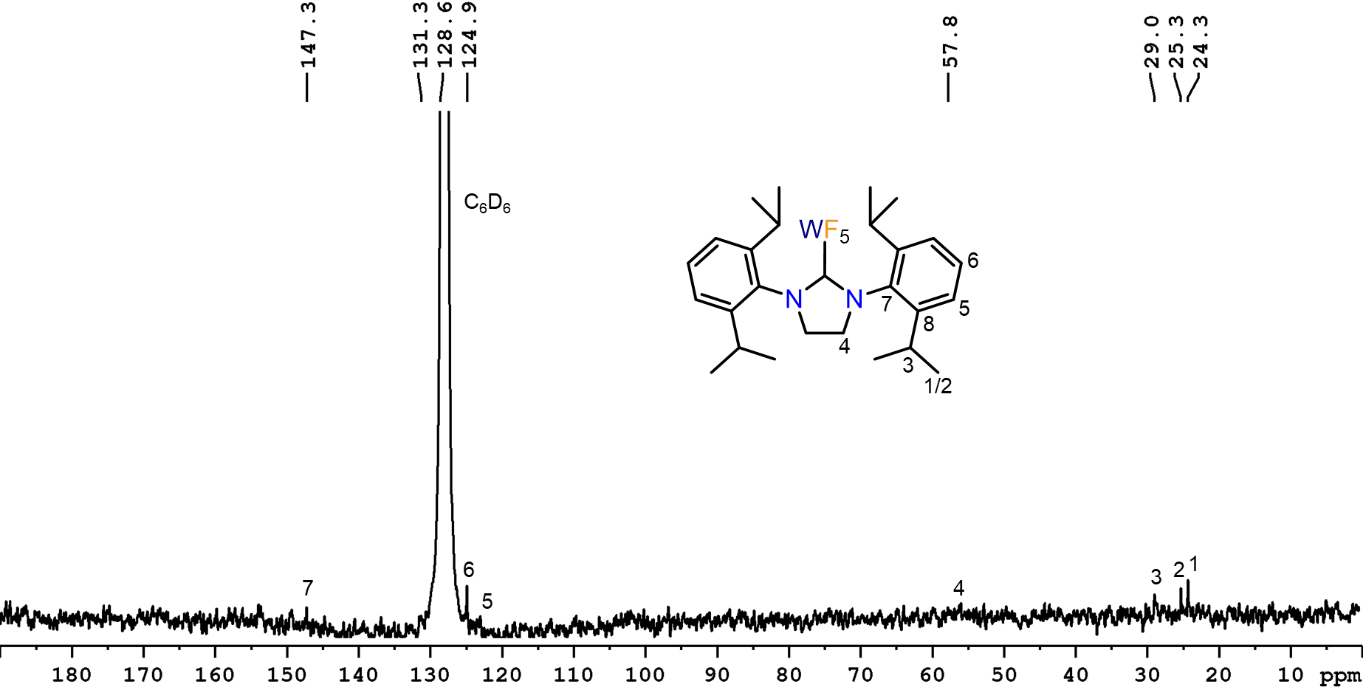


**Figure** **S31.** ^13^C{^1^H} NMR spectrum (376.8 MHz) of [(SIDipp)WF_5_] **11** recorded in C^­^_6_D_6_.

**Crystallographic Details**

Crystal data were collected on a Rigaku XtaLAB Synergy-DW diffractometer with an Hy-Pix-6000HE detector and monochromated Cu−K*α* or Mo−K*α* radiation equipped with an Oxford Cryo 800 cooling unit. Crystals were immersed in a film of perfluoropolyether oil on a glass fiber MicroMount^TM^ (MiTeGen) and data were collected at 100 K. Images were processed with Bruker or CrySalis software packages and equivalent reflections were merged. Corrections for Lorentz-polarization effects and absorption were performed if necessary and the structures were solved by direct methods. Subsequent difference Fourier syntheses revealed the positions of all other non-hydrogen atoms. Structures were solved by using the ShelXTL software package.^[15]^ All non-hydrogen atoms were refined anisotropically. Hydrogen atoms were assigned to idealized geometric positions and were included in structure factors calculations.

Crystallographic data for the structures reported in this paper have been deposited with the Cambridge Crystallographic Data Centre as supplementary publication no.s CCDC-**2425821** (**2**), CCDC-**2425823** (**3**), CCDC-**2425822** (**6**), CCDC-**2425817** (**7**), CCDC-**2425816** (**8**), CCDC-**2425819** (**9**), CCDC-**2425820** (**10**), CCDC-**2425824** (**12**), CCDC-**2425818** (**13**). Copies of the data can be obtained free of charge on application to CCDC.

Crystal data collection of **[(BI*i*Pr)WF_6_]** **2**

**
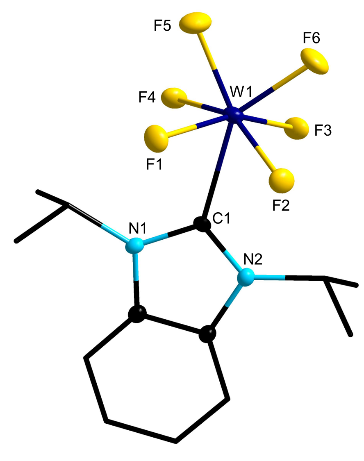
**

**Figure S32.** Molecular structure of [(BIiPr)WF_6_] **2** in the solid state (ellipsoids set at the 50% probability level, carbon atoms except the ones of the carbene ring are shown as wire-and-stick models). Hydrogen atoms are omitted for clarity. Selected bond lengths [Å] and angles [°]: W1−C1 2.264(3), W1−F1 1.865(3), W1−F2 1.872(2), W1−F3 1.875(3), W1−F4 1.877(2), W1−F5 1.881(3), W1−F6 1.869(3), C1−N1 1.334(9), C1−N2 1.367(8); N1-C1-N2 108.4(3), N1-C1-W1 126.5(5), N2-C1-W1 125.1(5), C1-W1-F1 78.3(3), C1-W1-F2 75.41(9), C1-W1-F3 78.6(3), C1-W1-F4 75.11(9), C1-W1-F5 141.6(2), C1-W1-F6 142.3(2), F1-W1-F2 79.99(15), F1-W1-F3 156.92(10), F1-W1-F4 94.38(13), F1-W1-F5 78.71(12), F1-W1-F6 120.96(12), F2-W1-F3 94.33(14), F2-W1-F4 150.51(8), F2-W1-F5 129.50(12), F2-W1-F6 9.84(12), F3-W1-F4 79.52(13), F3-W1-F5 120.57(11), F3-W1-F6 78.66(13), F4-W1-F5 76.48(12), F4-W1-F6 128.87(12), F5-W1-F6 76.03(11).

C_13_H_18_F_6_N_2_W, Mr = 500.14, T = 100.00(2) K, wavelength = 0.71073 Å, orthorhombic space group Pna2_1_, a = 11.50010(10) Å, b = 11.58590(10) Å, c = 11.74150(10) Å, α = 90°, β = 90°, γ = 90°, V = 1564.43(2) Å^3^, Z = 4, ρ(calcd) = 2.123 g/cm^3^ , µ = 7.443 mm^-1^ , F(000) = 952, 120359 reflections in -18 ≤ h ≤ 18, -19 ≤ k ≤ 18, -19 ≤ l ≤ 19 measured in 2.470 < θ < 35.945°, completeness 97.3%, 7073 independent reflections, 6330 reflections observed in [I>2sigma(I)], 215 parameters, 1 restraints, R indices (all data) R1 = 0.0277, wR2 = 0.0510, final R indices [I>2sigma(I)] R1 = 0.0222, wR2 = 0.0495, largest difference peak and hole 1.278 and -1.575 eA^-3^, GooF = 1.053.

Crystal data collection of **[(IMes)WF_6_]** **3**


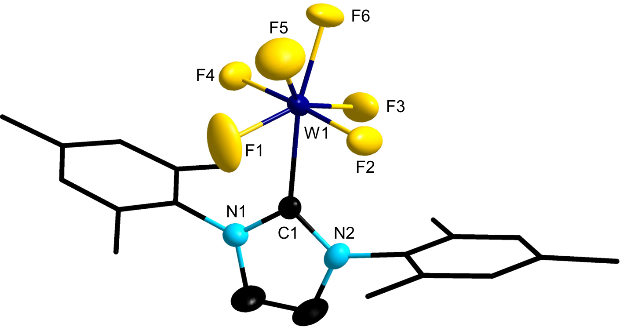


**Figure S33.** Molecular structure of [(IMes)WF_6_] **3** in the solid state (ellipsoids set at the 50% probability level, carbon atoms except the ones of the carbene ring are shown as wire-and-stick models). Hydrogen atoms are omitted for clarity. Selected bond lengths [Å] and angles [°]: W1−C1 2.236(5), W1−F1 1.837(4), W1−F2 1.828(3), W1−F3 1.856(3), W1−F4 1.851(3), W1−F5 1.960(5), W1−F6 1.858(3), C1−N1 1.358(6), C1−N2 1.367(6); N1-C1-N2 105.0(4), N1-C1-W1 127.2(3), N2-C1-W1 127.7(3), C1-W1-F1 74.84(18), C1-W1-F2 85.67(15), C1-W1-F3 76.14(15), C1-W1-F4 90.06(15), C1-W1-F5 136.72(18), C1-W1-F6 155.5(2), F1-W1-F2 96.96(18), F1-W1-F3150.19(18), F1-W1-F4 83.24(18), F1-W1-F5 68.49(19), F1-W1-F6 127.5(2), F2-W1-F3 87.47(16), F2-W1-F4 175.51(15), F2-W1-F5 76.95(18), F2-W1-F6 99.60(15), F3-W1-F4 90.16(17), F3-W1-F5 140.68(17), F3-W1-F6 80.2(2), F4-W1-F5 107.22(18), F4-W1-F6 83.74(15), F5-W1-F6 67.4(2).

C_21_H_24_F_6_N_2_W, Mr = 602.27, T = 100.00(2) K, wavelength = 0.71073 Å, orthorhombic space group Pbca, a = 15.0704(5) Å, b = 15.7503(4) Å, c = 18.0101(4) Å, α = 90°, β = 90°, γ = 90°, V = 4274.9(2) Å^3^, Z = 8, ρ(calcd) = 1.872 g/cm^3^ , µ = 5.428 mm^-1^ , F(000) = 2336, 81011 reflections in -18 ≤ h ≤ 18, -18 ≤ k ≤ 18, -21 ≤ l ≤ 21 measured in 2.186 < θ < 25.122°, completeness 100.0%, 3805 independent reflections, 3213 reflections observed in [I>2sigma(I)], 277 parameters, 0 restraints, R indices (all data) R1 = 0.0372, wR2 = 0.0789, final R indices [I>2sigma(I)] R1 = 0.0299, wR2 = 0.0757, largest difference peak and hole 1.820 and -1.209 eA^-3^, GooF = 1.053.

Crystal data collection of **[(IDipp)WF_6_] 4**

**
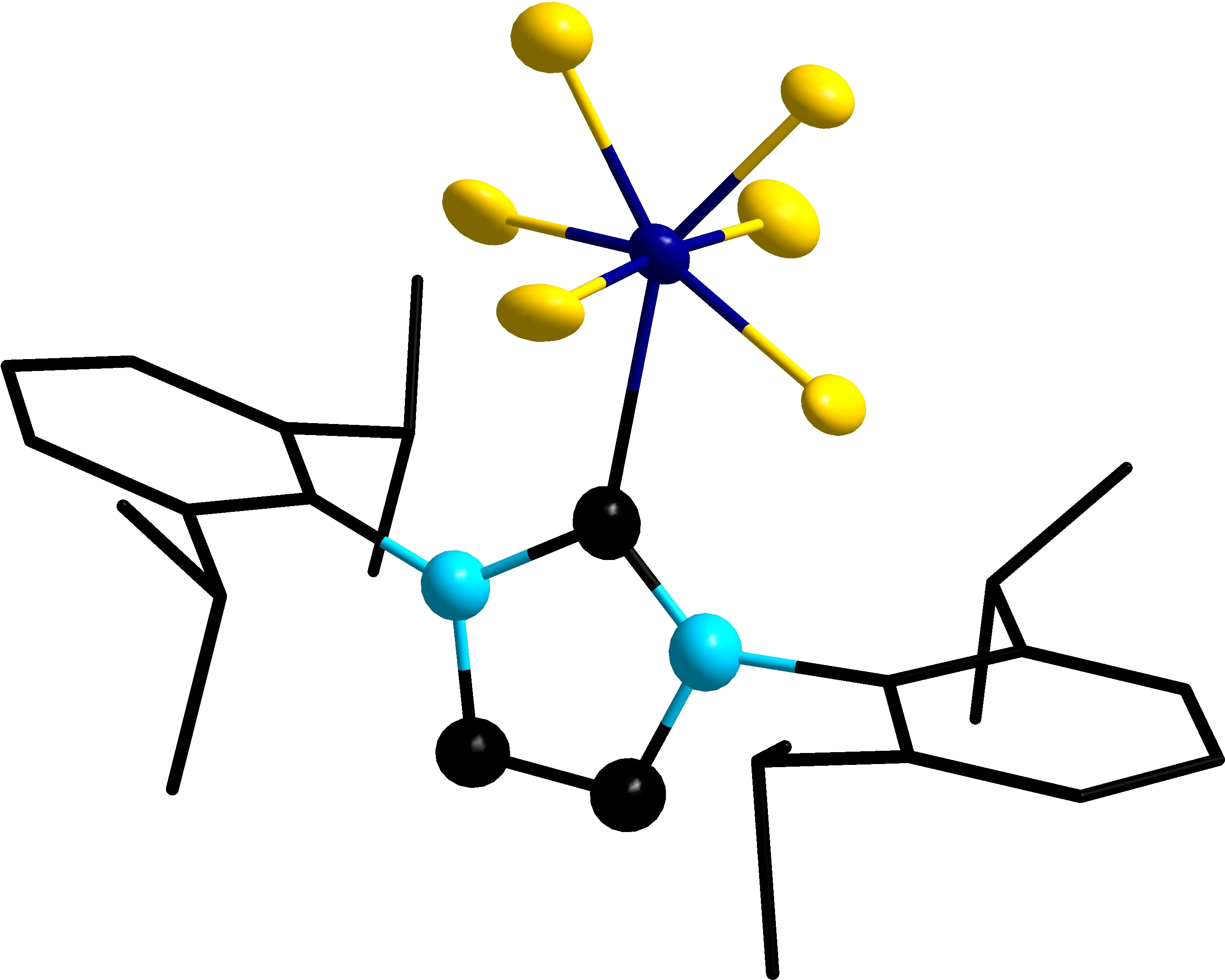
**

**Figure S34.** Molecular structure of [(IDipp)WF_6_] **4** in the solid state (ellipsoids set at the 30% probability level, carbon atoms except the ones of the carbene ring are shown as wire-and-stick models). Due to bad crystal quality the structural data is sufficient for proof of connectivity but insufficient for detailed discussion of bond parameters.

C_54_H_70_F_12_N_4_W_2_, Mr = 1370.84, T = 100.00(2) K, wavelength = 1.54184 Å, tetragonal space group P4/n, a = 12.58450(10) Å, b = 12.58450(10) Å, c = 8.8584(2) Å, α = 90°, β = 90°, γ = 90°, V = 1402.90(4) Å^3^, Z = 2, ρ(calcd) = 3.245 g/cm^3^ , µ = 16.276 mm^-1^ , F(000) = 1356, 27182 reflections in -15 ≤ h ≤ 15, -15 ≤ k ≤ 13, -10 ≤ l ≤ 10 measured in 4.970 < θ < 75.195°, completeness 98.6%, 1433 independent reflections, 1383 reflections observed in [I>2sigma(I)], 207 parameters, 507 restraints, R indices (all data) R1 = 0.0721, wR2 = 0.1965, final R indices [I>2sigma(I)] R1 = 0.0712, wR2 = 0.1952, largest difference peak and hole 3.560 and -3.769 eA^-3^, GooF = 1.068.

Crystal data collection of **[(cAAC^Me^)WF_6_] 6**


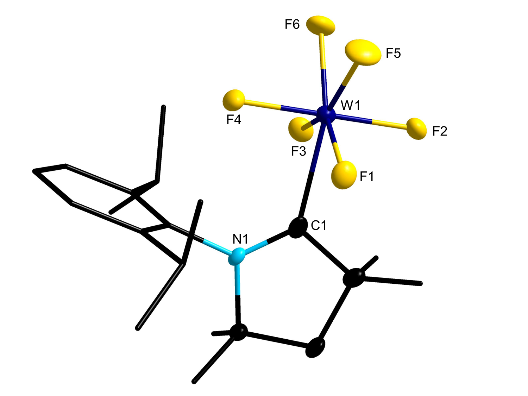


**Figure S35.** Molecular structure of [(cAAC^Me^)WF_6_] **6** in the solid state (ellipsoids set at the 50% probability level, carbon atoms except the ones of the carbene ring are shown as wire-and-stick models). Hydrogen atoms are omitted for clarity. Selected bond lengths [Å] and angles [°]: W1−C1 2.257(7), W1−F1 1.909(4), W1−F2 1.846(4), W1−F3 1.904(4), W1−F4 1.835(4), W1−F5 1.882(4), W1−F6 1.887(4), C1−N1 1.301(8), C1−F1 2.3430(77), C1−F3 2.3530(76); N1-C1-W1 128.6(5), C1-W1-F1 67.84(19), C1-W1-F2 91.6(2), C1-W1-F3 68.28(19), C1-W1-F4 88.4(2), C1-W1-F5 141.8(2), C1-W1-F6 141.7(2), F1-W1-F2 90.04(18), F1-W1-F3 135.72(18), F1-W1-F4 90.25(18), F1-W1-F5 73.97(19), F1-W1-F6 150.07(19), F2-W1-F3 85.53(18), F2-W1-F4 179.61(18), F2-W1-F5 88.2(2), F2-W1-F6 93.03(19), F3-W1-F4 94.17(18), F3-W1-F5 149.5(2), F3-W1-F6 74.20(19), F4-W1-F5 92.0(2), F4-W1-F6 86.79(19), F5-W1-F6 76.4(2).

C_20_H_31_F_6_NW, Mr = 583.31, T = 100.00(2) K, wavelength = 0.71073 Å, monoclinic space group P2_1_/n, a = 11.1336(2) Å, b = 9.9558(2) Å, c = 19.9089(3) Å, α = 90°, β = 99.252(2)°, γ = 90°, V = 2178.07(7) Å^3^, Z = 4, ρ(calcd) = 1.779 g/cm^3^ , µ = 5.358 mm^-1^ , F(000) = 1144, 53625 reflections in -13 ≤ h ≤ 13, -11 ≤ k ≤ 11, -23 ≤ l ≤ 23 measured in 1.973 < θ < 25.123°, completeness 100.0%, 3883 independent reflections, 3633 reflections observed in [I>2sigma(I)], 261 parameters, 0 restraints, R indices (all data) R1 = 0.0433, wR2 = 0.0950, final R indices [I>2sigma(I)] R1 = 0.0411, wR2 = 0.0939, largest difference peak and hole 7.026 and -1.014 eA^-3^, GooF = 1.085.

Crystal data collection of **[(I*i*Pr^Me^)WF_5_] 7**


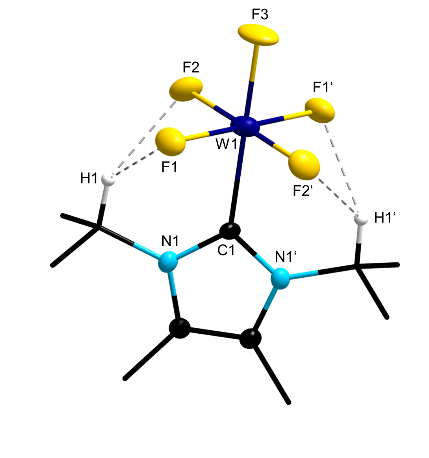


**Figure S36.** Molecular structure of [(IiPr^Me^)WF_6_] **7** in the solid state (ellipsoids set at the 50% probability level, carbon atoms except the ones of the carbene ring are shown as wire-and-stick models). Hydrogen atoms except the methine protons are omitted for clarity. Selected bond lengths [Å] and angles [°]: W1−C1 2.188(3), W1−F1 1.879(2), W1−F2 1.875(2), W1−F3 1.919(3), H1−F1 2.3020(21), H1−F2 2.3735(22), C1−N1 1.363(3); N1-C1-N1’ 105.6(3), N1-C1-W1 127.19(14), C1-W1-F1 90.19(5), C1-W1-F2 89.64(5), C1-W1-F3 180.000, F1-W1-F2 87.43(10), F1-W1-F3 89.81(5), F1-W1-F2’ 92.57(10), F2-W1-F3 90.36(5), F2-W1-F1’ 92.57(10).

C_11_H_20_F_5_N_2_W, Mr = 459.14, T = 100.00(2) K, wavelength = 0.71073 Å, monoclinic space group C2/c, a = 12.0547(4) Å, b = 10.7878(3) Å, c = 11.6213(4) Å, α = 90°, β = 106.168(4)°, γ = 90°, V = 1451.50(9) Å^3^, Z = 4, ρ(calcd) = 2.101 g/cm^3^ , µ = 8.002 mm^-1^ , F(000) = 876, 11893 reflections in -17 ≤ h ≤ 16, -15 ≤ k ≤ 15, -16 ≤ l ≤ 16 measured in 2.581 < θ < 30.505°, completeness 100.0%, 2225 independent reflections, 1995 reflections observed in [I>2sigma(I)], 91 parameters, 0 restraints, R indices (all data) R1 = 0.0278, wR2 = 0.0569, final R indices [I>2sigma(I)] R1 = 0.0223, wR2 = 0.0548, largest difference peak and hole 1.432 and -1.0007 eA^-3^, GooF = 1.022.

Crystal data collection of **[(BI*i*Pr)WF_5_]** **8**


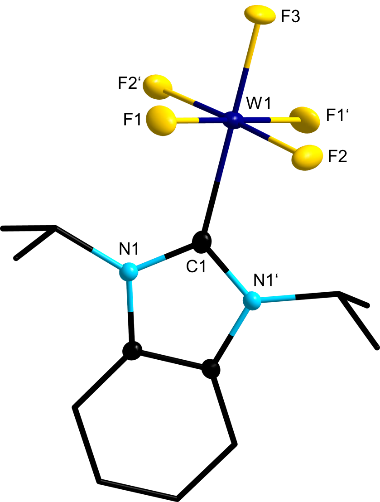


**Figure S37**. Molecular structure of [(BIiPr)WF_5_] **8** in the solid state (ellipsoids set at the 50% probability level, carbon atoms except the ones of the carbene ring are shown as wire-and-stick models). Hydrogen atoms are omitted for clarity. Selected bond lengths [Å] and angles [°]: W1−C1 2.213(5), W1−F1 1.869(2), W1−F2 1.869(2), W1−F3 1.913(3), C1−N1 1.358(4); N1-C1-N1’ 107.3(4), N1-C1-W1 126.3(2), C1-W1-F1 89.88(7), C1-W1-F2 88.74(7), C1-W1-F3 180.000, F1-W1-F2 88.01(12), F1-W1-F3 90.12(7), F2-W1-F1’ 91.99(12), F2-W1-F3, 91.26(7).

C_13_H_18_F_5_N_2_W, Mr = 481.14, T = 100.00(2) K, wavelength = 0.71073 Å, monoclinic space group C2/c, a = 11.4060(3) Å, b = 11.8904(3) Å, c = 11.5274(3) Å, α = 90°, β = 107.812(3)°, γ = 90°, V = 1488.43(7) Å^3^, Z = 4, ρ(calcd) = 2.147 g/cm^3^ , µ = 7.809 mm^-1^ , F(000) = 916, 1839 reflections in -15 ≤ h ≤ 14, -15 ≤ k ≤ 15, -10 ≤ l ≤ 15 measured in 2.540 < θ < 28.277°, completeness 100.0%, 1839 independent reflections, 1800 reflections observed in [I>2sigma(I)], 100 parameters, 1 restraints, R indices (all data) R1 = 0.0189, wR2 = 0.0530, final R indices [I>2sigma(I)] R1 = 0.0183, wR2 = 0.0527, largest difference peak and hole 2.095 and -1.254 eA^-3^, GooF = 1.187.

Crystal data collection of **[(IMes)WF_5_] 9**


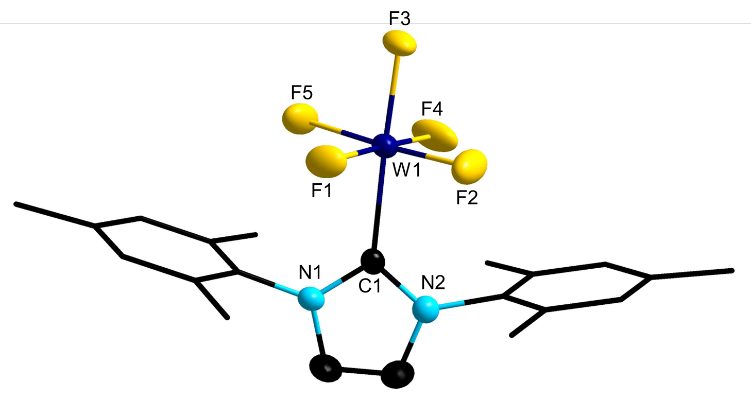


**Figure S38.** Molecular structure of [(IMes)WF_5_] **9** in the solid state (ellipsoids set at the 50% probability level, carbon atoms except the ones of the carbene ring are shown as wire-and-stick models). Hydrogen atoms are omitted for clarity. Selected bond lengths [Å] and angles [°]: W1−C1 2.215(6), W1−F1 1.854(4), W1−F2 1.866(4), W1−F3 1.928(4), W1−F4 1.841(5), W1−F5 1.870(4), C1−N1 1.348(8), C1−N2 1.358(8); N1-C1-N2 104.3(5), N1-C1-W1 127.6(4), N2-C1-W1 128.1(4), C1-W1-F1 89.5(2), C1-W1-F2 90.9(2), C1-W1-F3 176.96(19), C1-W1-F4 87.0(2), C1-W1-F5 93.53(19), F1-W1-F2 89.3(2), F1-W1-F3 93.48(18), F1-W1-F4 175.1(2), F1-W1-F5 87.3(2), F2-W1-F3 88.47(19), F2-W1-F4 94.1(3), F2-W1-F5 174.4(2), F3-W1-F4 90.11(19), F3-W1-F5 87.26(18), F4-W1-F5 89.5(2).

C_21_H_24_F_5_N_2_W, Mr = 583.27, T = 100.00(2) K, wavelength = 0.71073 Å, orthorhombic space group Pbca, a = 15.1413(6) Å, b = 15.9599(6) Å, c = 17.9081(6) Å, α = 90°, β = 90°, γ = 90°, V = 4327.6(3) Å^3^, Z = 8, ρ(calcd) = 1.790 g/cm^3^ , µ = 5.389 mm^-1^ , F(000) = 2264, 27295 reflections in -17 ≤ h ≤ 18, -19 ≤ k ≤ 18, -21 ≤ l ≤ 21 measured in 2.175 < θ < 25.120°, completeness 100.0%, 3855 independent reflections, 3220 reflections observed in [I>2sigma(I)], 268 parameters, 0 restraints, R indices (all data) R1 = 0.0496, wR2 = 0.1103, final R indices [I>2sigma(I)] R1 = 0.0401, wR2 = 0.1053, largest difference peak and hole 1.943 and -0.714 eA^-3^, GooF = 1.072.

Crystal data collection of **[(IDipp)WF_5_]** **10**


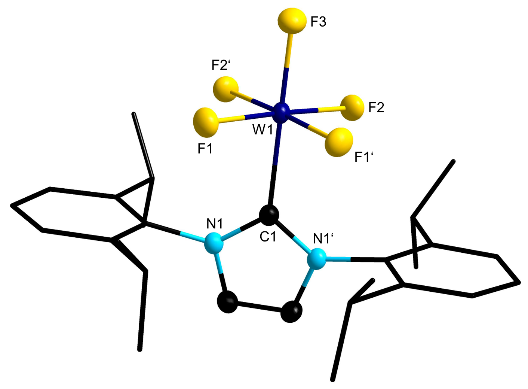


**Figure S39.** Molecular structure of [(IDipp)WF_5_] **10** in the solid state (ellipsoids set at the 50% probability level, carbon atoms except the ones of the carbene ring are shown as wire-and-stick models). Hydrogen atoms are omitted for clarity. Selected bond lengths [Å] and angles [°]: W1−C1 2.203(3), W1−F1 1.8614(15), W1−F2 1.8766(15), W1−F3 1.899(2), C1−N1 1.358(3); N1-C1-N1’ 104.6(3), N1-C1-W1 127.44(14), C1-W1-F1 90.17(8), C1-W1-F2 86.87(8), C1-W1-F3 176.80(12), F1-W1-F2 89.91(8), F1-W1-F3 92.08(8), F2-W1-F3 90.86(8), F1-W1-F1’ 90.62(11), F2-W1-2’ 89.4(1).

C_27_H_36_F_5_N_2_W, Mr = 667.43, T = 100.00(2) K, wavelength = 1.54184 Å, orthorhombic space group Pnma, a = 12.6840(2) Å, b = 19.5718(3) Å, c = 11.00080(10) Å, α = 90°, β = 90°, γ = 90°, V = 2730.93(6) Å^3^, Z = 4, ρ(calcd) = 1.623 g/cm^3^ , µ = 8.288 mm^-1^ , F(000) = 1324, 15632 reflections in -16 ≤ h ≤ 15, -24 ≤ k ≤ 24, -12 ≤ l ≤ 13 measured in 4.518 < θ < 77.580°, completeness 98.6%, 2950 independent reflections, 2655 reflections observed in [I>2sigma(I)], 167 parameters, 0 restraints, R indices (all data) R1 = 0.0270, wR2 = 0.0730, final R indices [I>2sigma(I)] R1 = 0.0249, wR2 = 0.0713, largest difference peak and hole 0.974 and -1.049 eA^-3^, GooF = 1.115.

Crystal data collection of **[(cAAC^Me^)WF_5_]_2_** **12**


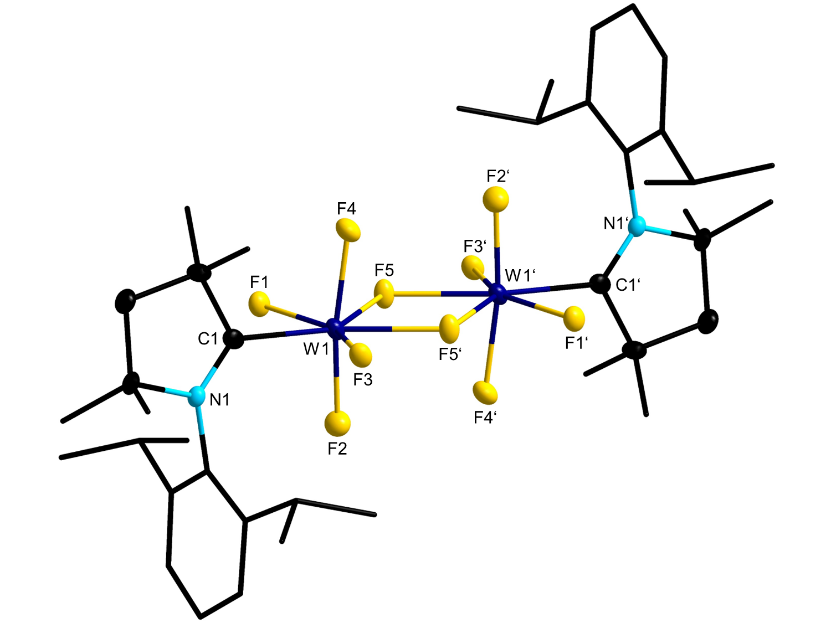


**Figure S40.** Molecular structure of [(cAAC^Me^)WF_5_]_2_ **12** in the solid state (ellipsoids set at the 50% probability level, carbon atoms except the ones of the carbene rings are shown as wire-and-stick models). Hydrogen atoms are omitted for clarity. Selected bond lengths [Å] and angles [°]: W1−W1’ 3.5117(4), W1−C1 2.209(5), W1−F1 1.883(3), W1−F2 1.834(3), W1−F3 1.885(3), W1−F4 1.904(3), W1−F5 2.093(3), W1−F5’ 2.104(3), F5−F5’ 2.2996(39), C1−N1 1.292(6); N1-C1-W1 129.0(3), C1-W1-F1 72.98(15), C1-W1-F2 95.81(15), C1-W1-F3 72.88(14), C1-W1-F4 93.10(15), C1-W1-F5 146.18(14), C1-W1-F5’ 146.37(14), F1-W1-F2 93.21(13), F1-W1-F3 145.79(12), F1-W1-F4 90.89(13), F1-W1-F5 73.62(12), F1-W1-F5’ 140.02(11), F2-W1-F3 92.44(13), F2-W1-F4 170.95(13), F2-W1-F5 91.20(13), F2-W1-F5’ 89.41(13), F3-W1-F4 88.69(13), F3-W1-F5 139.94(11), F3-W1-F5’ 73.72(11), F4-W1-F5 82.21(3) , F4-W1-F5’ 82.28(12), F5-W1-F5’ 66.44(13), W1-F5-W1’ 113.56(13).

C_40_H_62_F_10_N_2_W_2_, Mr = 1128.61, T = 100.00(2) K, wavelength = 0.71073 Å, monoclinic space group P2_1_/n, a = 8.3788(2) Å, b = 15.2553(3) Å, c = 15.6050(3) Å, α = 90°, β = 95.943(2)°, γ = 90°, V = 1983.93(7) Å^3^, Z = 2, ρ(calcd) = 1.889 g/cm^3^ , µ = 5.872 mm^-1^ , F(000) = 1108, 47434 reflections in -10 ≤ h ≤ 10, -18 ≤ k ≤ 18, -18 ≤ l ≤ 18 measured in 2.625 < θ < 25.123°, completeness 100.0%, 3532 independent reflections, 3190 reflections observed in [I>2sigma(I)], 252 parameters, 0 restraints, R indices (all data) R1 = 0.0326, wR2 = 0.0737, final R indices [I>2sigma(I)] R1 = 0.0284, wR2 = 0.0712, largest difference peak and hole 3.403 and -1.234 eA^-3^, GooF = 1.016.

Crystal data collection of **[(cAAC^Me^)_2_WF_5_]** **13**


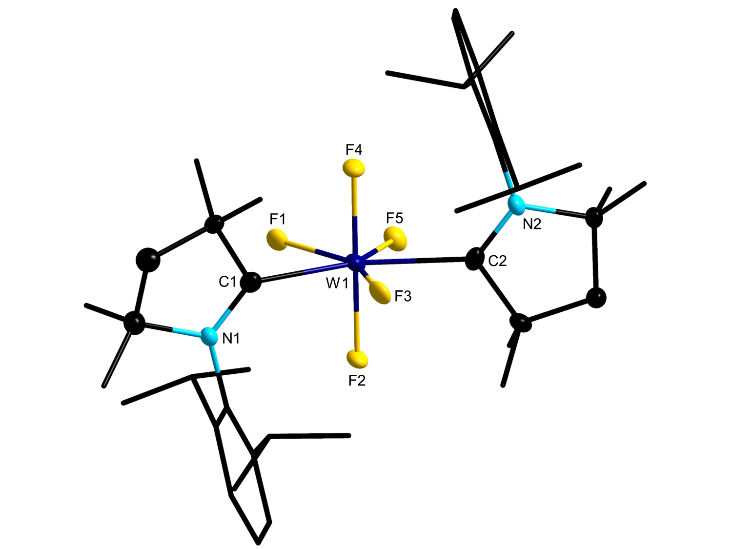


**Figure S41.** Molecular structure of [(cAAC^Me^)_2_WF_5_] **13** in the solid state (ellipsoids set at the 50% probability level, carbon atoms except the ones of the carbene rings are shown as wire-and-stick models). Hydrogen atoms are omitted for clarity. Selected bond lengths [Å] and angles [°]: W1−C1 2.289(5), W1−C2 2.277(5), W1−F1 1.924(3), W1−F2 1.915(3), W1−F3 1.915(3), W1−F4 1.909(3), W1−F5 1.948(3), C1−N1 1.306(6), C2−N2 1.311(7); N1-C1-W1 129.2(4), N2-C2-W1 129.5(4), C1-W1-C2 148.95(18), C1-W1-F1 68.68(14), C1-W1-F2 89.81(5), C1-W1-F3 74.70(15), C1-W1-F4 89.23(15), C1-W1-F5 142.22(15), C2-W1-F1 142.09(15), C2-W1-F2 91.44(15), C2-W1-F3 74.31(15), C2-W1-F4 88.35(16), C2-W1-F5 68.81(15), F1-W1-F2 93.56(11), F1-W1-F3 143.27(11), F1-W1-F4 87.94(2), F1-W1-F5 73.89(12), F2-W1-F3 88.99(13), F2-W1-F4 177.79(12), F2-W1-F5 87.59(3), F3-W1-F4 88.84(12), F3-W1-F5 142.85(12), F4-W1-F5 94.38(13).

C_40_H_62_F_5_N_2_W, Mr = 849.76, T = 100.00(2) K, wavelength = 1.54184 Å, monoclinic space group P2_1_/n, a = 19.0805(3) Å, b = 9.78530(10) Å, c = 21.1416(4) Å, α = 90°, β = 100.841(2)°, γ = 90°, V = 3876.87(11) Å^3^, Z = 4, ρ(calcd) = 1.456 g/cm^3^ , µ = 5.961 mm^-1^ , F(000) = 1740, 25546 reflections in -22 ≤ h ≤ 22, -11 ≤ k ≤ 11, -25 ≤ l ≤ 23 measured in 2.864 < θ < 67.078°, completeness 98.8%, 6853 independent reflections, 5809 reflections observed in [I>2sigma(I)], 449 parameters, 0 restraints, R indices (all data) R1 = 0.0548, wR2 = 0.1251, final R indices [I>2sigma(I)] R1 = 0.0457, wR2 = 0.1195, largest difference peak and hole 1.828 and -1.467 eA^-3^, GooF = 1.090.

1. **Additional Tables and Figures**

**Table S1.** Selected experimental and calculated (DFT/PBE0-D3(BJ)/def2-TZVP) vibrational data (W−F) for the compounds **1**−**11** (DFT calculated values are uncorrected).

| **compound** | **IR** (exp.) [cm^-1^] | **Raman** (exp.) [cm^-1^] | **calcd.** [cm^-1^] |
| --- | --- | --- | --- |
| **1** | 685, 707 | 686 |  |
| **2** | 658, 689 | 690 | 662, 704 |
| **3** | 668, 718 | 708 | 669, 712 |
| **4** | 670, 708 | 710 | 667, 710 |
| **5** | 674, 710 | 711 |  |
| **6** | 666, 704 | 705 |  |
| **7** | 675, 734 | 673 |  |
| **8** | 685, 716 | 687 |  |
| **9** | 692, 711 | 691 |  |
| **10** | 678, 710 | 693 |  |
| **11** | 676, 709 | 683 |  |

**Table S2.** Selected bond lengths [Å] and angles [°] of the complexes **7**, **8**, **9**, and **10**.

| **compound** | W1−C1 [Å] | W−F [Å] | C1-W1-F3 [°] |
| --- | --- | --- | --- |
| [(I*i*Pr^Me^)WF_5_] **7** | 2.188(3) | W1−F1 1.879(2)  W1−F2 1.875(2)  W1−F3 1.919(3) | 180.000 |
| [(BI*i*Pr)WF_5_] **8** | 2.213(5) | W1−F1 1.869(2)  W1−F2 1.869(2)  W1−F3 1.913(3) | 180.000 |
| [(IMes)WF_5_] **9** | 2.215(6) | W1−F1 1.854(4)  W1−F2 1.866(4)  W1−F3 1.928(4)  W1−F4 1.841(5)  W1−F5 1.870(4) | 176.96(19) |
| [(IDipp)WF_5_] **10** | 2.203(3) | W1−F1 1.8614(15)  W1−F2 1.8766(15)  W1−F3 1.899(2) | 176.80(12) |


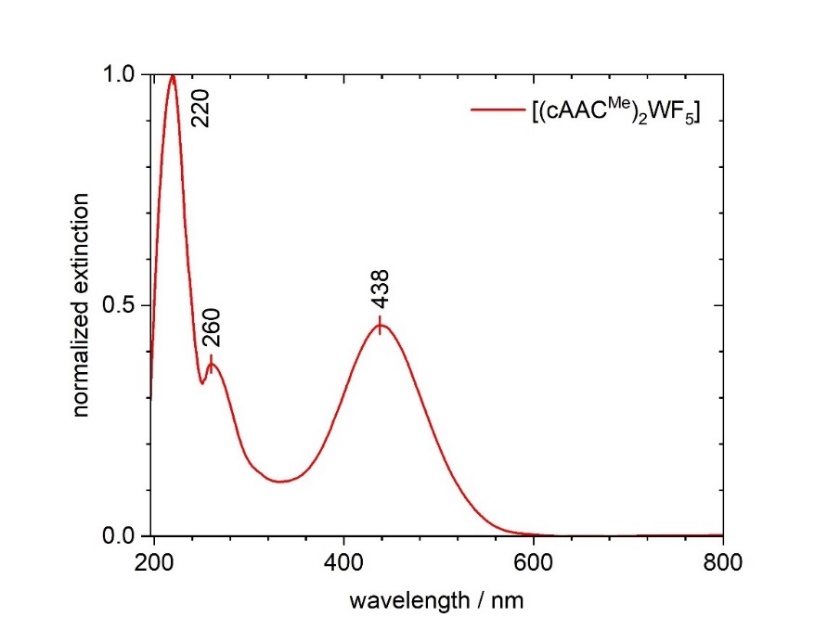


**Figure S42.** UV/Vis spectrum of **13** in n-hexane.

1. **Thermal analyses**


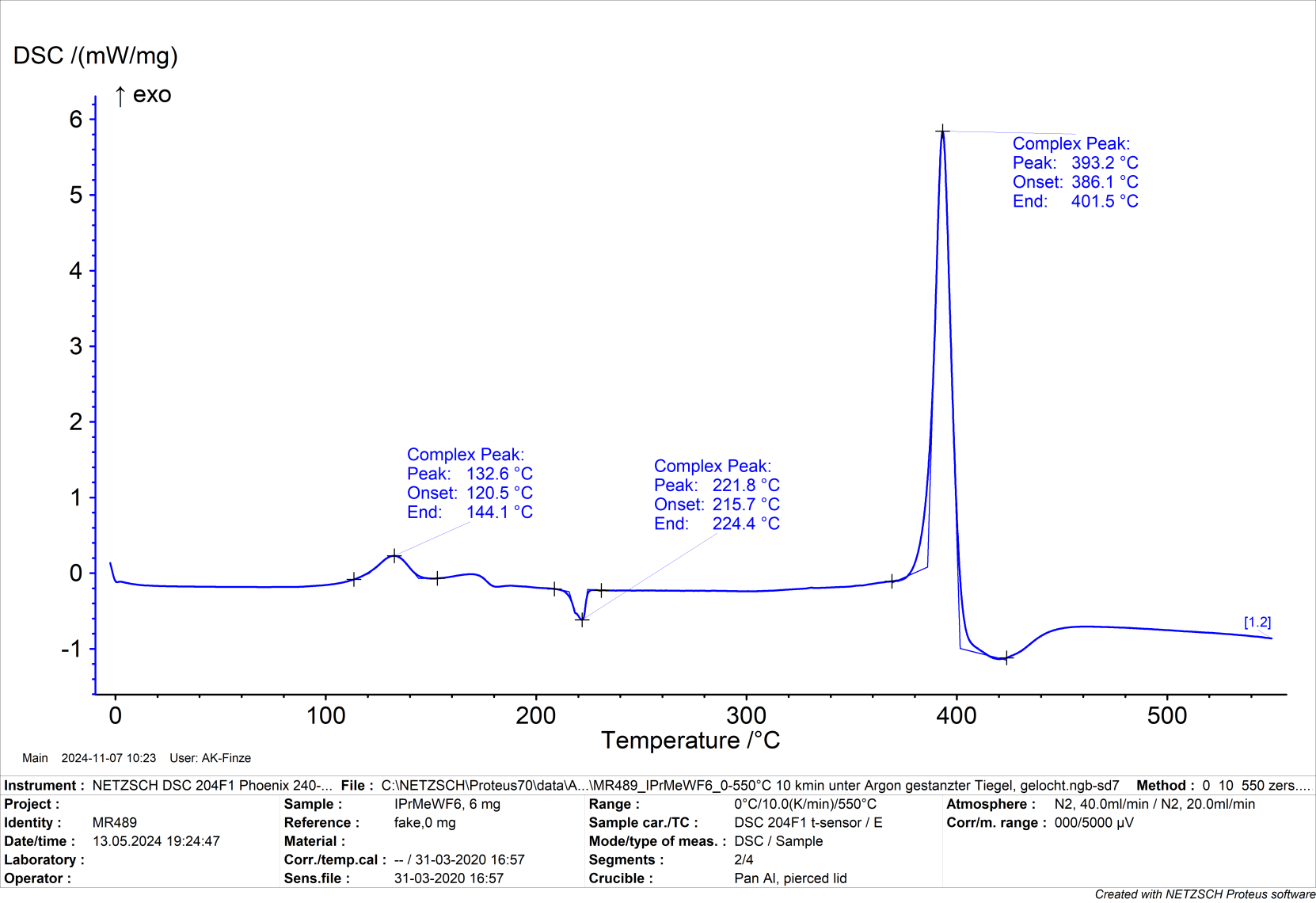


**Figure S43.** DSC curve of [(IiPr^Me^)WF_6_] **1.**


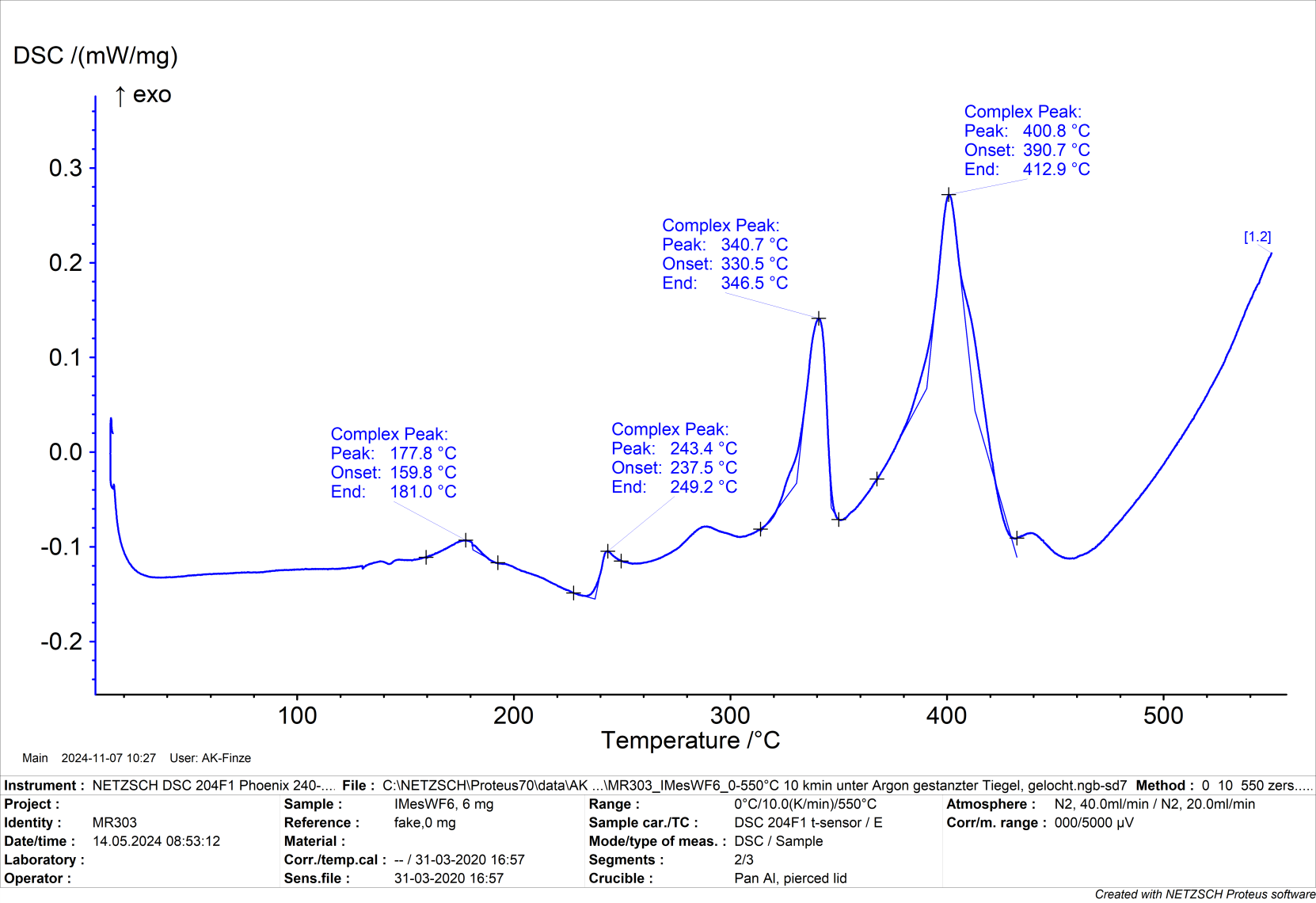


**Figure S44.** DSC curve of [(IMes)WF_6_] **3.**


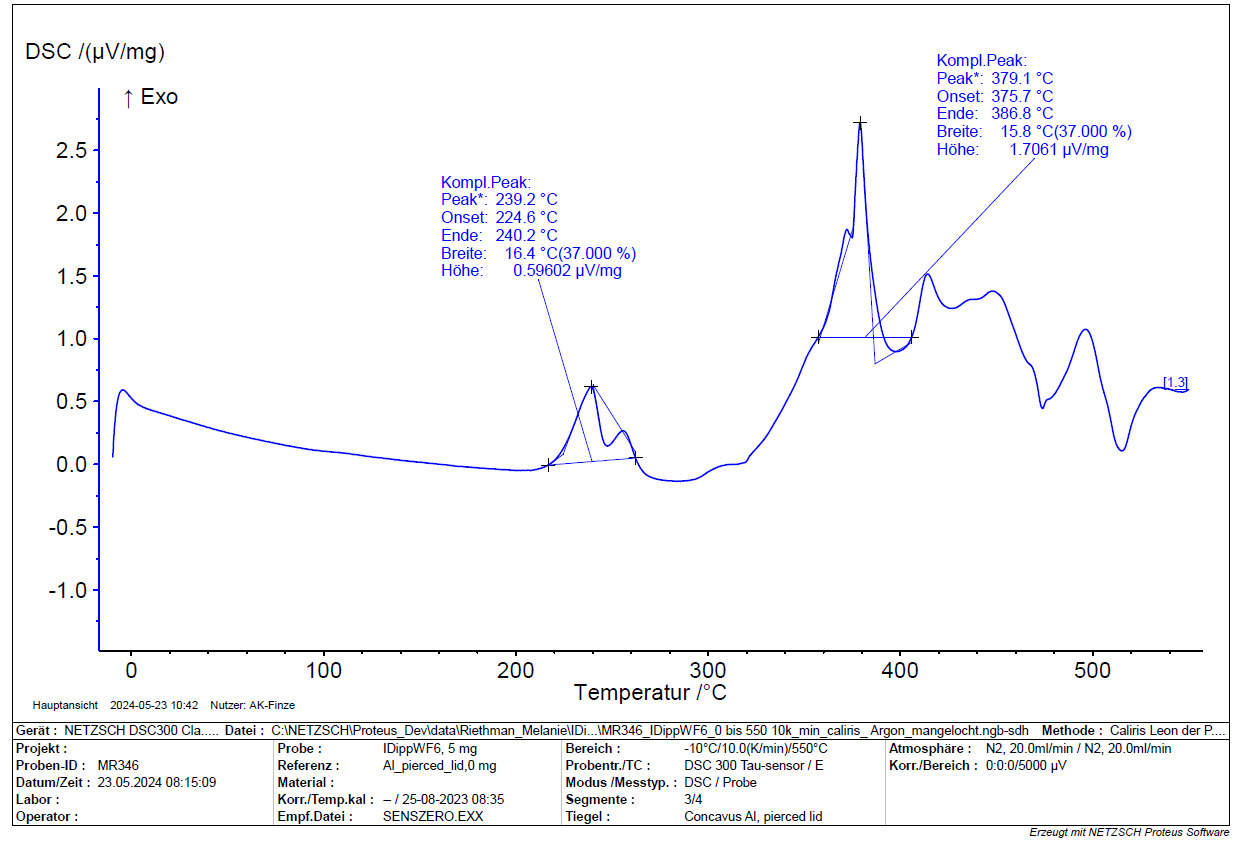


**Figure** **S45.** DSC curve of [(IDipp)WF_6_] **4.**


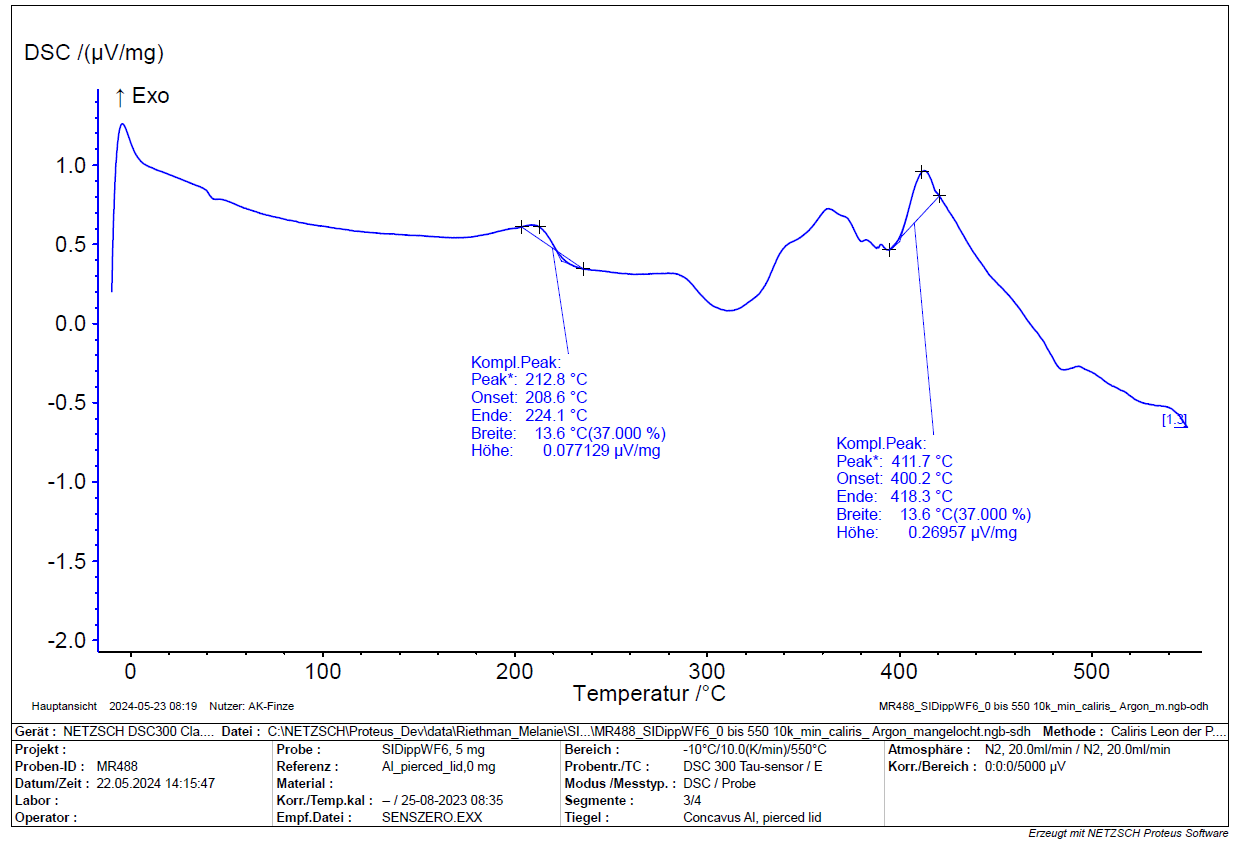


**Figure** **S46.** DSC curve of [(SIDipp)WF_6_] **5.**


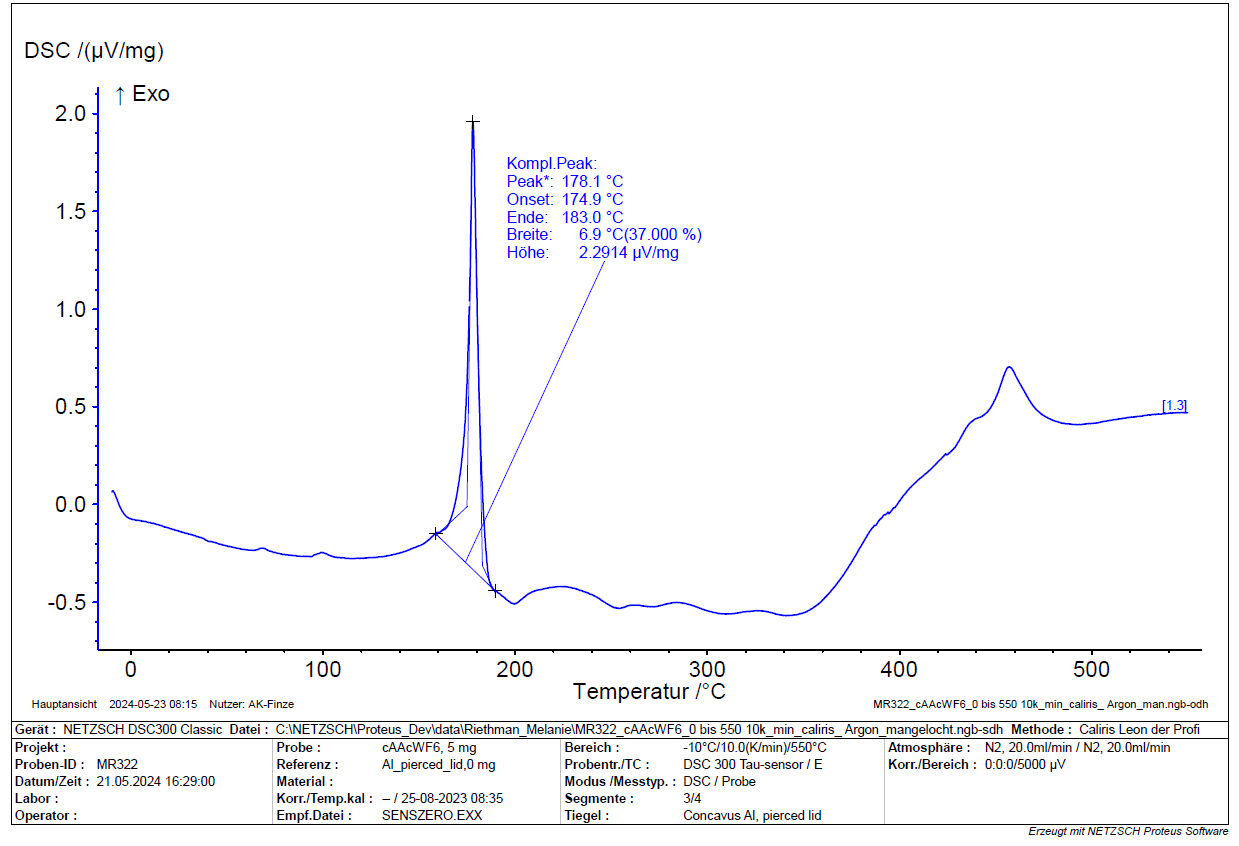


**Figure S47.** DSC curve of [(cAAC^Me^)WF_6_] **6.**

1. **Computational Details − Optimized Geometries**

Calculations were carried out using the TURBOMOLE V7.8.1 program suite, a development of the University of Karlsruhe and the Forschungszentrum Karlsruhe GmbH, 1989-2007, TURBOMOLE GmbH, since 2007; available from https://www.turbomole.org.^[16, 17]^ Geometry optimizations were performed using (RI-)DFT calculations^[18-20]^ on a m4 grid employing the BPE0^[21, 22]^ functional and a def2-TZVP basis set for all atoms.^[23-26]^ Dispersion corrections were considered in the geometry optimizations by using Grimme’s D3^[27]^ correction together with the Becke-Johnson (BJ) damping function (level denoted as PBE0-D3(BJ)/def2TZVP).^[28]^ All stationary points were fully characterized by analytical frequency calculations as either minima (only positive eigenvalues) or transition states (one negative eigenvalue), if not stated otherwise. Vibrational frequencies were calculated at the same level with the AOFORCE^[29]^ module and all structures represented true minima without imaginary frequencies. Natural population analysis,^[30]^ NBO^[31]^ and Wiberg bond indices^[32]^ have been evaluated from the DFT ground state electron density. Vertical excitation energies were computed at the TD-DFT level^[33-35]^ using the long range corrected *ω*B97X-D^[36]^ functional and a def2-TZVP basis set (denoted as *ω*B97X/def2-TZVP).


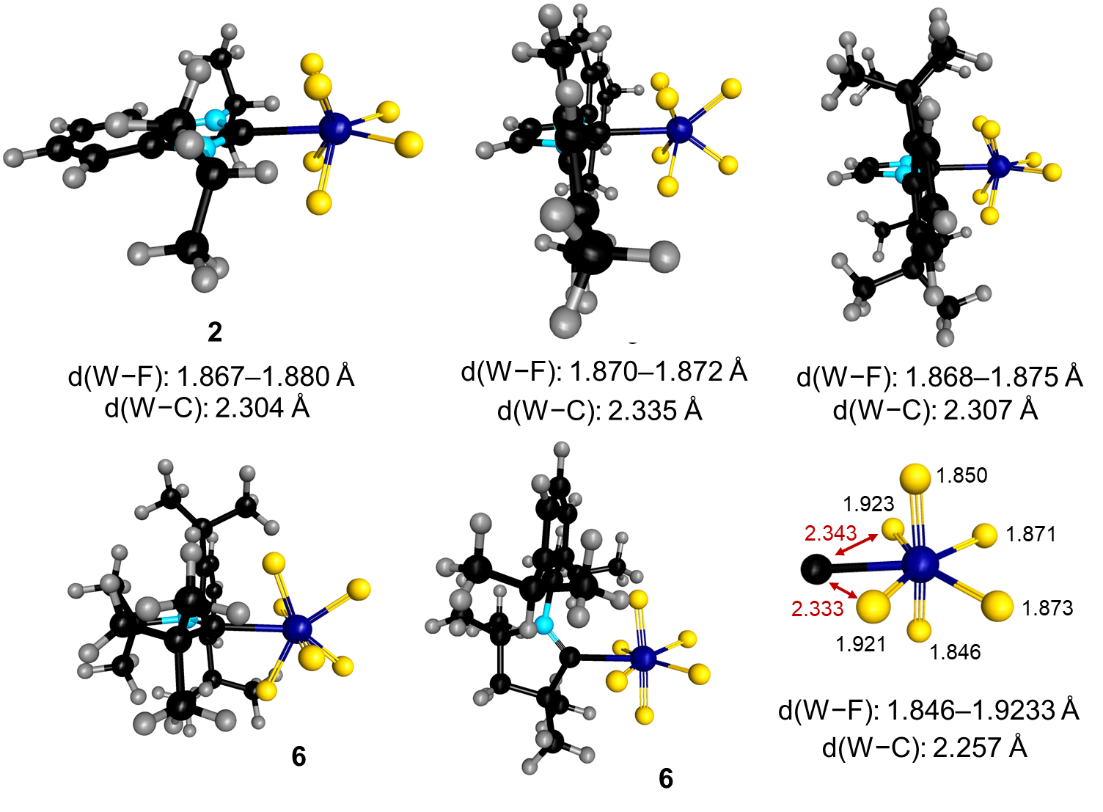


**Figure S48.** DFT-optimized geometries (PBE0-D3(BJ)/def2-TZVP level of theory) of [(BIiPr)WF_6_] **2**, [(IMes)WF_6_] **3**, [(IDipp)WF_6_] **4** (top), and [(cAAC^Me^)WF_6_] **6** (bottom) and calculated W−C and W−F bond lengths. The bottom row shows the DFT-optimized geometry of **6** in two different views and a truncated picture thereof with W−F bond lengths for each of the fluoride ligands.


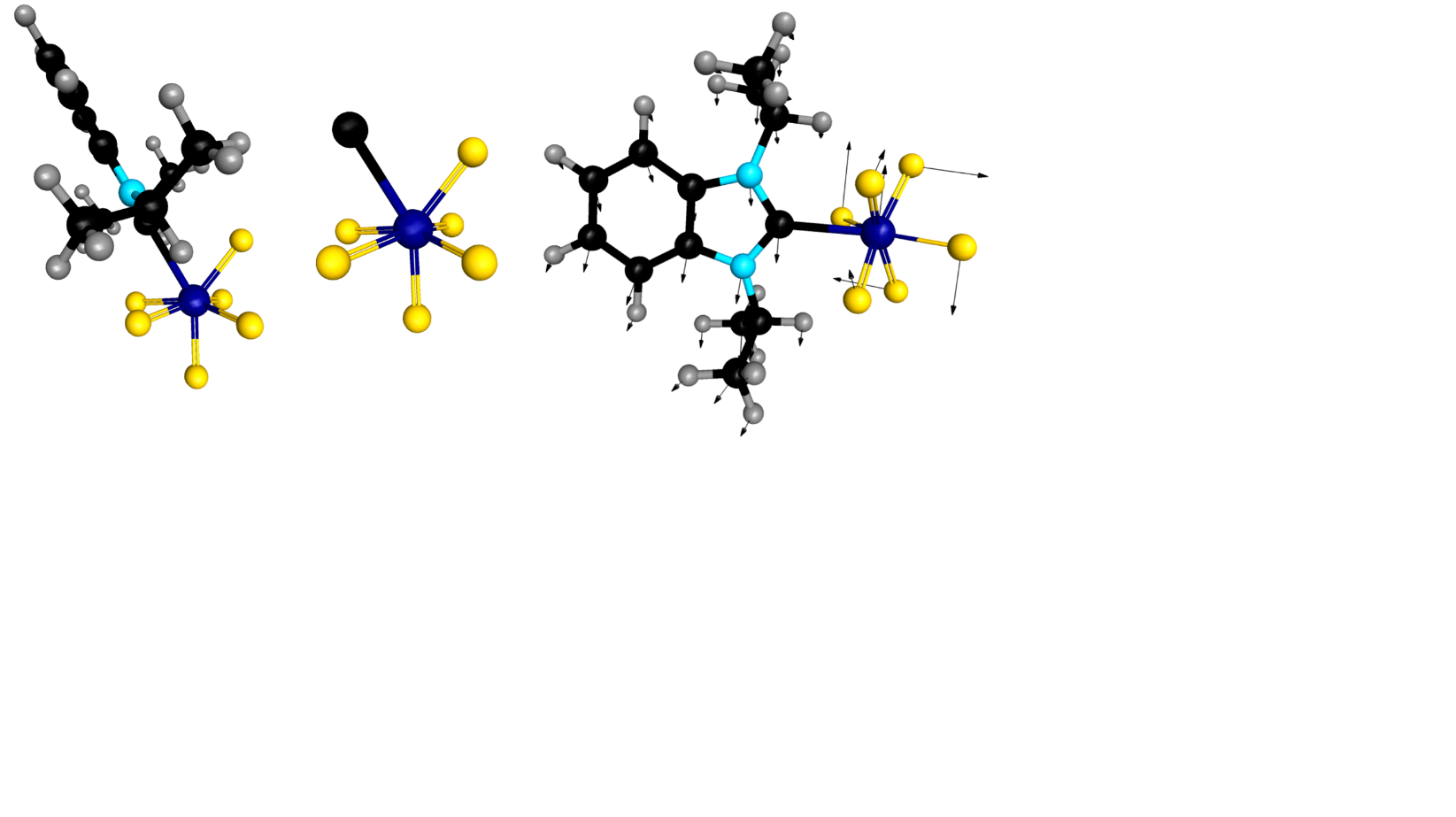


**Figure S49.** Transition state for rapid fluoride exchange in [(BIiPr)WF_6_] **2**: Visualization of the capped octahedral geometry (left, middle) and the vibrational mode of the imaginary frequency at i46.9 cm^-1^.


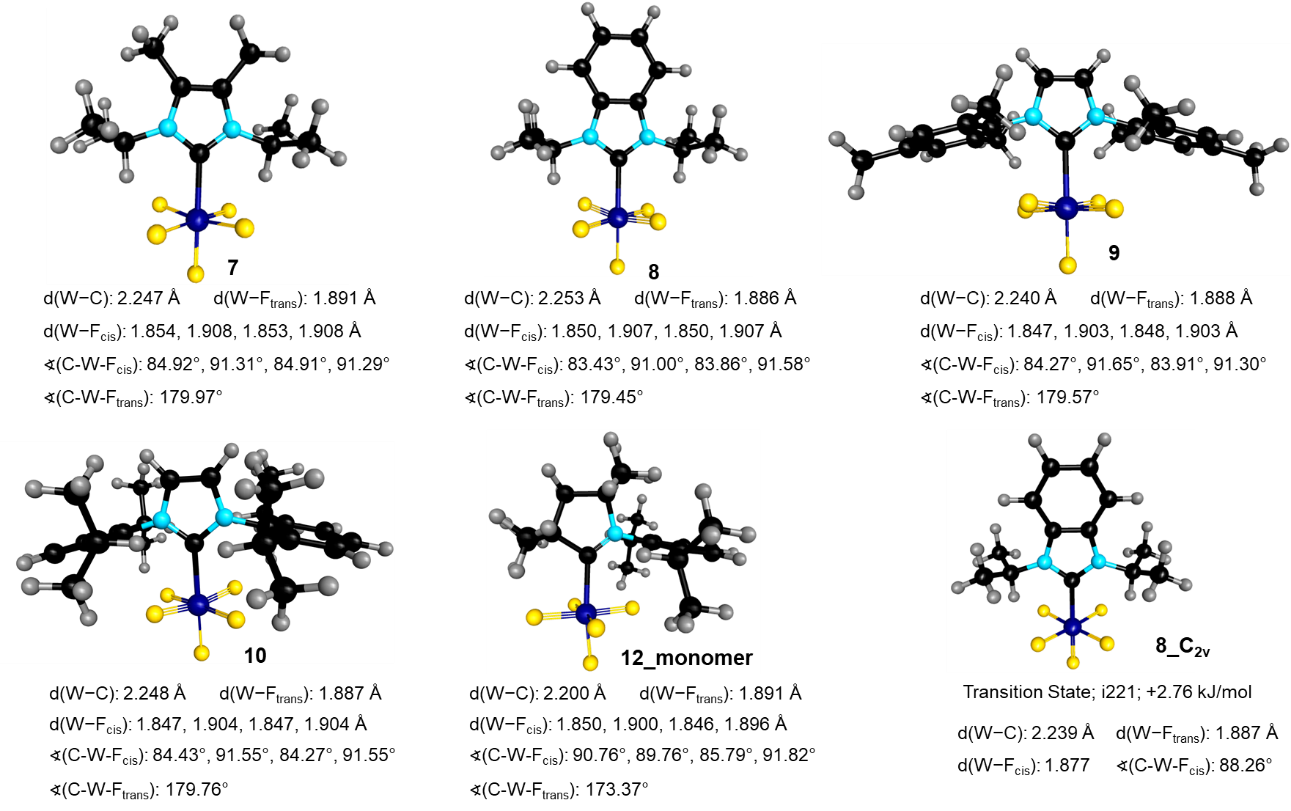


**Figure S50**. DFT-optimized geometries (PBE0-D3(BJ)/def2-TZVP level of theory) of [(IiPr^Me^)WF_5_] **7**, [(BIiPr)WF_5_] **8**, [(IMes)WF_5_]**9**, [(IDipp)WF_5_] **10**, the monomer of [(cAAC^Me^)WF_5_]_2_ **12** (**12_monomer**) and the geometry of **8** optimized with C_2v_-symmetry restraints (**8_C_2v_**), and DFT-calculated W−C and W−F bond lengths and C-W-F bond angles. **8_C_2v_** is a transition state at the energy hypersurface showing one imaginary frequency.


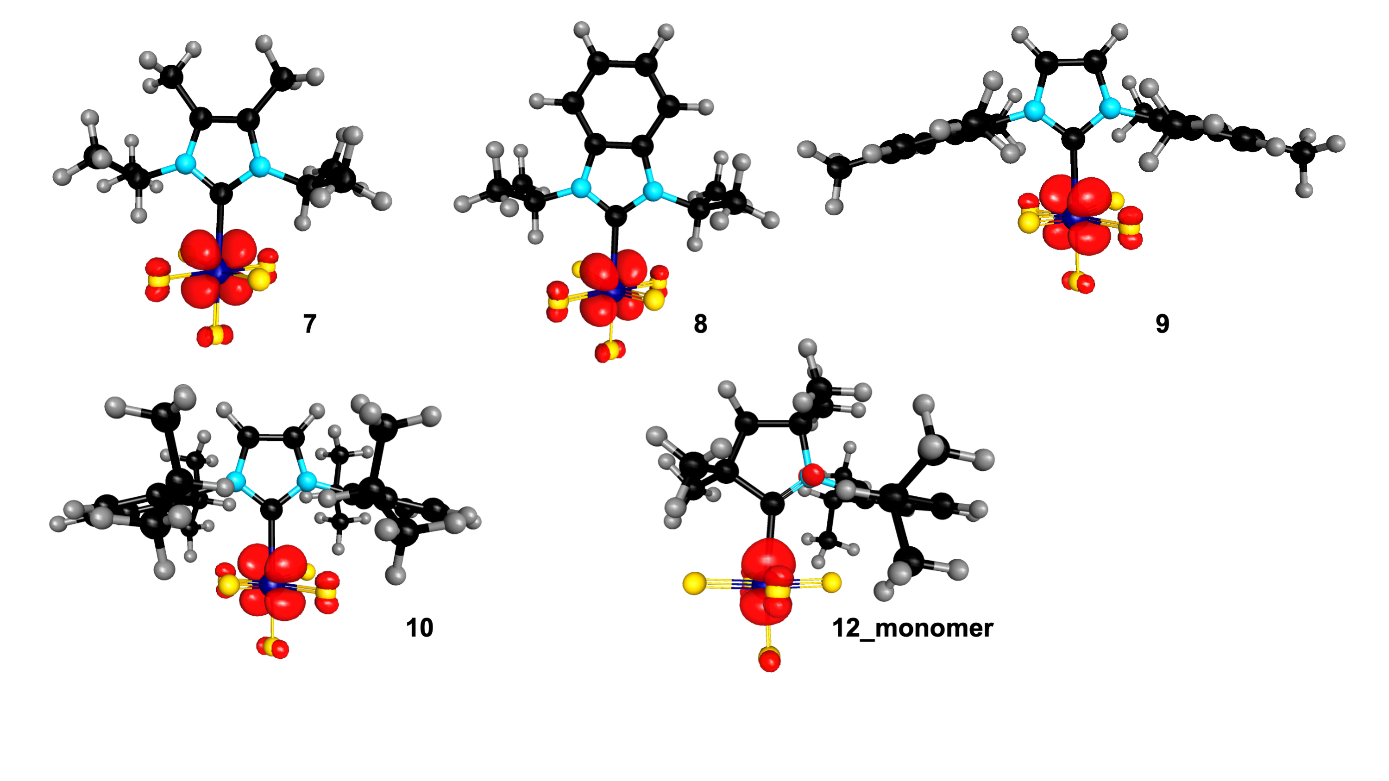


**Figure S51.** Spin density calculated for the monomer complexes **7**–**12** (PBE0-D3(BJ)/def2-TZVP level of theory).


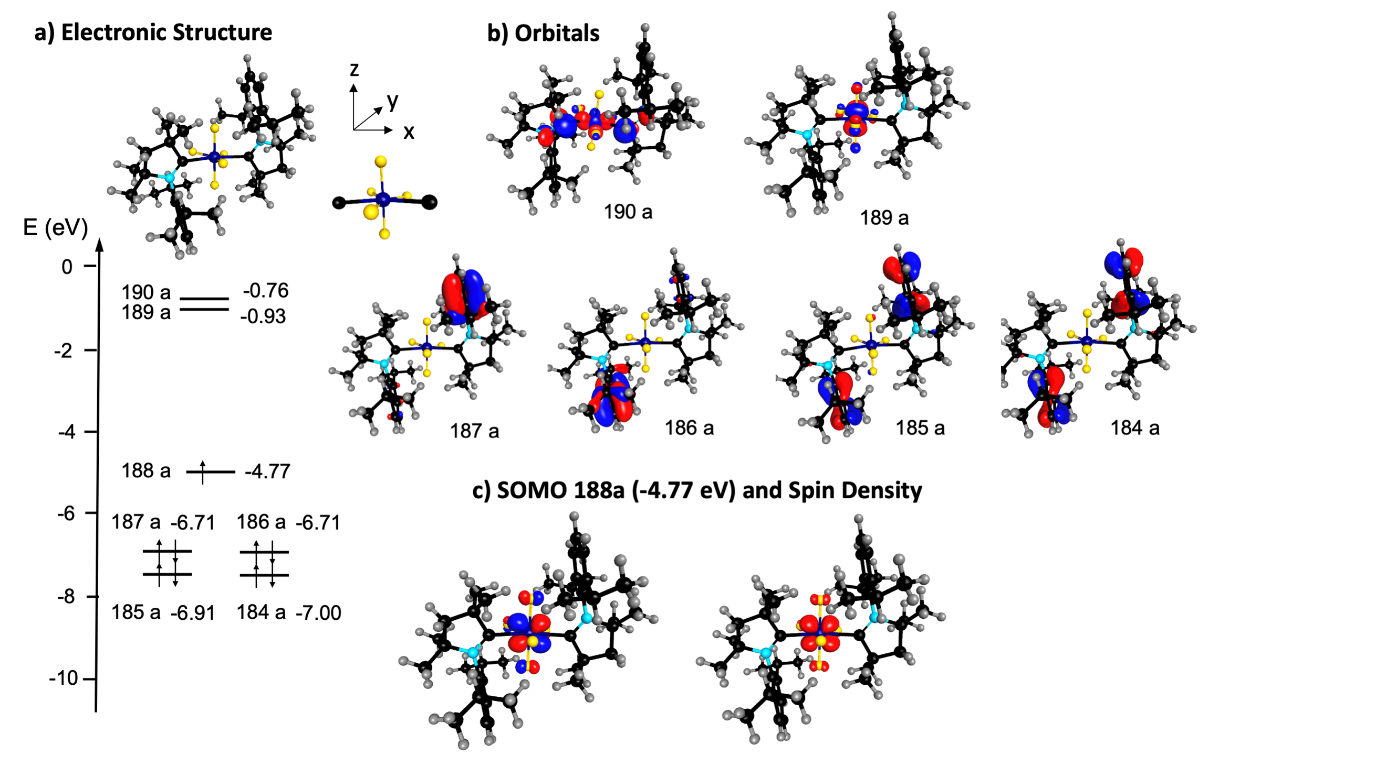


**Figure S52.** Electronic structure, important orbitals, SOMO and spin density of [(cAAC^Me^)_2_WF_5_] **13**.


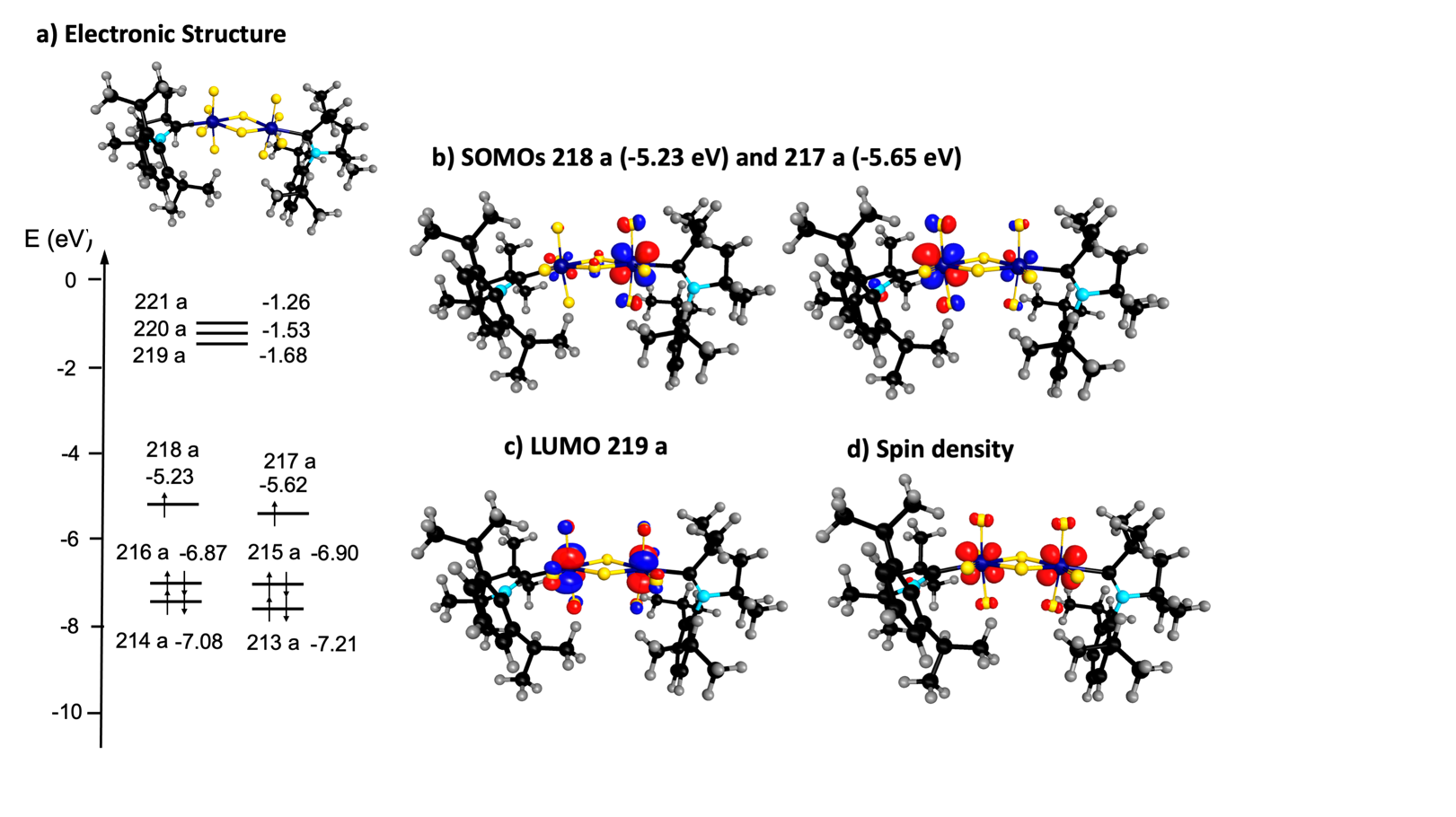


**Figure S53.** Electronic structure, important orbitals, SOMO and spin density of [(cAAC^Me^)WF_5_]_2_ **12**.

**Cartesian Coordinates of Geometry-optimized Molecules**

[(I*i*Pr^Me^)WF_6_] **1**

Energy = -1206.443804288

W -0.9508613 -0.0438183 -0.0058673

C -0.9571864 2.3024296 -0.0044369

N -0.8775883 3.1240113 -1.0778620

N -1.0395354 3.1224425 1.0699908

C -0.8233385 2.6997920 -2.4905458

C -0.9094651 4.4489269 -0.6791986

C -1.0116035 4.4479391 0.6729690

C -1.0947211 2.6963066 2.4820565

H -0.6944229 1.6302729 -2.4577051

C -2.1405542 2.9671078 -3.1991095

C 0.3939556 3.2546257 -3.2112560

C -0.8167952 5.6366352 -1.5664218

C -1.1073719 5.6343211 1.5616653

H -1.2204647 1.6264433 2.4476421

C -2.3150115 3.2467247 3.2011334

C 0.2202746 2.9666706 3.1935742

H -2.9623885 2.5037026 -2.6533724

H -2.1025156 2.5183265 -4.1935732

H -2.3565806 4.0285015 -3.3253277

H 0.3118148 4.3127148 -3.4560588

H 0.5128857 2.7105691 -4.1499355

H 1.2966550 3.0953424 -2.6192151

H -1.4247646 5.5400382 -2.4634762

H -0.5008661 5.5372517 2.4596275

H -2.4338823 2.7016139 4.1392089

H -3.2161859 3.0851161 2.6073993

H -2.2366036 4.3048918 3.4468333

H 0.4324503 4.0285129 3.3224962

H 1.0447380 2.5071856 2.6485164

H 0.1818124 2.5156959 4.1870312

F 0.1557020 0.4131097 1.4394923

F -2.0608094 0.4082777 -1.4502253

F -2.2847215 0.3979362 1.2385922

F 0.3797949 0.4073605 -1.2503335

F -2.0940975 -1.5196385 -0.1118368

F 0.2017666 -1.5125223 0.0984036

H -1.1730170 6.5151075 -1.0302731

H 0.2112150 5.8328546 -1.8800411

H -0.7513285 6.5140140 1.0273852

H -2.1361842 5.8288073 1.8737540

[(BI*i*Pr)WF_6_] **2**, capped trigonal prismatic conformer 1

Energy = -1281.409825148

W -1.0010052 0.0042034 0.0870704

C -0.9693618 2.3071733 0.0190735

N -0.9523112 3.0742452 -1.0897481

N -0.9660455 3.1340709 1.0844340

C -0.9473557 2.6029562 -2.4848305

C -0.9386109 4.4148806 -0.7358060

C -0.9469710 4.4531302 0.6576376

C -0.9811088 2.7406059 2.5033893

H -0.9597569 1.5229997 -2.4291411

C -2.2149690 3.0316566 -3.2036417

C 0.3399343 3.0028303 -3.1849074

C -0.9200503 5.5855621 -1.4871427

C -0.9369248 5.6631222 1.3440096

H -0.9961613 1.6592448 2.5079163

C -2.2569060 3.2107370 3.1806548

C 0.2978333 3.1758974 3.1976236

H -3.0925077 2.6724156 -2.6649370

H -2.2238501 2.5861050 -4.1996887

H -2.2961836 4.1125814 -3.3199777

H 0.4477445 4.0816225 -3.2994253

H 0.3528377 2.5574429 -4.1809863

H 1.2011175 2.6232473 -2.6338607

H -0.9131610 5.5739614 -2.5664250

C -0.9105257 6.7845693 -0.8017404

H -0.9430180 5.7105452 2.4223254

C -0.9189205 6.8228276 0.5942162

H -2.2805305 2.8192451 4.1989544

H -3.1282708 2.8252605 2.6501786

H -2.3360580 4.2964971 3.2378285

H 0.4067812 4.2591704 3.2537323

H 1.1652461 2.7648647 2.6799858

H 0.2963301 2.7862603 4.2168973

H -0.8961702 7.7124384 -1.3600790

H -0.9114502 7.7798467 1.1011123

F -1.0385301 -1.4087594 1.3061149

F -2.3488427 0.4488079 -1.1458115

F -2.3801124 0.5326289 1.2513531

F 0.3689587 0.4886880 1.2797560

F -1.0049568 -1.4934924 -1.0265481

F 0.3823430 0.4175107 -1.1182665

[(BI*i*Pr)WF_6_] **2**, capped trigonal prismatic conformer 2

Energy = -1281.404431003

W -0.0002889 0.0009483 -3.2455848

C 0.0000955 -0.0004772 -0.8987044

N 1.0873982 -0.0004879 -0.0926748

N -1.0872073 -0.0006755 -0.0926927

C 2.5060884 -0.0005806 -0.4943499

C 0.6960658 -0.0001379 1.2365650

C -0.6959303 -0.0004329 1.2365541

C -2.5058596 -0.0006933 -0.4945079

H 2.5071060 -0.0006206 -1.5727943

C 3.1932293 1.2787903 -0.0478377

C 3.1929885 -1.2800414 -0.0477417

C 1.4151821 0.0006629 2.4288126

C -1.4150808 -0.0002276 2.4287834

H -2.5066944 -0.0011682 -1.5729598

C -3.1928670 1.2789897 -0.0486888

C -3.1930673 -1.2798761 -0.0475722

H 2.6701184 2.1460738 -0.4528072

H 4.2115435 1.2864276 -0.4402429

H 3.2517876 1.3848220 1.0352961

H 3.2508849 -1.3863338 1.0354150

H 4.2115375 -1.2875691 -0.4395382

H 2.6701243 -2.1472331 -0.4532316

H 2.4940073 0.0013124 2.4484745

C 0.6984221 0.0011305 3.6084837

H -2.4939106 -0.0006362 2.4484219

C -0.6983463 0.0008031 3.6084699

H -4.2111248 1.2865991 -0.4412415

H -2.6695640 2.1460012 -0.4539951

H -3.2515584 1.3855420 1.0343893

H -3.2512720 -1.3857125 1.0356131

H -2.6702164 -2.1473107 -0.4525617

H -4.2115117 -1.2874192 -0.4396409

H 1.2316813 0.0015682 4.5511238

H -1.2316279 0.0014362 4.5510983

F -0.0008714 1.1554730 -4.7138556

F 1.3444509 -1.2234124 -2.7845438

F 1.3442936 1.2248510 -2.7827962

F -1.3448044 1.2246099 -2.7820134

F -0.0006530 -1.1513693 -4.7155849

F -1.3445487 -1.2236258 -2.7837385

[(BI*i*Pr)WF_6_] **2**, pentagonal bipyramidal conformer 3

Energy = -1281.391225681

W 0.0000000 0.0000000 -3.2569268

C 0.0000000 0.0000000 -0.8794341

N 0.0000000 -1.0852795 -0.0709568

N 0.0000000 1.0852795 -0.0709568

C 0.0000000 -2.4980311 -0.4710846

C 0.0000000 -0.6948582 1.2610118

C 0.0000000 0.6948582 1.2610118

C 0.0000000 2.4980311 -0.4710846

H 0.0000000 -2.4962449 -1.5495123

C 1.2792158 -3.1915548 -0.0310452

C -1.2792158 -3.1915548 -0.0310452

C 0.0000000 -1.4147372 2.4511940

C 0.0000000 1.4147372 2.4511940

H 0.0000000 2.4962449 -1.5495123

C 1.2792158 3.1915548 -0.0310452

C -1.2792158 3.1915548 -0.0310452

H 2.1500574 -2.6571349 -0.4142483

H 1.2936295 -4.2013938 -0.4443469

H 1.3751988 -3.2720668 1.0515630

H -1.3751988 -3.2720668 1.0515630

H -1.2936295 -4.2013938 -0.4443469

H -2.1500574 -2.6571349 -0.4142483

H 0.0000000 -2.4936175 2.4707572

C 0.0000000 -0.6977951 3.6320947

H 0.0000000 2.4936175 2.4707572

C 0.0000000 0.6977951 3.6320947

H 1.2936295 4.2013938 -0.4443469

H 2.1500574 2.6571349 -0.4142483

H 1.3751988 3.2720668 1.0515630

H -1.3751988 3.2720668 1.0515630

H -2.1500574 2.6571349 -0.4142483

H -1.2936295 4.2013938 -0.4443469

H 0.0000000 -1.2315164 4.5744174

H 0.0000000 1.2315164 4.5744174

F 0.0000000 1.8472946 -3.2918274

F 0.0000000 -1.8472946 -3.2918274

F 1.7626315 0.0000000 -2.5291006

F -1.7626315 0.0000000 -2.5291006

F 1.1524881 0.0000000 -4.7298879

F -1.1524881 0.0000000 -4.7298879

[(BI*i*Pr)WF_6_] **2**, capped octahedral transition state

i 46.9 cm^-1^

Energy = -1281.401221964

W -0.1586049 3.1045075 0.0000042

F 1.6312811 2.4650457 0.0000192

F 0.5512933 3.8462831 -1.5565952

F 0.5512648 3.8462773 1.5566194

F -1.4979795 2.5891587 1.2318291

F -1.1015211 4.7152821 -0.0000010

F -1.4979589 2.5891654 -1.2318459

C -0.1097903 0.8371142 -0.0000005

N -0.0738040 0.0505757 1.0879338

N -0.0737845 0.0505757 -1.0879344

C 0.0565330 0.5212802 2.4760191

C -0.0508650 -1.2814765 0.6978692

C -0.0508486 -1.2814765 -0.6978691

C 0.0565293 0.5212794 -2.4760224

H 0.0947340 1.6045315 2.4149197

C -1.1665200 0.1548101 3.2958898

C 1.3741922 0.0585586 3.0744890

C -0.0392343 -2.4703139 1.4174987

C -0.0391914 -2.4703137 -1.4174983

H 0.0947087 1.6045314 -2.4149265

C -1.1665269 0.1547815 -3.2958761

C 1.3741902 0.0585830 -3.0745080

H -2.0654712 0.5598249 2.8312802

H -1.0724321 0.5892730 4.2923188

H -1.2844801 -0.9236218 3.4101292

H 1.4244459 -1.0225760 3.2051111

H 1.4963114 0.5209869 4.0551168

H 2.2096804 0.3714394 2.4462584

H -0.0476638 -2.4861592 2.4971937

C -0.0237913 -3.6502092 0.6981243

H -0.0475917 -2.4861591 -2.4971936

C -0.0237652 -3.6502090 -0.6981236

H -1.0724622 0.5892450 -4.2923070

H -2.0654806 0.5597773 -2.8312547

H -1.2844650 -0.9236532 -3.4101120

H 1.4244651 -1.0225510 -3.2051270

H 2.2096804 0.3714840 -2.4462902

H 1.4962860 0.5210101 -4.0551392

H -0.0157099 -4.5933314 1.2303225

H -0.0156531 -4.5933311 -1.2303216

[(IMes)WF_6_] **3**

Energy = -1589.630490486

W 0.2776637 0.1819155 0.1557706

C 0.2407856 0.0182882 -2.1736824

N -0.8408804 -0.0269994 -2.9861213

N 1.2957878 -0.0497406 -3.0188917

C -0.4660659 -0.1205659 -4.3075351

C -2.2379156 0.0049440 -2.6544316

C 0.8785667 -0.1343426 -4.3281971

C 2.7031250 -0.0479614 -2.7318712

H -1.1981005 -0.1675914 -5.0948054

C -2.9078010 -1.2029165 -2.4791853

C -2.8931891 1.2332726 -2.6509324

H 1.5849770 -0.1950016 -5.1376327

C 3.3846594 1.1657654 -2.7469930

C 3.3523677 -1.2707628 -2.5828992

C -4.2695607 -1.1483169 -2.2142065

C -2.1998626 -2.5167225 -2.5874317

C -4.2555282 1.2326346 -2.3821166

C -2.1700466 2.5107148 -2.9415963

C 4.7549684 1.1346358 -2.5243953

C 2.6804064 2.4597122 -3.0094007

C 4.7233808 -1.2466652 -2.3650751

C 2.6127548 -2.5686564 -2.6698502

C -4.9560972 0.0573155 -2.1431920

H -4.8080318 -2.0783247 -2.0625255

H -2.9124316 -3.3387164 -2.5237713

H -1.6641590 -2.6069360 -3.5361912

H -1.4688802 -2.6340623 -1.7862498

H -4.7826129 2.1810838 -2.3623912

H -2.8733089 3.3412251 -2.9974799

H -1.4393918 2.7320615 -2.1624577

H -1.6312661 2.4600451 -3.8915696

C 5.4377755 -0.0563375 -2.3131882

H 5.3030736 2.0713124 -2.5210561

H 1.9867288 2.6977861 -2.2018710

H 3.4001084 3.2735819 -3.0937809

H 2.1030675 2.4219101 -3.9370475

H 5.2468153 -2.1887969 -2.2375772

H 1.9058993 -2.6716447 -1.8454047

H 2.0445007 -2.6451140 -3.6007245

H 3.3088896 -3.4061126 -2.6308155

C -6.4150064 0.0892023 -1.8075216

C 6.9073104 -0.0572024 -2.0257988

H -6.9069449 0.9573126 -2.2492085

H -6.9243473 -0.8106006 -2.1566646

H -6.5578094 0.1469434 -0.7242122

H 7.0874273 -0.0125453 -0.9474847

H 7.3859769 -0.9639334 -2.3996555

H 7.4021395 0.8046811 -2.4763660

F 1.6439547 1.3299741 -0.4078135

F 0.3131039 1.4439959 1.5347690

F 0.2891068 -0.8607132 1.7080134

F -1.0956920 -1.0404257 -0.1943736

F 1.5984749 -1.0845827 -0.2396679

F -1.0649661 1.3743776 -0.3735418

[(IDipp)WF_6_] **4**

Energy = -1825.310774043

W -1.3023491 0.0267458 -0.0504465

F -2.7157382 0.3775005 1.1292852

F -0.3817935 -1.1066398 -1.2257656

F -2.6909546 0.3318231 -1.2730795

F -0.5178810 1.2513777 -1.2222534

F -0.5337210 1.2807120 1.1011219

F -0.4089361 -1.0739827 1.1767463

C -2.5242956 -1.9300772 -0.0306972

N -2.9758929 -2.6094893 1.0456321

N -2.9405678 -2.6544018 -1.0918027

C -3.6631024 -3.7394756 0.6614720

C -2.8281214 -2.2900177 2.4400127

C -3.6404627 -3.7680331 -0.6832849

C -2.7454646 -2.3956618 -2.4929814

H -4.0976359 -4.4059098 1.3846349

C -1.7433525 -2.8318400 3.1324519

C -3.8298518 -1.5353384 3.0544796

H -4.0500195 -4.4653789 -1.3917287

C -3.7257527 -1.6707217 -3.1739235

C -1.6377569 -2.9660945 -3.1235435

C -1.6611425 -2.5623376 4.4935322

C -0.7230758 -3.7250320 2.4645868

C -3.6986950 -1.2967909 4.4172799

C -5.0440235 -1.0403006 2.3022375

C -3.5485362 -1.4930843 -4.5409427

C -4.9650161 -1.1447173 -2.4864401

C -1.5097958 -2.7580021 -4.4917430

C -0.6400130 -3.8259111 -2.3818735

C -2.6239375 -1.7990551 5.1283925

H -0.8278489 -2.9545157 5.0631584

H -0.7758109 -3.5479400 1.3886728

H -4.4490886 -0.7061135 4.9277389

H -4.8017524 -1.0402901 1.2377384

C -2.4506142 -2.0252202 -5.1922590

H -4.2812336 -0.9268916 -5.1023255

H -4.7600549 -1.0989427 -1.4151409

H -0.6580945 -3.1745468 -5.0149451

H -0.7289180 -3.6001044 -1.3174762

H -2.5371771 -1.5954928 6.1895044

H -2.3282145 -1.8693894 -6.2579367

C 0.8017196 -3.5375940 -2.7818279

H 1.4827476 -4.0950129 -2.1346251

H 1.0063454 -3.8484267 -3.8093143

H 1.0310524 -2.4767101 -2.6873218

C -0.9645744 -5.3059168 -2.5827441

H -1.9736078 -5.5510324 -2.2452525

H -0.8918651 -5.5762029 -3.6394406

H -0.2616609 -5.9295005 -2.0247291

C -6.1374323 -2.1005326 -2.7054405

H -7.0273902 -1.7376664 -2.1851625

H -6.3761071 -2.1801721 -3.7692122

H -5.9181205 -3.1047846 -2.3374015

C -5.3358475 0.2644514 -2.9315884

H -5.6693505 0.2852521 -3.9719992

H -6.1605990 0.6392373 -2.3210469

H -4.4924868 0.9456219 -2.8229041

C -1.0576343 -5.1941023 2.7216606

H -0.3380502 -5.8430751 2.2163007

H -1.0207675 -5.4160269 3.7913717

H -2.0555041 -5.4534266 2.3622415

C 0.7050297 -3.4198023 2.8992748

H 0.8743144 -3.6837387 3.9461340

H 1.4061615 -4.0066044 2.3013769

H 0.9398458 -2.3644993 2.7643578

C -5.4298017 0.3862518 2.6729725

H -6.2325114 0.7332645 2.0182295

H -5.7997278 0.4514884 3.6991337

H -4.5830201 1.0632094 2.5647621

C -6.2238955 -1.9870217 2.5209031

H -5.9922948 -3.0059970 2.2045488

H -6.4996869 -2.0208240 3.5781256

H -7.0948879 -1.6479259 1.9545283

[(cAAC^Me^)WF_6_] **6**

Energy = -1501.026721389

W 3.1454639 4.8696518 14.2032290

F 1.3512780 4.1981773 14.3749900

F 3.2974640 4.8398668 16.0471796

F 2.9379477 4.7794430 12.3712095

F 4.8874702 4.0619354 14.2552632

F 4.3479844 6.2643436 13.8614383

F 2.0607960 6.3848522 14.3750962

C 3.0612703 2.6153955 14.1307706

N 3.0764190 1.8315178 13.0989614

C 3.0315936 1.7963229 15.4169834

C 3.0635044 2.1893013 11.6931797

C 3.1294499 0.3469114 13.4343917

C 2.7660972 0.3721295 14.9077456

C 1.9111474 2.1951230 16.3777356

C 4.3806188 1.8937604 16.1401445

C 1.8195264 2.2801260 11.0477551

C 4.2699954 2.3676032 11.0000930

C 4.5259037 -0.1897360 13.1747108

C 2.1375001 -0.4600502 12.6229728

H 3.3294934 -0.3697250 15.4744029

H 1.7046544 0.1418305 15.0220713

H 1.8781322 1.4493824 17.1765186

H 0.9435768 2.2059021 15.8759763

H 2.0739320 3.1697241 16.8296068

H 4.3566350 1.2144354 16.9953803

H 4.5665157 2.9012941 16.5046555

H 5.2148848 1.6110678 15.4981664

C 1.8166448 2.4680064 9.6710022

C 0.4821461 2.2746003 11.7591503

C 5.6306436 2.5029169 11.6514549

C 4.1997950 2.5445928 9.6214066

H 4.5589051 -1.2335759 13.4918342

H 5.2927759 0.3574025 13.7210508

H 4.7581976 -0.1565710 12.1117955

H 2.2259006 -1.5052266 12.9265466

H 2.3400822 -0.4002687 11.5530876

H 1.1144033 -0.1430338 12.8121910

C 2.9923386 2.5756135 8.9549013

H 0.8676956 2.5434589 9.1542521

C -0.4918632 1.2519381 11.1752041

H 0.6445755 2.0421188 12.8134623

C -0.1559154 3.6632160 11.6942439

H 5.5289446 2.3147023 12.7197607

C 6.6716579 1.5442643 11.0777947

C 6.1405932 3.9372101 11.4901597

H 5.1191307 2.6843080 9.0659491

H 2.9658368 2.7142991 7.8803966

H -1.3907750 1.1986658 11.7938511

H -0.0644315 0.2515088 11.1037624

H -0.8090498 1.5448869 10.1719377

H -1.0896653 3.6711669 12.2611031

H -0.3870148 3.9278472 10.6593013

H 0.4952672 4.4298308 12.1059313

H 7.6101919 1.6470639 11.6273299

H 6.8830550 1.7759018 10.0314536

H 6.3655012 0.4991404 11.1268546

H 7.0624028 4.0707089 12.0600008

H 5.4209560 4.6728485 11.8410958

H 6.3578202 4.1497722 10.4402100

[(I*i*Pr^Me^)WF_5_] **7**

Energy = -1106.637483948

W -1.6267243 -1.1941965 -0.0744174

C -1.6259270 -1.1929793 2.1726182

N -2.3495698 -1.9868274 2.9934455

N -0.9012982 -0.3988858 2.9922803

C -2.0865275 -1.6929972 4.3162294

C -3.3157012 -2.9824671 2.5027698

C -1.1626905 -0.6922397 4.3154691

C 0.0640113 0.5967036 2.4999910

H -3.1314308 -3.0346717 1.4352745

C -4.7452400 -2.5118537 2.7065460

C -3.0437449 -4.3718482 3.0524937

H -0.1214864 0.6482879 1.4326792

C 1.4939503 0.1267177 2.7023947

C -0.2077042 1.9862763 3.0493650

H -5.4239423 -3.2123736 2.2167641

H -5.0253394 -2.4610504 3.7599887

H -4.8909959 -1.5305796 2.2546662

H -3.3390321 -4.4853625 4.0953667

H -3.6185281 -5.0919881 2.4680106

H -1.9881866 -4.6285022 2.9499319

H 2.1717867 0.8269168 2.2109643

H 1.7754782 0.0771804 3.7555063

H 1.6394403 -0.8549835 2.2513787

H -1.2633973 2.2426924 2.9476766

H 0.0885128 2.1002084 4.0919287

H 0.3664492 2.7063159 2.4641465

F 0.2164497 -1.3001266 0.0891964

F -1.5649489 0.7120653 -0.1189931

F -3.4697998 -1.0878567 0.0907722

F -1.6878531 -3.1005584 -0.1162917

F -1.6283261 -1.1954304 -1.9651781

C -2.6912522 -2.3663283 5.4926481

H -2.5177173 -1.7661709 6.3847201

H -3.7686638 -2.4890491 5.3897647

H -2.2581011 -3.3541733 5.6659491

C -0.5564119 -0.0187023 5.4909657

H 0.5209276 0.1035496 5.3868461

H -0.9889846 0.9693332 5.6645340

H -0.7291816 -0.6185450 6.3833989

[(BI*i*Pr)WF_5_] **8**

Energy = -1181.596202720

W -0.4328576 -0.9408247 0.0189549

F -0.2711536 -0.7640637 -1.8150668

F -0.5872001 -0.7080531 1.8478144

F 1.4666591 -1.0112902 0.1698078

F -2.3334251 -0.9558039 -0.1349866

F -0.4630983 -2.8266620 0.0535588

C -0.4095732 1.3115488 -0.0048043

N -1.1782576 2.1170933 -0.7710381

N 0.3587660 2.1166738 0.7621552

C -2.1741562 1.6532576 -1.7467739

C -0.9050122 3.4451660 -0.4961730

C 0.0785644 3.4448629 0.4951593

C 1.3648516 1.6515572 1.7268013

H -2.1471755 0.5709521 -1.6901574

C -3.5735628 2.0824944 -1.3417339

C -1.7794354 2.0558681 -3.1569382

C -1.4141517 4.6363674 -1.0039477

C 0.5778466 4.6356823 1.0134860

H 1.3530140 0.5698228 1.6522118

C 0.9725778 2.0282673 3.1445950

C 2.7567094 2.1059449 1.3221438

H -3.8035845 1.7197974 -0.3391247

H -4.2930281 1.6398594 -2.0322828

H -3.7098660 3.1641905 -1.3621642

H -1.7874670 3.1363198 -3.3056721

H -2.4840786 1.6149290 -3.8637317

H -0.7831172 1.6803727 -3.3943227

H -2.1730876 4.6529792 -1.7718554

C -0.9136473 5.8152304 -0.4875591

H 1.3346668 4.6517243 1.7834897

C 0.0675515 5.8148956 0.5075787

H 1.6897243 1.5911239 3.8411399

H -0.0151432 1.6317822 3.3830754

H 0.9626588 3.1068971 3.3065348

H 2.8824097 3.1880487 1.3689349

H 2.9840287 1.7719324 0.3089066

H 3.4856967 1.6537709 1.9962999

H -1.2912111 6.7588404 -0.8616778

H 0.4361644 6.7582452 0.8911622

[(IMes)WF_5_] **9**

Energy = -1489.821911545

W 0.3861174 -1.2631421 0.1068911

F 0.2823938 -1.1572778 -1.7344501

F -1.5087277 -1.4255922 0.1996124

F 0.4684787 -1.0062751 1.9346349

F 2.2872213 -1.2036354 0.0154462

F 0.5003596 -3.1455481 0.1839264

C 0.2510347 0.9798227 0.0245965

N -0.5099163 1.7952112 0.7918486

N 0.9150586 1.8246917 -0.7985703

C -0.3221629 3.1152739 0.4562809

C -1.4279833 1.4003582 1.8186489

C 0.5745816 3.1336439 -0.5508769

C 1.8709084 1.4726163 -1.8063172

H -0.8468403 3.9064827 0.9630768

C -2.7444353 1.1205915 1.4643997

C -0.9809187 1.3756500 3.1374670

H 1.0058462 3.9445821 -1.1117209

C 3.2118809 1.3766143 -1.4501885

C 1.4247484 1.3073881 -3.1163908

C -3.6191090 0.7656758 2.4831190

C -3.2049630 1.1859821 0.0428131

C -1.8943734 1.0138257 4.1171430

C 0.4319538 1.7217371 3.4885982

C 4.1197450 1.0649039 -2.4553798

C 3.6675221 1.5880544 -0.0413804

C 2.3709872 0.9976784 -4.0813014

C -0.0216702 1.4540361 -3.4709389

C -3.2113633 0.6939040 3.8085360

H -4.6485215 0.5353374 2.2285951

H -4.2931579 1.1533771 -0.0067588

H -2.8680412 2.0994915 -0.4529124

H -2.8130104 0.3358787 -0.5193250

H -1.5647517 0.9782907 5.1505020

H 0.5779491 1.6785153 4.5675029

H 1.1314221 1.0251045 3.0228680

H 0.6970811 2.7274805 3.1517343

C 3.7194673 0.8618348 -3.7683910

H 5.1702777 0.9766723 -2.1986520

H 3.3858354 0.7335136 0.5772242

H 4.7516549 1.6926509 -0.0034716

H 3.2226409 2.4826260 0.4006572

H 2.0440120 0.8557253 -5.1064669

H -0.6270367 0.7054776 -2.9562985

H -0.4084378 2.4376463 -3.1911178

H -0.1658985 1.3280408 -4.5435664

C -4.1627756 0.2582339 4.8794870

C 4.7108272 0.4888449 -4.8268401

H -3.9263280 0.7247843 5.8372470

H -5.1938580 0.5041791 4.6208407

H -4.1091893 -0.8255010 5.0203760

H 4.6306358 -0.5743849 -5.0710643

H 4.5372105 1.0477923 -5.7488243

H 5.7336180 0.6771355 -4.4984689

[(IDipp)WF_5_] **10**

Energy = -1725.499373467

W 0.4050223 -1.1712088 0.1301621

F 0.2600268 -1.0836839 -1.7092863

F -1.4854643 -1.3344922 0.2720006

F 0.5257000 -0.8793649 1.9507705

F 2.3017938 -1.1059319 -0.0105337

F 0.5262364 -3.0523887 0.2336197

C 0.2690963 1.0622355 0.0224986

N -0.4989988 1.8840810 0.7748406

N 0.9467952 1.8984310 -0.7979617

C -0.3017471 3.2004242 0.4331259

C -1.4374609 1.4971455 1.7899952

C 0.6079839 3.2092586 -0.5623242

C 1.9021751 1.5323556 -1.8051267

H -0.8280655 3.9982528 0.9258329

C -2.7463352 1.2021322 1.4018341

C -1.0085271 1.4876106 3.1182165

H 1.0458389 4.0165408 -1.1218109

C 3.2458957 1.4293488 -1.4393134

C 1.4444112 1.3501125 -3.1116502

C -3.6481979 0.8858271 2.4103286

C -3.1859073 1.2644701 -0.0430970

C -1.9521755 1.1674639 4.0871046

C 0.4088339 1.8405915 3.5074575

C 4.1538635 1.1290698 -2.4475246

C 3.7108418 1.6749790 -0.0222438

C 2.3949846 1.0537521 -4.0810528

C -0.0144250 1.4989691 -3.4793684

C -3.2568076 0.8703605 3.7372754

H -4.6701182 0.6396092 2.1517660

H -2.3029766 1.1006028 -0.6652411

H -1.6579463 1.1416485 5.1289374

H 1.0367627 1.7273611 2.6203793

C 3.7342793 0.9451309 -3.7528792

H 5.2040812 1.0293786 -2.2047348

H 2.8665600 1.4767324 0.6422705

H 2.0802555 0.8971234 -5.1052591

H -0.6023563 1.3850144 -2.5653543

H 4.4593470 0.7066255 -4.5225060

H -3.9760410 0.6159739 4.5073228

C -3.7475730 2.6481846 -0.3699001

H -4.0372644 2.7029022 -1.4222503

H -4.6335504 2.8545850 0.2363471

H -3.0206039 3.4399915 -0.1797673

C -4.1918612 0.1809288 -0.4075809

H -5.1564123 0.3425133 0.0803437

H -4.3710410 0.1946892 -1.4851110

H -3.8179383 -0.8052730 -0.1347150

C 0.4920404 3.2970938 3.9625835

H 0.1485486 3.9863041 3.1886887

H -0.1265923 3.4578097 4.8493717

H 1.5219660 3.5601063 4.2160172

C 0.9743776 0.9147881 4.5776585

H 2.0325454 1.1357809 4.7352594

H 0.4697211 1.0511716 5.5371469

H 0.8830255 -0.1301542 4.2828496

C 4.1249173 3.1356185 0.1565613

H 4.4346671 3.3202297 1.1882208

H 4.9662172 3.3780597 -0.4981044

H 3.3102781 3.8234418 -0.0781113

C 4.8391676 0.7449662 0.4034416

H 5.7669540 0.9620240 -0.1319463

H 5.0437597 0.8799510 1.4679886

H 4.5690862 -0.2969836 0.2357339

C -0.2851887 2.8933339 -4.0432287

H -0.0092223 3.6790780 -3.3373126

H 0.2870643 3.0538333 -4.9606698

H -1.3455030 3.0102825 -4.2805532

C -0.4907842 0.4283299 -4.4533400

H -1.5718259 0.5080226 -4.5891462

H -0.0324308 0.5453343 -5.4383060

H -0.2650790 -0.5707858 -4.0817401

[(cAAC^Me^)WF_5_]_2_ **12** triplet

Energy = -2802.425733228

W 0.0061609 0.2407536 -0.0942665

F 1.2850938 -1.1495058 0.1127442

F 1.3000423 1.4221618 -0.7130307

F -1.4712765 -0.8539550 -0.3471359

F -1.1873144 1.7124645 -0.1226158

F -0.7287125 -0.3743677 1.7834361

F 0.9227485 1.1621899 1.5859147

W 0.3046011 0.4021331 3.4276289

F 1.6499061 1.6494177 3.8191299

F 1.5492926 -1.0068480 3.1618631

F -0.9876564 -0.7727109 4.1243506

F -1.0081436 1.7813467 3.4705418

C 0.6010643 0.3416436 5.6232724

C -0.1087592 1.3186590 6.5383393

N 1.3061830 -0.4926308 6.3214495

C 0.5179525 1.0491371 7.9125801

C 0.1165732 2.7754219 6.1355652

C -1.6117585 1.0116228 6.5201109

C 1.2155300 -0.3006936 7.8265719

C 2.1524917 -1.5677026 5.8413253

H 1.2534449 1.8253707 8.1343167

H -0.2270023 1.0633981 8.7089467

H 1.1807309 3.0048395 6.0604116

H -0.3554123 3.0103821 5.1844225

H -0.3191783 3.4138480 6.9088634

H -2.0351017 1.1910413 5.5335435

H -1.8220328 -0.0222616 6.7987128

H -2.1068929 1.6698293 7.2381232

C 2.5850824 -0.2675830 8.4736701

C 0.4045825 -1.4261797 8.4426752

C 3.5066971 -1.2876457 5.6002784

C 1.6334980 -2.8636932 5.7018302

H 2.4502254 -0.1045811 9.5449990

H 3.1240781 -1.2058708 8.3374984

H 3.1876045 0.5505627 8.0838524

H 0.9194314 -2.3787165 8.3269044

H 0.2962213 -1.2306637 9.5111075

H -0.5907900 -1.5057214 8.0069480

C 4.3515333 -2.3558450 5.3238770

C 4.0880467 0.1079492 5.5168079

C 2.5314390 -3.8918105 5.4318538

C 0.1579224 -3.2051672 5.6802022

C 3.8803106 -3.6522449 5.2686930

H 5.3996651 -2.1593224 5.1336990

H 3.3232853 0.8271314 5.8166677

C 5.3148119 0.2993977 6.4064542

C 4.4655481 0.4202327 4.0669156

H 2.1515911 -4.9002756 5.3215552

H -0.4137885 -2.3070481 5.9138410

C -0.2316760 -4.3080411 6.6610208

C -0.2348891 -3.6391714 4.2644839

H 4.5590327 -4.4714917 5.0615565

H 5.6341620 1.3439676 6.3794006

H 5.1327949 0.0234580 7.4454641

H 6.1530920 -0.3030072 6.0491649

H 4.7685926 1.4655262 3.9746185

H 5.3051158 -0.2052235 3.7520653

H 3.6373047 0.2411772 3.3857665

H 0.0148210 -4.0692303 7.6954998

H -1.3080863 -4.4869337 6.6043349

H 0.2656591 -5.2481660 6.4113712

H -1.3206545 -3.7258384 4.1865020

H 0.1060163 -2.9284363 3.5158003

H 0.2012699 -4.6142260 4.0305283

C 0.1286230 -0.2871478 -2.2555025

C 1.0586522 0.4108789 -3.2569952

N -0.4387471 -1.3060767 -2.8553144

C 0.7353362 -0.2850897 -4.5860399

C 0.6914950 1.8929054 -3.3936033

C 2.5466973 0.2496400 -2.9213446

C 0.1244312 -1.6215143 -4.2307259

C -1.5588571 -2.1292527 -2.4472846

H 0.0015650 0.3057112 -5.1406926

H 1.6184801 -0.3935845 -5.2161679

H -0.3856761 2.0149094 -3.5332021

H 0.9953563 2.4844564 -2.5364431

H 1.1888688 2.2849944 -4.2852901

H 2.8184569 0.7576471 -2.0023892

H 2.8324152 -0.7978179 -2.8251076

H 3.1284806 0.6803750 -3.7401643

C -0.9269518 -2.0554401 -5.2336690

C 1.1529397 -2.7361664 -4.1022498

C -2.8346755 -1.6810255 -2.8241440

C -1.3664155 -3.3518864 -1.7950850

H -0.4213241 -2.2247815 -6.1869374

H -1.4169223 -2.9839579 -4.9400662

H -1.6834623 -1.2900662 -5.3905682

H 0.6673614 -3.6780562 -3.8564236

H 1.6565614 -2.8594816 -5.0629201

H 1.9042268 -2.5264133 -3.3431486

C -3.9136550 -2.5221694 -2.5881486

C -3.0995856 -0.3043522 -3.3991379

C -2.4841611 -4.1593210 -1.6014821

C -0.0605233 -3.7918825 -1.1696547

C -3.7432125 -3.7624944 -2.0020895

H -4.9083773 -2.1947157 -2.8643427

H -2.1543724 0.1100573 -3.7564219

C -4.0691591 -0.3290725 -4.5781816

C -3.6282531 0.6376891 -2.3175101

H -2.3598400 -5.1097791 -1.0964716

H 0.7113033 -3.0637620 -1.4216657

C 0.3895839 -5.1736297 -1.6385289

C -0.1914032 -3.8054118 0.3561988

H -4.5975694 -4.4080712 -1.8350503

H -4.1064312 0.6561578 -5.0486074

H -3.7870573 -1.0618533 -5.3368591

H -5.0843462 -0.5662416 -4.2526777

H -3.8188214 1.6266754 -2.7423079

H -4.5695909 0.2572534 -1.9125028

H -2.9300204 0.7517720 -1.4908675

H 0.4533391 -5.2585582 -2.7242110

H 1.3723423 -5.4061943 -1.2219250

H -0.3013123 -5.9461370 -1.2924730

H 0.7854444 -3.9725077 0.8141529

H -0.5859823 -2.8685064 0.7417534

H -0.8602791 -4.6095576 0.6750684

[(cAAC^Me^)WF_5_]_2_ singlet (dissociative)

Energy = -2802.418311376

W 0.0515150 0.8880518 -0.4086955

F 1.9077079 0.5297059 -0.1013884

F 0.4179440 2.6411837 -0.8848624

F -0.2594140 -0.6018508 0.6412901

F -1.7627305 1.4934999 -0.2649031

F 0.2204755 1.8188773 1.7956175

F 2.4485131 2.3555563 2.3408186

W 1.1796356 1.6012322 3.4720990

F 2.8302138 1.4564418 4.4228106

F 1.4107823 -0.1196442 2.8892553

F -0.6633066 1.3988094 3.8939415

F 0.9161336 3.2988020 4.1557433

C 0.9218163 0.6933202 5.5160292

C 0.8108722 1.5757812 6.7525951

N 0.8197350 -0.5563452 5.8396681

C 0.9469481 0.5654381 7.8996185

C 1.9031019 2.6342625 6.8931776

C -0.5652288 2.2561966 6.7535603

C 0.6344668 -0.8078246 7.3315192

C 0.8549260 -1.7126232 4.9604338

H 1.9747623 0.5803483 8.2683900

H 0.2912185 0.8148557 8.7343515

H 2.8963152 2.1920266 6.8181571

H 1.8208162 3.4258959 6.1534834

H 1.8008502 3.0853034 7.8836565

H -0.6566291 2.9706045 5.9382053

H -1.3807565 1.5382883 6.6621247

H -0.6804954 2.7916031 7.6985974

C 1.5944019 -1.8609099 7.8444449

C -0.7841816 -1.2616411 7.6216033

C 2.0980589 -2.3195056 4.7114056

C -0.3380774 -2.2188128 4.4198408

H 1.4210171 -1.9816317 8.9157166

H 1.4315639 -2.8262901 7.3641074

H 2.6306379 -1.5610512 7.7046803

H -0.9796989 -2.2285600 7.1627040

H -0.8908260 -1.3764692 8.7017404

H -1.5334330 -0.5495456 7.2801107

C 2.1058676 -3.4947725 3.9713327

C 3.4402519 -1.7446617 5.1186315

C -0.2579090 -3.3936315 3.6780827

C -1.6975531 -1.5535618 4.5063671

C 0.9413469 -4.0401201 3.4704615

H 3.0536819 -3.9766840 3.7649626

H 3.2738966 -0.8511876 5.7234932

C 4.2778649 -2.7366380 5.9258534

C 4.2408381 -1.3167575 3.8870055

H -1.1604248 -3.7917996 3.2318841

H -1.6120465 -0.6570752 5.1213022

C -2.7614629 -2.4707345 5.1095519

C -2.1686363 -1.1129828 3.1206644

H 0.9722561 -4.9542637 2.8902161

H 5.1824349 -2.2471943 6.2945404

H 3.7407147 -3.1525463 6.7781523

H 4.5964533 -3.5728501 5.2996054

H 5.1804789 -0.8547669 4.1982062

H 4.4811642 -2.1833322 3.2663479

H 3.7015003 -0.6037350 3.2697169

H -2.4772257 -2.8918816 6.0739085

H -3.6940583 -1.9174360 5.2425862

H -2.9741746 -3.3055974 4.4382027

H -3.1247667 -0.5909723 3.2033074

H -1.4617667 -0.4475593 2.6377631

H -2.3096788 -1.9750841 2.4647236

C -0.0460026 0.0332302 -2.1427939

C -0.2291949 0.8574434 -3.4431744

N 0.0799496 -1.2876569 -2.4745389

C -0.3624032 -0.2557185 -4.4969701

C -1.5069129 1.6937567 -3.4038618

C 0.9569642 1.7734799 -3.7476174

C 0.3111388 -1.4983401 -3.9282580

C -0.1882742 -2.3726524 -1.5831670

H -1.4214600 -0.4736075 -4.6588032

H 0.0661577 0.0473798 -5.4536813

H -2.3674669 1.0894955 -3.1108503

H -1.4288532 2.5235155 -2.7013602

H -1.6963125 2.1064891 -4.3990118

H 1.0369681 2.5761506 -3.0171993

H 1.9041232 1.2362456 -3.7601772

H 0.8109132 2.2268249 -4.7317701

C -0.3324062 -2.7761369 -4.4320907

C 1.8028025 -1.5396695 -4.2571972

C -1.5281527 -2.7378542 -1.3525121

C 0.8564743 -3.0704175 -0.9585901

H -0.1411911 -2.8713352 -5.5029735

H 0.0830174 -3.6543384 -3.9345868

H -1.4098619 -2.7694054 -4.2784199

H 2.2528499 -2.4685423 -3.9111572

H 1.9435627 -1.4833959 -5.3392429

H 2.3422537 -0.7133959 -3.7971510

C -1.7899037 -3.8265056 -0.5293892

C -2.7067852 -1.9918284 -1.9368901

C 0.5416358 -4.1662264 -0.1613820

C 2.3084165 -2.6701737 -1.0674723

C -0.7662756 -4.5502044 0.0516904

H -2.8188902 -4.1152450 -0.3478715

H -2.3198260 -1.2485647 -2.6354840

C -3.6546820 -2.9140187 -2.7013633

C -3.4799671 -1.2500788 -0.8484590

H 1.3462502 -4.7163131 0.3120730

H 2.3601357 -1.7548816 -1.6574969

C 3.1372911 -3.7547452 -1.7525013

C 2.8956274 -2.3622264 0.3089024

H -0.9909355 -5.4078860 0.6766574

H -4.4213265 -2.3248862 -3.2106592

H -3.1363371 -3.5195694 -3.4467983

H -4.1693424 -3.5997977 -2.0238753

H -4.3155685 -0.6993021 -1.2883403

H -3.8911865 -1.9569414 -0.1223449

H -2.8538645 -0.5367757 -0.3165128

H 2.7130786 -4.0594444 -2.7111989

H 4.1584414 -3.4053301 -1.9244409

H 3.1946188 -4.6493484 -1.1264083

H 3.9078215 -1.9642977 0.2036943

H 2.2987429 -1.6246797 0.8409952

H 2.9525501 -3.2664282 0.9211148

[(cAAC^Me^)_2_WF_5_] **13**

Energy = -2236.100220928

W 6.9372347 4.9952381 15.5565502

F 6.5030130 5.1212940 17.4214128

C 5.0391085 5.9044151 12.5482344

F 7.2925290 4.7588586 13.6857264

F 8.7877679 5.5531619 15.7440547

N 4.4536462 6.2185313 13.8345213

N 8.7592182 2.8647317 17.2218983

C 8.2132115 3.2298948 18.5120707

C 9.9384735 1.9072667 17.1686456

C 5.9711441 6.7610991 11.9468641

C 4.6085401 4.7275929 11.9139535

C 8.7146975 4.3251975 19.2282721

C 3.8596171 6.1866469 16.0603906

C 6.4324589 6.4274180 10.6772086

H 7.1576657 7.0754008 10.1997646

C 8.1886832 4.5674373 20.4934800

H 8.5607140 5.4134707 21.0587729

C 3.6548997 3.7223256 12.5274784

H 3.2685936 4.1329244 13.4624610

C 8.3193258 3.2502909 16.0592481

C 7.1885235 2.4213866 19.0307724

C 6.0118403 5.2855224 10.0281256

H 6.3924425 5.0456305 9.0419076

C 3.4881142 4.9497352 16.8796531

H 2.6212670 5.1932089 17.5005148

H 4.3061413 4.6447398 17.5279827

H 3.2216753 4.1093895 16.2350047

C 3.1834143 7.0519659 13.8782859

C 4.8885178 5.8291166 14.9977359

C 9.7934602 1.3806910 15.7522230

H 9.2088423 0.4567310 15.7691933

H 10.7608362 1.1540094 15.3022215

C 6.3115884 9.2509113 11.7753457

H 6.6327883 10.1346700 12.3318541

H 5.2618123 9.3799811 11.5066041

H 6.8814998 9.2269287 10.8430902

C 2.6473120 6.6542989 15.2417919

H 1.9440412 5.8251966 15.1248175

H 2.1160503 7.4756652 15.7243339

C 7.1931677 3.7779143 21.0299036

H 6.7963395 3.9929222 22.0154635

C 9.8562835 0.8112264 18.2105865

H 10.7511054 0.1911828 18.1250216

H 8.9915613 0.1702666 18.0558363

H 9.8218616 1.2165616 19.2230659

C 2.2229691 6.7233961 12.7543900

H 1.3580825 7.3844856 12.8405910

H 1.8653374 5.6983207 12.8147881

H 2.6752826 6.8851349 11.7746902

C 9.0274763 2.4577040 14.9702090

C 6.5663175 7.9891550 12.5997862

H 6.1229082 8.1080241 13.5867475

C 8.0722606 7.8258359 12.8069616

H 8.4647404 8.6893896 13.3488264

H 8.5916321 7.7666814 11.8464361

H 8.3059996 6.9335936 13.3813540

C 8.0062320 1.7987590 14.0418925

H 8.5322770 1.0914002 13.3944579

H 7.5057358 2.5357606 13.4183970

H 7.2505405 1.2483931 14.6064834

C 3.5338451 8.5307899 13.8039650

H 2.6245450 9.1118906 13.9701134

H 3.9212797 8.7919446 12.8215914

H 4.2674296 8.8193200 14.5547495

C 6.6942763 2.7216746 20.2941861

H 5.8971234 2.1137428 20.7051836

C 4.3682667 7.2754990 17.0104716

H 4.6581859 8.1836145 16.4839016

H 5.2293851 6.9252629 17.5737760

H 3.5650464 7.5215454 17.7102250

C 11.2365647 2.6753399 17.3696009

H 12.0727840 1.9957418 17.1934127

H 11.3177167 3.0462751 18.3889445

H 11.3282783 3.5168899 16.6852357

C 6.5535137 1.2615073 18.2906302

H 7.1127857 1.0868725 17.3692923

C 5.1152582 4.4414071 10.6522189

H 4.8004053 3.5329177 10.1526852

C 9.7398506 5.3066659 18.7044728

H 10.0401642 4.9961416 17.7054683

C 5.1165608 1.5906651 17.8930719

H 4.6743033 0.7484112 17.3537954

H 5.0755416 2.4657605 17.2505021

H 4.5066098 1.7781017 18.7806168

C 9.9623792 3.3371365 14.1338626

H 10.4759172 2.6987208 13.4098512

H 10.7116225 3.8418815 14.7419460

H 9.4003603 4.0960396 13.5957542

C 6.5681702 -0.0258103 19.1147016

H 6.2401401 -0.8679321 18.5001463

H 5.8795233 0.0447351 19.9597101

H 7.5554043 -0.2564813 19.5167995

C 9.1418063 6.7075159 18.5724576

H 9.8749482 7.3792363 18.1201228

H 8.8768480 7.1102807 19.5539847

H 8.2530564 6.7091804 17.9472287

C 10.9730994 5.3747806 19.6051032

H 11.7480194 5.9855897 19.1357836

H 11.3975367 4.3930297 19.8220096

H 10.7279144 5.8379507 20.5641224

C 2.4713206 3.4160986 11.6098060

H 1.7342627 2.8072106 12.1393517

H 2.7934465 2.8454486 10.7357871

H 1.9767792 4.3176881 11.2464725

C 4.3792601 2.4223048 12.8687149

H 3.6805151 1.7100868 13.3163009

H 5.1880325 2.5945423 13.5733741

H 4.7918460 1.9648631 11.9657087

F 5.8064361 3.4544443 15.4657155

F 6.9175181 6.9368973 15.5145273

[1] J. Attner, U. Radius, "Syntheses and Molecular Structures of New Calix[4]arene Molybdenum and Tungsten Complexes" *Chem. Eur. J.* **2001**, *7*, 783–790.

[2] G. R. Fulmer, A. J. M. Miller, N. H. Sherden, H. E. Gottlieb, A. Nudelman, B. M. Stoltz, J. E. Bercaw, K. I. Goldberg, "NMR Chemical Shifts of Trace Impurities: Common Laboratory Solvents, Organics, and Gases in Deuterated Solvents Relevant to the Organometallic Chemist" *Organometallics* **2010**, *29*, 2176–2179.

[3] S. Stoll, A. Schweiger, "EasySpin, a comprehensive software package for spectral simulation and analysis in EPR" *J. Magn. Reson.* **2006**, *178*, 42–55.

[4] C. L. L. C. Wilfred L. F. Armarego, *Purification of Laboratory Chemicals*, 5, Butterworth-Heinemann (Elsevier), Amsterdam, **2003**.

[5] N. Kuhn, T. Kratz, "Synthesis of Imidazol-2-Ylidenes by Reduction of Imidazole-2-(3H)-Thiones" *Synthesis* **1993**, *1993*, 561–562.

[6] A. J. Arduengo, III, H. V. R. Dias, R. L. Harlow, M. Kline, "Electronic stabilization of nucleophilic carbenes" *J. Am. Chem. Soc.* **1992**, *114*, 5530–5534.

[7] A. J. Arduengo, R. Krafczyk, R. Schmutzler, H. A. Craig, J. R. Goerlich, W. J. Marshall, M. Unverzagt, "Imidazolylidenes, Imidazolinylidenes and Imidazolidines" *Tetrahedron* **1999**, *55*, 14523–14534.

[8] C. M. Crudden, J. H. Horton, Ebralidze, II, O. V. Zenkina, A. B. McLean, B. Drevniok, Z. She, H. B. Kraatz, N. J. Mosey, T. Seki, E. C. Keske, J. D. Leake, A. Rousina-Webb, G. Wu, "Ultra stable self-assembled monolayers of *N*-heterocyclic carbenes on gold" *Nat. Chem.* **2014**, *6*, 409–414.

[9] O. V. Starikova, G. V. Dolgushin, L. I. Larina, T. N. Komarova, V. A. Lopyrev, "Synthesis of new stable carbenes from the corresponding 1,3-dialkylimidazolium and benzimidazolium salts" *Arkivoc* **2003**, *2003*, 119–124.

[10] W.-C. Chen, Y.-C. Lai, W.-C. Shih, M.-S. Yu, G. P. A. Yap, T.-G. Ong, "Mechanistic Study of a Switch in the Regioselectivity of Hydroheteroarylation of Styrene Catalyzed by Bimetallic Ni–Al through C–H Activation" *Chem. Eur. J.* **2014**, *20*, 8099–8105.

[11] V. Lavallo, Y. Canac, C. Präsang, B. Donnadieu, G. Bertrand, "Stable Cyclic (Alkyl)(Amino)Carbenes as Rigid or Flexible, Bulky, Electron-Rich Ligands for Transition-Metal Catalysts: A Quaternary Carbon Atom Makes the Difference" *Angew. Chem. Int. Ed. Engl.* **2005**, *44*, 5705–5709.

[12] C. Müller, D. M. Andrada, I.-A. Bischoff, M. Zimmer, V. Huch, N. Steinbrück, A. Schäfer, "Synthesis, Structure, and Bonding Analysis of Tin(II) Dihalide and Cyclopentadienyltin(II) Halide (Alkyl)(Amino)Carbene Complexes" *Organometallics* **2019**, *38*, 1052–1061.

[13] R. A. Sulzbach, A. F. M. Iqbal, "1,4-Bis(trimethylsilyl)-1,4-dihydropyrazine by Reductive Silylation of Pyrazine" *Angew. Chem. Int. Ed. Engl.* **1971**, *10*, 127–127.

[14] W. Kaim, "Effects of cyclic 8π-electron conjugation in reductively silylated nitrogen heterocycles" *J. Am. Chem. Soc.* **1983**, *105*, 707–713.

[15] G. Sheldrick, "SHELXT - Integrated Space-Group and Crystal-Structure Determination" *Acta Crystallogr., Sect. A* **2015**, *71*, 3–8.

[16] S. G. Balasubramani, G. P. Chen, S. Coriani, M. Diedenhofen, M. S. Frank, Y. J. Franzke, F. Furche, R. Grotjahn, M. E. Harding, C. Hättig, A. Hellweg, B. Helmich-Paris, C. Holzer, U. Huniar, M. Kaupp, A. Marefat Khah, S. Karbalaei Khani, T. Müller, F. Mack, B. D. Nguyen, S. M. Parker, E. Perlt, D. Rappoport, K. Reiter, S. Roy, M. Rückert, G. Schmitz, M. Sierka, E. Tapavicza, D. P. Tew, C. van Wüllen, V. K. Voora, F. Weigend, A. Wodyński, J. M. Yu, "TURBOMOLE: Modular program suite for ab initio quantum-chemical and condensed-matter simulations" *J. Chem. Phys.* **2020**, *152*.

[17] Y. J. Franzke, C. Holzer, J. H. Andersen, T. Begušić, F. Bruder, S. Coriani, F. Della Sala, E. Fabiano, D. A. Fedotov, S. Fürst, S. Gillhuber, R. Grotjahn, M. Kaupp, M. Kehry, M. Krstić, F. Mack, S. Majumdar, B. D. Nguyen, S. M. Parker, F. Pauly, A. Pausch, E. Perlt, G. S. Phun, A. Rajabi, D. Rappoport, B. Samal, T. Schrader, M. Sharma, E. Tapavicza, R. S. Treß, V. Voora, A. Wodyński, J. M. Yu, B. Zerulla, F. Furche, C. Hättig, M. Sierka, D. P. Tew, F. Weigend, "TURBOMOLE: Today and Tomorrow" *Journal of Chemical Theory and Computation* **2023**, *19*, 6859–6890.

[18] M. Häser, R. Ahlrichs, "Improvements on the direct SCF method" *J. Comput. Chem.* **1989**, *10*, 104–111.

[19] O. Treutler, R. Ahlrichs, "Efficient molecular numerical integration schemes" *J. Chem. Phys.* **1995**, *102*, 346–354.

[20] M. Sierka, A. Hogekamp, R. Ahlrichs, "Fast evaluation of the Coulomb potential for electron densities using multipole accelerated resolution of identity approximation" *J. Chem. Phys.* **2003**, *118*, 9136–9148.

[21] M. Ernzerhof, G. E. Scuseria, "Assessment of the Perdew–Burke–Ernzerhof exchange-correlation functional" *J. Chem. Phys.* **1999**, *110*, 5029–5036.

[22] C. Adamo, V. Barone, "Toward reliable density functional methods without adjustable parameters: The PBE0 model" *J. Chem. Phys.* **1999**, *110*, 6158–6170.

[23] A. Schäfer, C. Huber, R. Ahlrichs, "Fully optimized contracted Gaussian basis sets of triple zeta valence quality for atoms Li to Kr" *J. Chem. Phys.* **1994**, *100*, 5829–5835.

[24] K. Eichkorn, F. Weigend, O. Treutler, R. Ahlrichs, "Auxiliary basis sets for main row atoms and transition metals and their use to approximate Coulomb potentials" *Theor. Chem. Acc.* **1997**, *97*, 119–124.

[25] K. Eichkorn, O. Treutler, H. Öhm, M. Häser, R. Ahlrichs, "Auxiliary basis sets to approximate Coulomb potentials (Chem. Phys. Letters 240 (1995) 283-290)" *Chem. Phys. Lett.* **1995**, *242*, 652–660.

[26] F. Weigend, R. Ahlrichs, "Balanced basis sets of split valence, triple zeta valence and quadruple zeta valence quality for H to Rn: Design and assessment of accuracy" *Phys. Chem. Chem. Phys.* **2005**, *7*, 3297–3305.

[27] S. Grimme, J. Antony, S. Ehrlich, H. Krieg, "A consistent and accurate ab initio parametrization of density functional dispersion correction (DFT-D) for the 94 elements H-Pu" *J. Chem. Phys.* **2010**, *132*.

[28] S. Grimme, S. Ehrlich, L. Goerigk, "Effect of the damping function in dispersion corrected density functional theory" *J. Comput. Chem.* **2011**, *32*, 1456–1465.

[29] P. Deglmann, K. May, F. Furche, R. Ahlrichs, "Nuclear second analytical derivative calculations using auxiliary basis set expansions" *Chem. Phys. Lett.* **2004**, *384*, 103–107.

[30] A. E. Reed, R. B. Weinstock, F. Weinhold, "Natural population analysis" *J. Chem. Phys.* **1985**, *83*, 735–746.

[31] E. D. Glendening, C. R. Landis, F. Weinhold, "Erratum: NBO 6.0: Natural bond orbital analysis program" *J. Comput. Chem.* **2013**, *34*, 2134–2134.

[32] K. B. Wiberg, "Application of the pople-santry-segal CNDO method to the cyclopropylcarbinyl and cyclobutyl cation and to bicyclobutane" *Tetrahedron* **1968**, *24*, 1083–1096.

[33] F. Furche, R. Ahlrichs, "Erratum: “Time-dependent density functional methods for excited state properties” [J. Chem. Phys. 117, 7433 (2002)]" *J. Chem. Phys.* **2004**, *121*, 12772–12773.

[34] D. Rappoport, F. Furche, "Analytical time-dependent density functional derivative methods within the RI-J approximation, an approach to excited states of large molecules" *J. Chem. Phys.* **2005**, *122*, 064105.

[35] F. Furche, D. Rappoport, Density Functional Methods for Excited States: Equilibrium Structure and Electronic Spectra in M. Olivucci (ed.), *Computational Photochemistry*, *Vol. 16, Ch. 3.* (Eds.: M. Olivucci (ed.)), Elsevier, Amsterdam, **2005**.

[36] J.-D. Chai, M. Head-Gordon, "Systematic optimization of long-range corrected hybrid density functionals" *J. Chem. Phys.* **2008**, *128*.
